# Supplementary material for: Lipotrichaibol A and Trichoderpeptides A–D: Five New Peptaibiotics from a Sponge-Derived Trichoderma sp. GXIMD 01001
Source: Mar Drugs. 2025 Jun 24;23(7):264. doi: 10.3390/md23070264 (PMC12298237; doi:10.3390/md23070264)
Supplement: Supplementary file 1 [file marinedrugs-23-00264-s001.zip › marinedrugs-3688237-supplementary.pdf]

Supporting Information

Lipotrichaibol A and Trichoderpeptides A–D: Five New Peptaibiotics from a Sponge-Derived *Trichoderma* sp. GXIMD 01001

Weichan Yang [1,†], Zhenzhou Tang [1,†], Xiaowei Luo [1], Yuman Gan [1], Meng Bai [1], Houwen Lin [2], Chenghai Gao [1], Ling Chai [3,*], Xiao Lin [1,*]

1 Institute of Marine Drugs, Guangxi Key Laboratory of Marine Drugs, Guangxi University of Chinese Medicine, Nanning 530200, China; 15278363299@139.com (W.Y.); trcstrive2015@126.com (Z.T.); luoxiaowei1991@126.com (X.L.); gan_ym2018@163.com (Y.G.); xxbai2014@163.com (M.B.)
2 Research Center for Marine Drugs, Department of Pharmacy, Ren Ji Hospital, Shanghai Jiao Tong University School of Medicine, Shanghai 200127, China; franklin67@126.com
3 Guangxi Key Laboratory of Traditional Chinese Medicine Quality Standards, Guangxi Institute of Chinese Medicine and Pharmaceutical Science, Nanning 530022, China
* Correspondence: cicichai001@163.com (L.C.); linxiaolegend@163.com (X.L.)
† These authors contributed equally to this work.

Table Contents

Table S1. ^{1}H (800MHz) and ^{13}C (201MHz) NMR data of compound 3 in DMSO-d_6. 3
Table S2. ^{1}H (800MHz) and ^{13}C (201MHz) NMR data of compound 4 in DMSO-d_6. 4
Table S3. ^{1}H (600MHz) and ^{13}C (150MHz) NMR data of compound 5 in DMSO-d_6. 5

Contents

Fig S1. LC-MS and UV spectra of crude extract 6
Fig S2. ^{1}H NMR spectrum of compound 1 in DMSO-d_6 (600 MHz) 7
Fig S3. ^{13}C NMR spectrum of compound 1 in DMSO-d_6 (600 MHz) 8
Fig S4. DEPT-135 spectrum of compound 1 in DMSO-d_6 (600 MHz) 9
Fig S5. ^{1}H-^{1}H COSY spectrum of compound 1 in DMSO-d_6 (600 MHz) 10
Fig S6. HSQC spectrum of compound 1 in DMSO-d_6 (600 MHz) 11
Fig S7. HMBC spectrum of compound 1 in DMSO-d_6 (600 MHz) 12
Fig S8. TOCSY spectrum of compound 1 in DMSO-d_6 (600 MHz) 13
Fig S9. NOESY spectrum of compound 1 in DMSO-d_6 (600 MHz) 14
Fig S10. Marfey's of compound 1 15
Fig S11. CD spectrum of compound 1 in MeOH 16
Fig S12. ^{1}H NMR spectrum of compound 2 in DMSO-d_6 (600 MHz) 17
Fig S13. ^{13}C NMR spectrum of compound 2 in DMSO-d_6 (600 MHz) 18
Fig S14. DEPT-135 spectrum of compound 2 in DMSO-d_6 (600 MHz) 19
Fig S15. ^{1}H-^{1}H COSY spectrum of compound 2 in DMSO-d_6 (600 MHz) 20
Fig S16. HSQC spectrum of compound 2 in DMSO-d_6 (600 MHz) 21
Fig S17. HMBC spectrum of compound 2 in DMSO-d_6 (600 MHz) 22
Fig S18. TOCSY spectrum of compound 2 in DMSO-d_6 (600 MHz) 23
Fig S19. NOESY spectrum of compound 2 in DMSO-d_6 (600 MHz) 24
Fig S20. Marfey's of compound 2 25

Fig S21. CD spectrum of compound 2 in MeOH 26
Fig S22. ESI-MS^2 spectrum of compound 3 at m/z 1055.6 $[M+H]^+$ 27
Fig S23. ^1H NMR spectrum of compound 3 in DMSO-d_6 (600 MHz) 28
Fig S24. ^{13}C NMR spectrum of compound 3 in DMSO-d_6 (600 MHz) 29
Fig S25. DEPT-135 spectrum of compound 3 in DMSO-d_6 (600 MHz) 30
Fig S26. ^1H-^1H COSY spectrum of compound 3 in DMSO-d_6 (600 MHz) 31
Fig S27. HSQC spectrum of compound 3 in DMSO-d_6 (600 MHz) 32
Fig S28. HMBC spectrum of compound 3 in DMSO-d_6 (600 MHz) 33
Fig S29. TOCSY spectrum of compound 3 in DMSO-d_6 (600 MHz) 34
Fig S30. NOESY spectrum of compound 3 in DMSO-d_6 (600 MHz) 35
Fig S31. Marfey's of compound 3 36
Fig S32. CD spectrum of compound 3 in MeOH 37
Fig S33. ESI-MS^2 spectrum of 4 at m/z 1069.6 $[M+H]^+$ 38
Fig S34. ^1H NMR spectrum of compound 4 in DMSO-d_6 (600 MHz) 39
Fig S35. ^{13}C NMR spectrum of compound 4 in DMSO-d_6 (600 MHz) 40
Fig S36. DEPT-135 spectrum of compound 4 in DMSO-d_6 (600 MHz) 41
Fig S37. ^1H-^1H COSY spectrum of compound 4 in DMSO-d_6 (600 MHz) 42
Fig S38. HSQC spectrum of compound 4 in DMSO-d_6 (600 MHz) 43
Fig S39. HMBC spectrum of compound 4 in DMSO-d_6 (600 MHz) 44
Fig S40. TOCSY spectrum of compound 4 in DMSO-d_6 (600 MHz) 45
Fig S41. NOESY spectrum of compound 4 in DMSO-d_6 (600 MHz) 46
Fig S42. Marfey's of compound 4 47
Fig S43. CD spectrum of compound 4 in MeOH 48
Fig S44. ESI-MS^2 spectrum of 5 at m/z 1083.6 $[M+H]^+$ 49
Fig S45. ^1H NMR spectrum of compound 5 in DMSO-d_6 (600 MHz) 50
Fig S46. ^{13}C NMR spectrum of compound 5 in DMSO-d_6 (600 MHz) 51
Fig S47. DEPT-135 spectrum of compound 5 in DMSO-d_6 (600 MHz) 52
Fig S48. ^1H-^1H COSY spectrum of compound 5 in DMSO-d_6 (600 MHz) 53
Fig S49. HSQC spectrum of compound 5 in DMSO-d_6 (600 MHz) 54
Fig S50. HMBC spectrum of compound 5 in DMSO-d_6 (600 MHz) 55
Fig S51. TOCSY spectrum of compound 5 in DMSO-d_6 (600 MHz) 56
Fig S52. NOESY spectrum of compound 5 in DMSO-d_6 (600 MHz) 57
Fig S53. Marfey's of compound 5 58
Fig S54. CD spectrum of compound 5 in MeOH 59
Fig S55. Protein Molecular Weight Marker used in western blotting system to detect Erk1/2 (42 kda). 60
Fig S56. Original protein image of Erk1/2 (42 kda) detected using the Amersham imager 680 QC 60
Fig S57. Original protein image of *p*-Erk1/2 (42 kda) in DLD-1 cells detected using the Amersham imager 680 QC. 61
Fig S58. Original protein image of p-Erk1/2 (42 kda) in HT-29 cells detected using the Amersham imager 680 QC 61
Fig S59. Original protein image of GAPDH (37 kda) detected using the Amersham imager 680 QC 62

Table S1. ^{1}H (800MHz) and ^{13}C (201MHz) NMR data of compound 3 in DMSO-d_6.

Pos.	δ_C, Type	δ_H, (J in Hz)	Pos.	δ_C, Type	δ_H, (J in Hz)	Pos.	δ_C, Type	δ_H, (J in Hz)
Ac-^{1}Aib			^{5}Iva			^{9}Aib		
$COCH_3$	170.9, C		1	174.1, C		1	174.2, C	
$COCH_3$	23.0, CH_3	1.91, s	2	58.6, C		2	55.8[a], C	
1	175.6, C		3a	26, CH_2	2.17, m	3	22.9[b], CH_3	1.31[g], m
2	55.9[a], C		3b		1.62, m	4	25.7[c], CH_3	1.42[g], m
3	23.1[b], CH_3	1.36[g], m	4	7.2, CH_3	0.70, t, (7.5)	NH		7.50, m
4	25.7[c], CH_3	1.44[g], m	5	22.6[b], CH_3	1.31[g], m			
NH		8.59, br s	NH		7.49, m			
^{2}Ser			^{6}Gln			^{10}Gly		
1	171.2, C		1	173.4[f], C		1	168.6, C	
2	57.5, C		2	55.7, CH	3.77, m	2a	42.6, CH_2	3.56, s
3a	60.6, CH_2	3.75, m	3	26.0, CH_2	1.98, m	2b		
3b		3.67, m	4a	31.5, CH_2	2.17, m	NH		7.82, m
OH			4b		2.26, m			
NH		8.08, br s	5	173.2, C	6.75, br s			
			NH_2a		7.17, s			
			NH_2b		6.75, s			
			NH		7.80, m			
^{3}Ala			^{7}Aib			^{11}Leu		
1	174.5[d], C		1	174.6[d], C		1	171.3, C	
2	50.6[e], CH	4.05, m	2	56.1, C		2	50.6[e], CH	4.08, m
3	16.2, CH_3	1.31[g], m	3	23.2, CH_3	1.36[g], m	3a	40, CH_2	1.47, m
NH		7.87, s	4	26.1, CH_3	1.42[g], m	3b		1.62, m
			NH		7.81, m	4	24.0[c], CH	1.68, m
						5	21.4, CH_3	0.82, d, (6.6)
						6	23, CH_3	0.87, d, (6.4)
						NH		7.48, m
^{4}Aib			^{8}Val					
1	174.0, C		1	176.9[f], C				
2	55.8[a], C		2	57.8, CH	4.13, m			
3	23.1[b], CH_3	1.34[g], m	3	29, CH	2.28, m			
4	24.1[c], CH_3	1.45[g], m	4	19.4, CH_3	0.80, m			
NH		7.50, m	5	17.6, CH_3	0.84, d, (6.7)			
			NH		6.96, d, (9.4)			

[a–g] Assignments for overlapping ^{1}H and ^{13}C NMR resonances with the same superscript may be interchanged.

Table S2. ^{1}H **(800MHz) and** ^{13}C **(201MHz) NMR data of compound 4 in DMSO-d_6.**

Pos.	δ_C, Type	δ_H, (J in Hz)	Pos.	δ_C, Type	δ_H, (J in Hz)	Pos.	δ_C, Type	δ_H, (J in Hz)
Ac-^{1}Aib			^{5}Iva			^{9}Aib		
$COCH_3$	170.8, C		1	174.1, C		1	174.2, C	
$COCH_3$	23.0, CH_3	1.91, s	2	58.7[f], C		2	56.1[a], C	
1	175.5, C		3a	26.0[g], CH_2	1.95, m	3	22.4[b], CH_3	1.35[i], m
2	55.8[a], C		3b		2.22, m	4	25.6[c], CH_3	1.41[i], m
3	24.0[b], CH_3	1.30[i], m	4	7.1[h], CH_3	0.73, m	NH		7.58, br s
4	26.1[c], CH_3	1.40[i], m	5	23.0[b], CH_3	1.34[i], m			
NH		8.56, br s	NH		7.52, m			
^{2}Ser			^{6}Gln			^{10}Gly		
1	171.5[d], C		1	173.4, C		1	168.8, C	
2	57.8, CH	4.06, m	2	55.9[a], CH	3.77, m	2a	42.6, CH_2	3.56, m
3a	60.6, CH_2	3.75, m	3	26.3[g], CH_3	1.98, m	2b		
3b		3.68, m	4a	31.4, CH_2	2.19, m	NH		7.80, m
OH			4b		2.29, m			
NH		8.04, br s	5	173.2, C				
			NH_2a		7.17, d, (16.1)			
			NH_2b		6.75, s			
			NH		7.87, m			
^{3}Ala			^{7}Aib			^{11}Leu		
1	175.0, C		1	174.5, C		1	171.2, C	
2	50.5[e], CH	4.06, m	2	56.2[a], C		2	50.8[e], CH	4.09, m
3	16.2, CH_3	1.31[i], m	3	22.8[b], CH_3	1.34[i], m	3a	40.1, CH_2	1.46, m
NH		7.92, m	4	25.5[c], CH_3	1.40[i], m	3b		1.63, m
			NH		7.77, m	4	24.2, CH	1.69, m
						5	23.1, CH_3	0.85, m
						6	21.4, CH_3	0.78, m
						NH		7.50, m
^{4}Iva			^{8}Val					
1	172.6, C		1	177.0, C				
2	58.6[f], C		2	57.5, CH	4.15, m			
3a	26.0[g], CH_2	1.94, m	3	28.6, CH_2	2.32, m			
3b		2.20, m	4	17.6, CH_3	0.87, d, (6.4)			
4	7.4[h]	0.72, m	5	19.4, CH_3	0.80, m			
5	22.6[b], CH_3	1.33[i], m	NH		6.95, d, (8.8)			
NH		7.43, s						

[a–i] Assignments for overlapping ^{1}H and ^{13}C NMR resonances with the same superscript may be interchanged.

Table S3. ^{1}H (600MHz) and ^{13}C (150MHz) NMR data of compound 5 in DMSO-d_6.

Pos.	δ_C, Type	δ_H, (*J* in Hz)	Pos.	δ_C, Type	δ_H, (*J* in Hz)	Pos.	δ_C, Type	δ_H, (*J* in Hz)
Ac-^{1}Aib			^{5}Iva			^{9}Aib		
$COCH_3$	170.9, C		1	175.1, C		1	174.0, C	
$COCH_3$	22.7, CH_3	1.90, s	2	58.8[d], C		2	56.1[a], C	
1	175.6, C		3a	25.9[e], CH_2	1.64, m	3	23.1[b], CH_3	1.31, m
2	55.8[a], C		3b		2.19, m	4	26[c], CH_3	1.36, m
3	23.0[b], CH_3	1.31[g], m	4	7.3[f], CH_3	0.70, m	NH		7.59, br s
4	25.6[c], CH_3	1.37[g], m	5	23.0[b], CH_3	1.34[g], m			
NH		8.62, br s	NH		7.45, s			
^{2}Ser			^{6}Gln			^{10}Gly		
1	171.5, C		1	173.1, C		1	168.6, C	
2	57.5, CH	4.05, m	2	55.7[a], CH	3.80, m	2a	42.6, CH_2	3.57, m
3a	60.6, CH_2	3.74, m	3	26.3, CH_2	1.97, m	2b		
3b		3.64, m	4a	31.6, CH_2	2.22, m	NH		7.75, m
OH			4b		2.34, m			
NH		8.14, br s	5	173.5, C				
			NH_2a		6.74, s			
			NH_2b		7.19, s			
			NH		7.91, s			
^{3}Ala			^{7}Iva			^{11}Leu		
1	175.1, C		1	175.0, C		1	171.2, C	
2	50.8, CH	4.08, m	2	59.0[d], C		2	50.8, CH	4.10, m
3	16.4, CH_3	1.33[g], m	3a	25.8[e], CH_2	1.63, m	3a	40, CH_2	1.62, m
NH		7.92, m	3b		2.14, m	3b		1.48, m
			4	7.2[f], CH_3	0.71, m	4	24.1, CH	1.66, m
			5	22.7[b], CH_3	1.34, m	5	22.1[b], CH_3	0.87, (6.6)
			NH		7.50, s	6	21.5, CH_3	0.82, m
						NH		7.49, d, (7.7)
^{4}Iva			^{8}Val					
1	175.1, C		1	176.9, C				
2	58.7[d], C		2	57.8[d], CH	4.13, m			
3a	25.7[e], CH_2	1.63, m	3	28.7, CH	2.31, m			
3b		2.16, m	4	19.4, CH_3	0.82, m			
4	7.4[f], CH_3	0.74, m	5	17.6, CH_3	0.84, d, (6.6)			
5	22.5[b], CH_3	1.33[g], m	NH		6.98, d, (8.8)			
NH		7.71, s						

[a–g] Assignments for overlapping ^{1}H and ^{13}C NMR resonances with the same superscript may be interchanged.

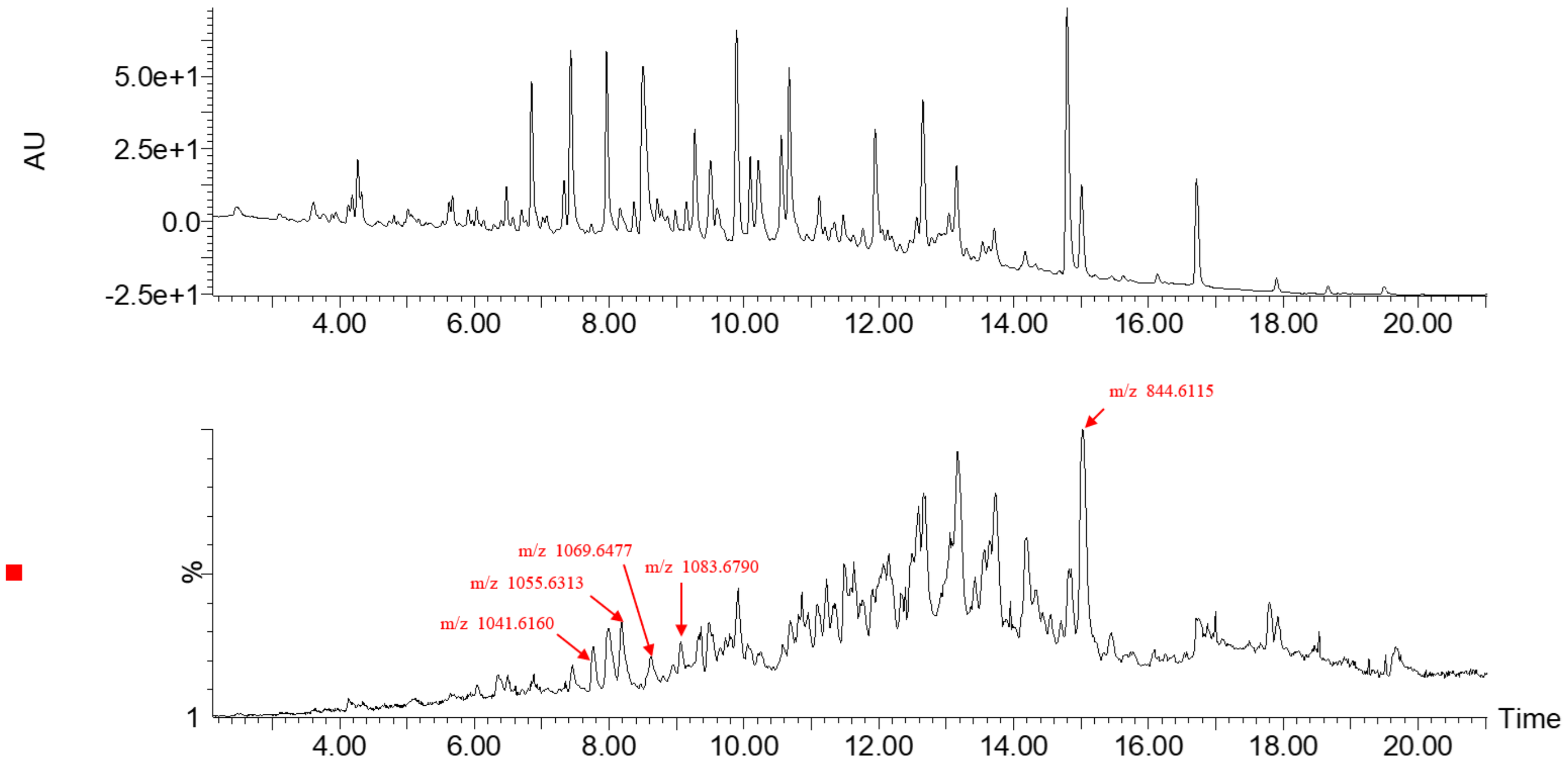

Fig S1. LC-MS and UV spectra of crude extract

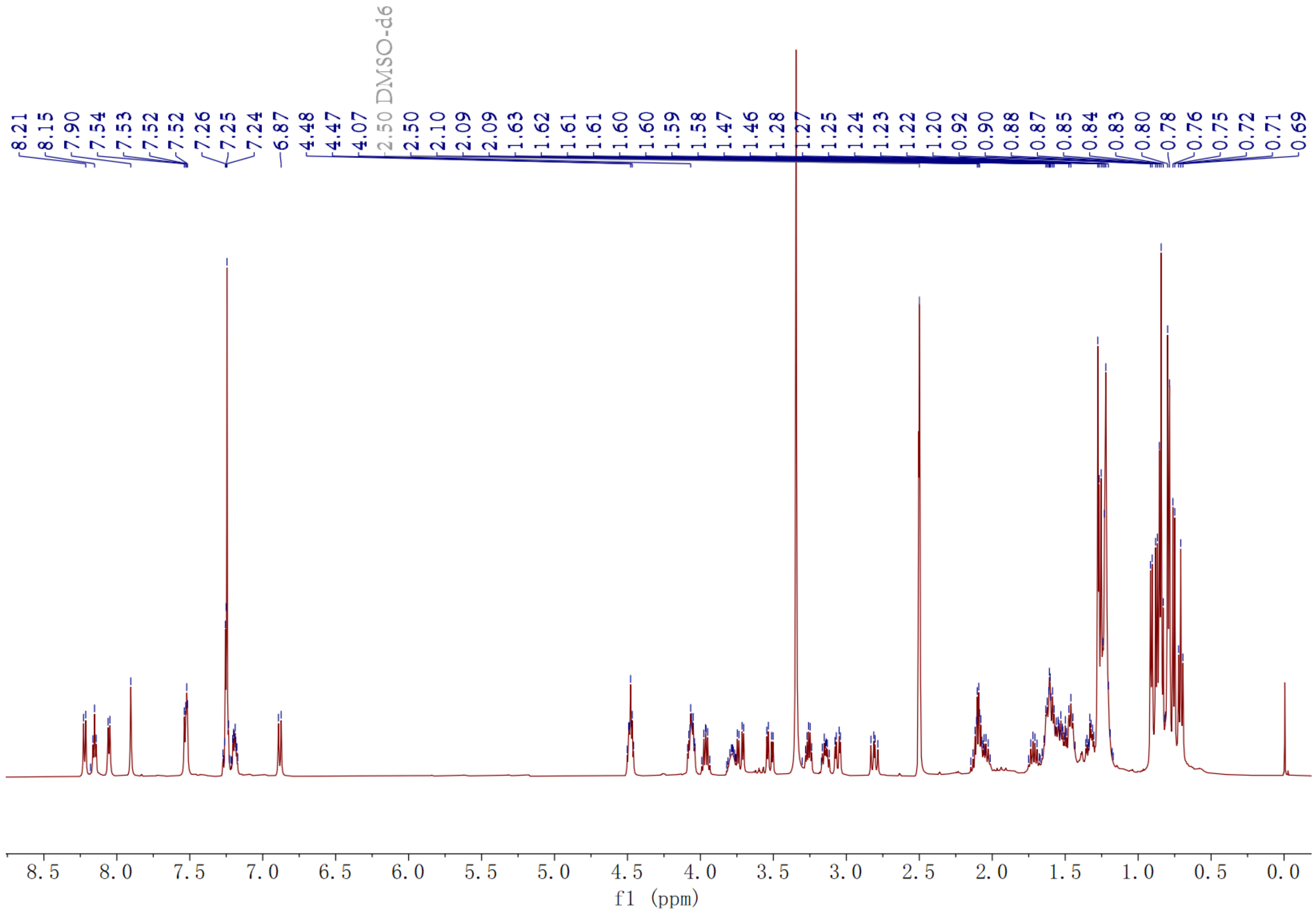

Fig S2. ¹H NMR spectrum of compound 1 in DMSO-*d*₆ (600 MHz)

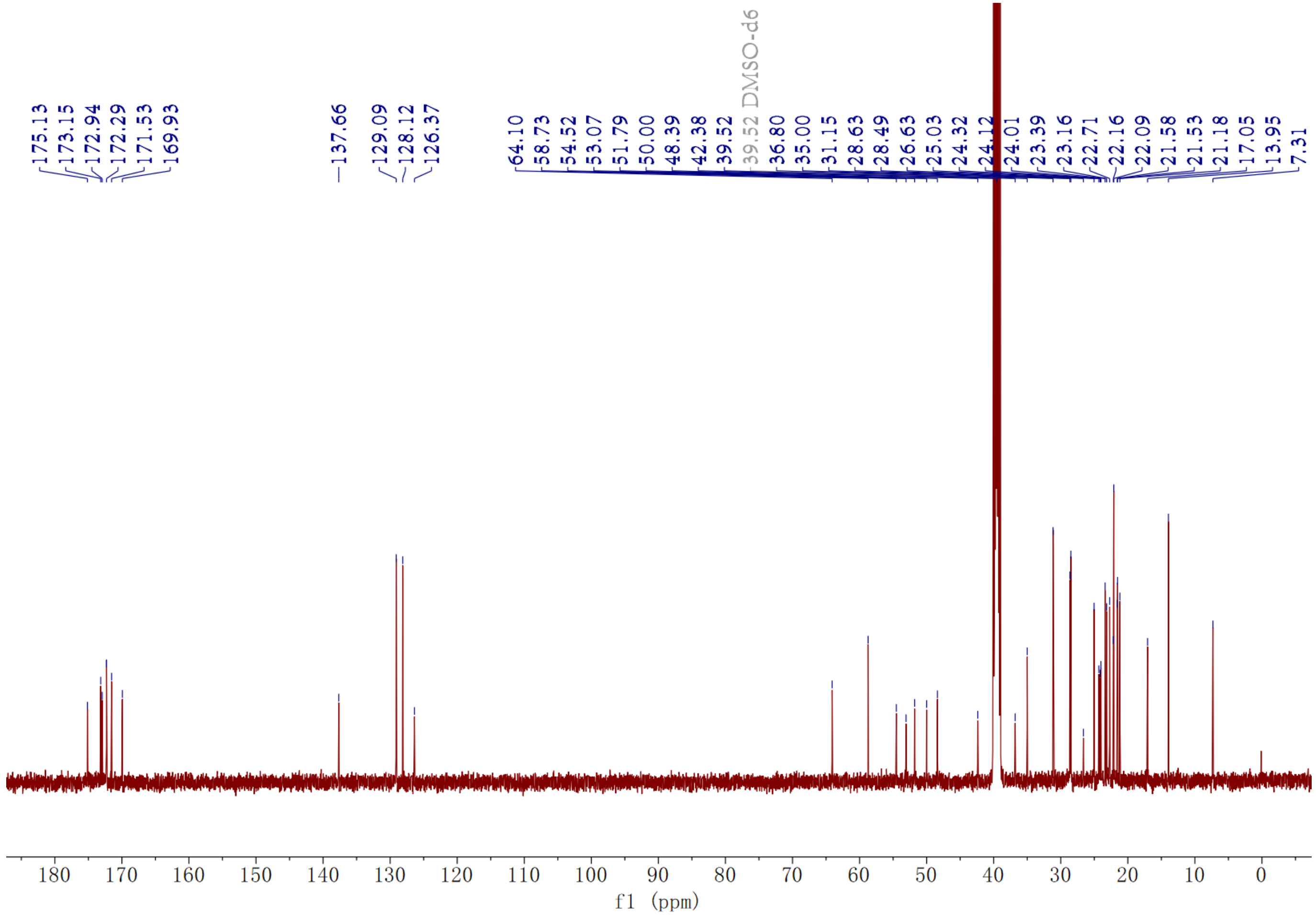

Fig S3. ^{13}**C NMR spectrum of compound 1 in DMSO-*d*$_6$ (600 MHz)**

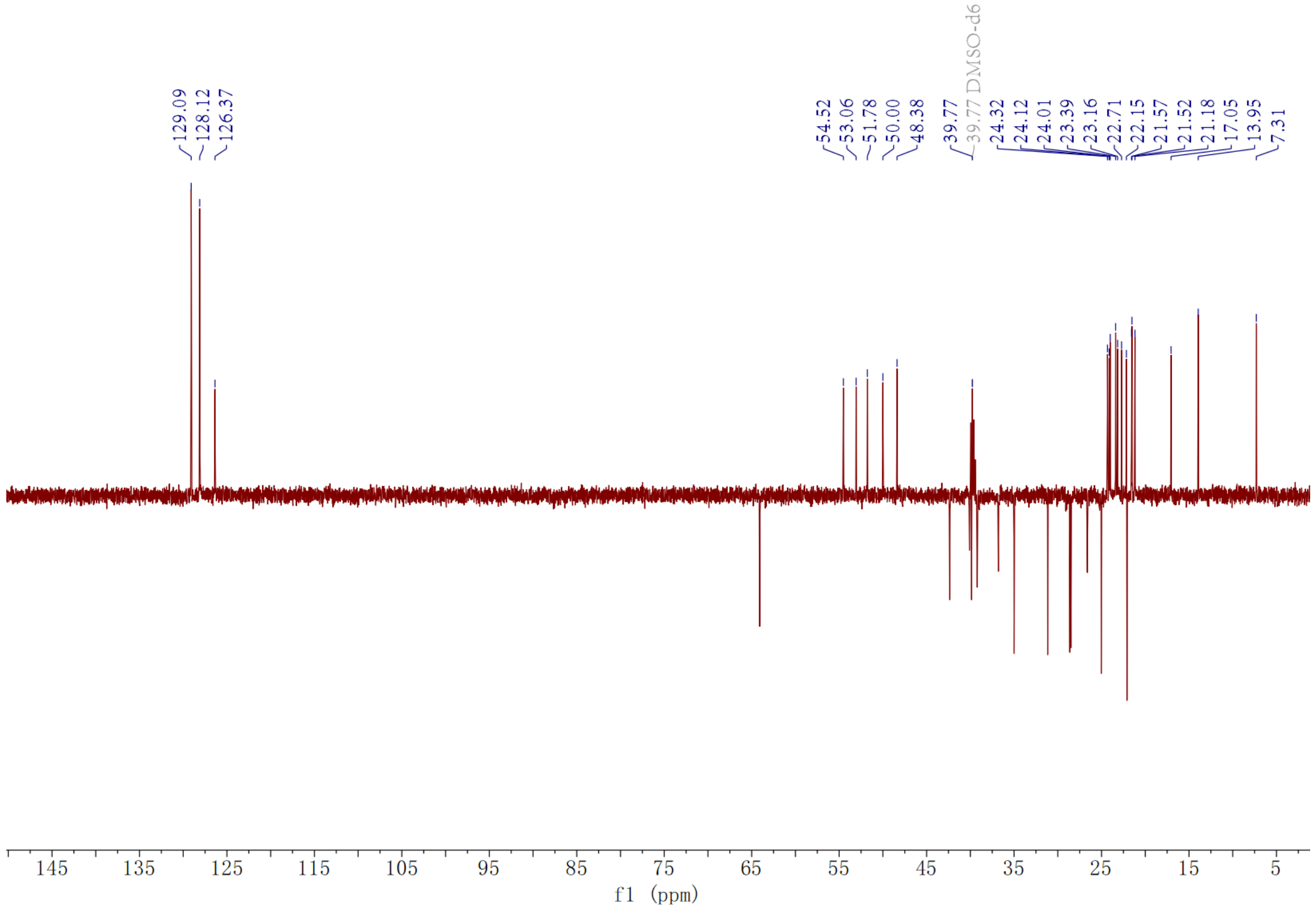

Fig S4. DEPT-135 spectrum of compound 1 in DMSO-d_6 (600 MHz)

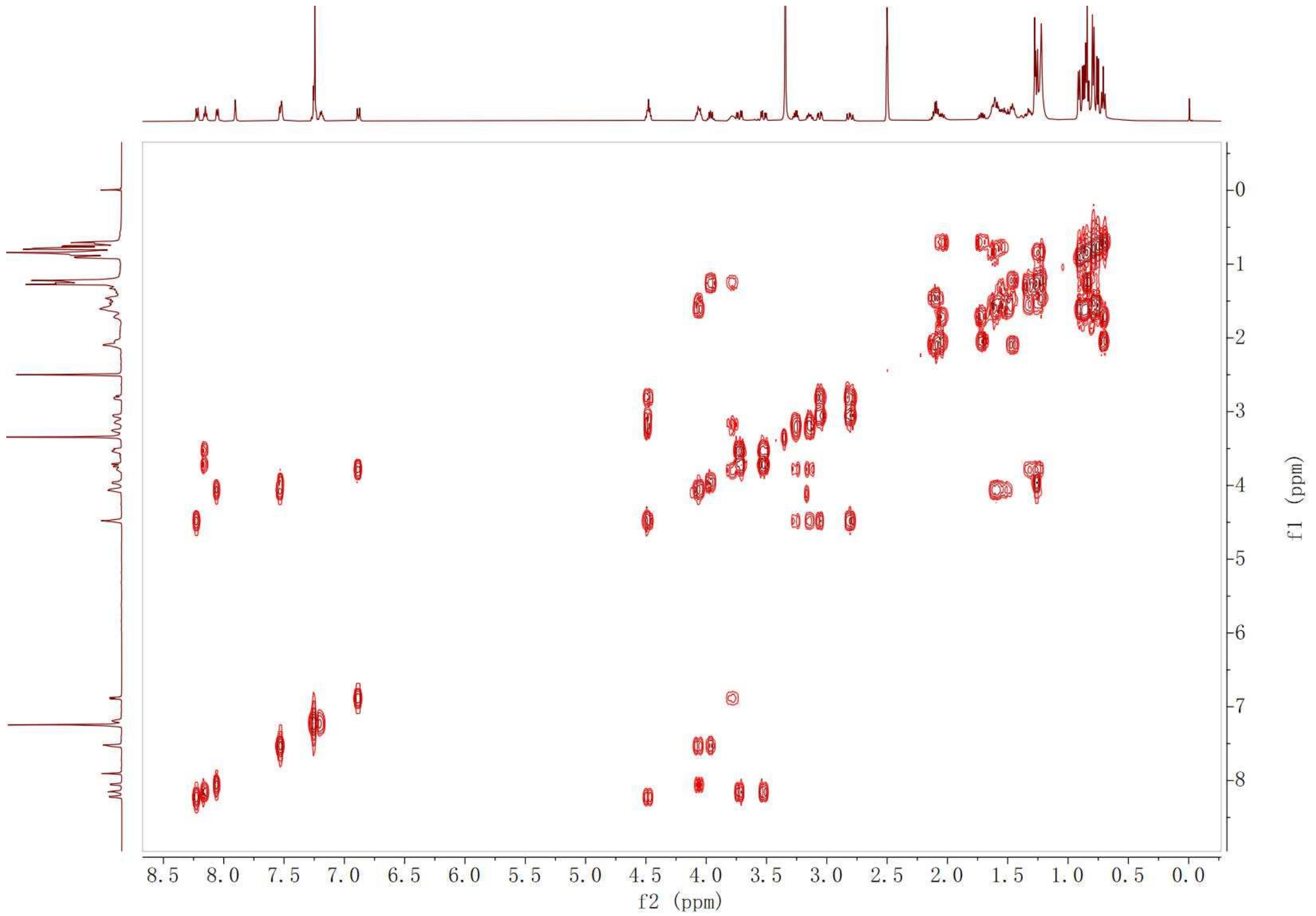

Fig S5. ^{1}H-^{1}H COSY spectrum of compound 1 in DMSO-d_6 (600 MHz)

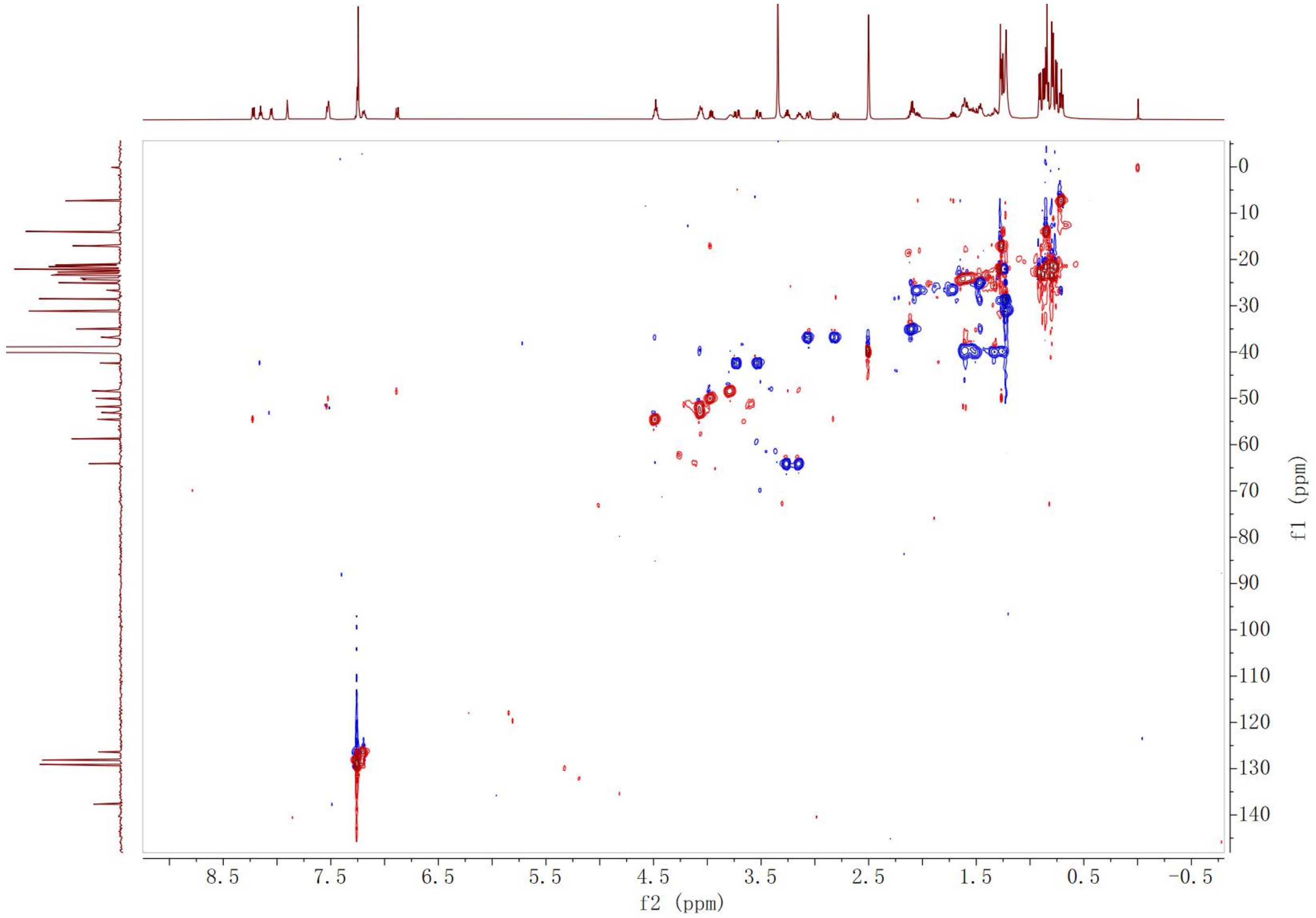

Fig S6. HSQC spectrum of compound 1 in DMSO-d_6 (600 MHz)

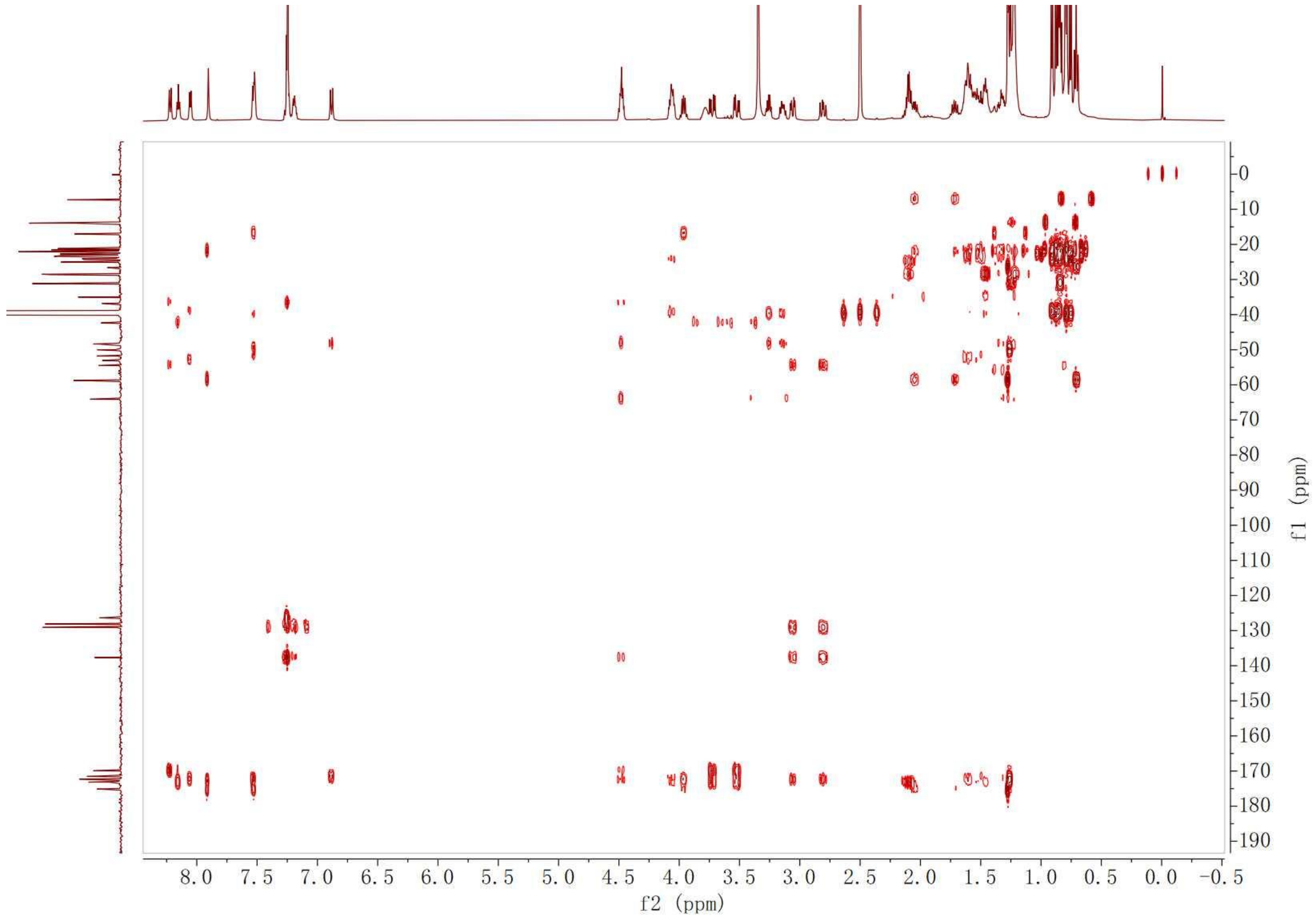

Fig S7. HMBC spectrum of compound 1 in DMSO-d_6 (600 MHz)

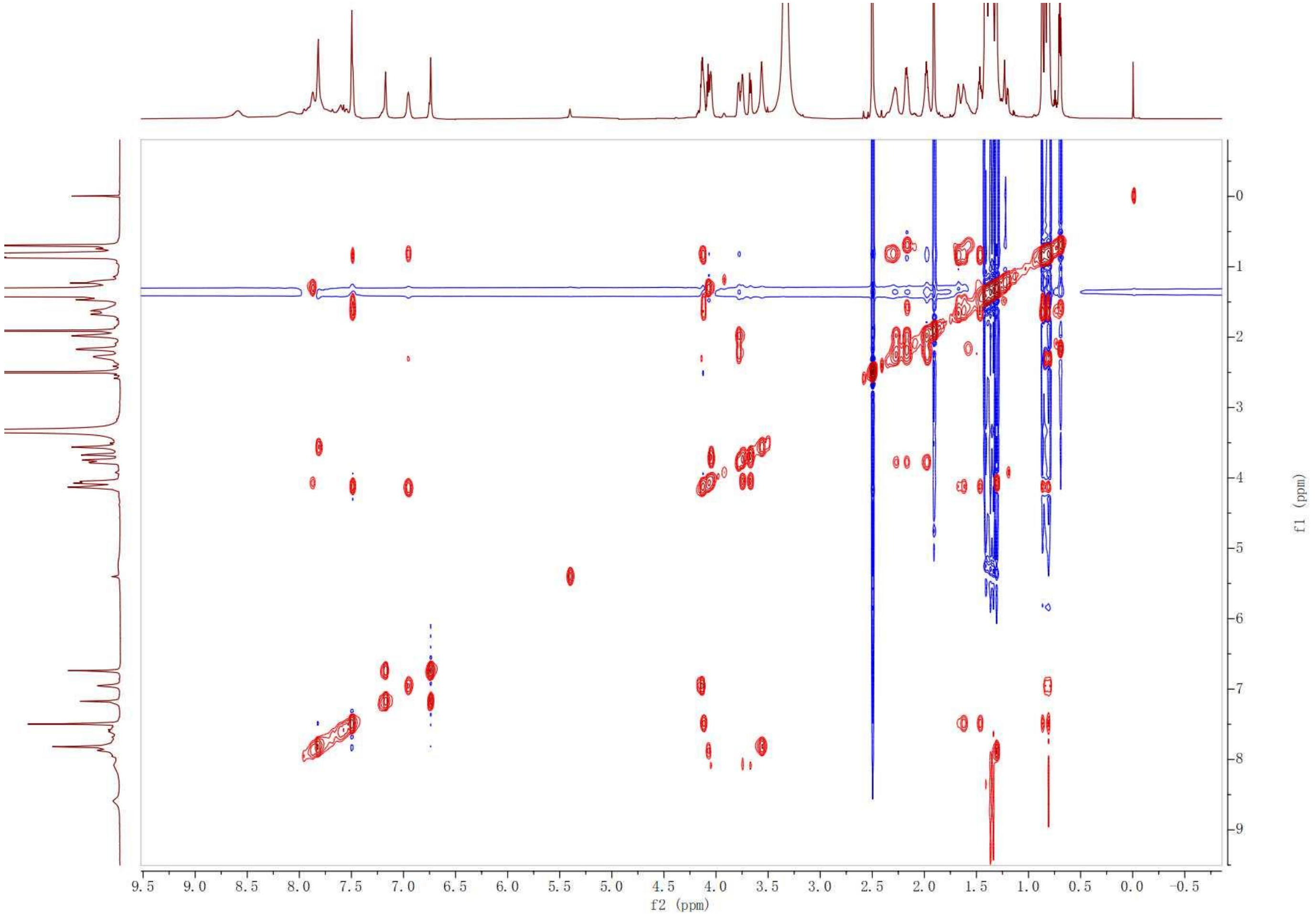

Fig S8. TOCSY spectrum of compound 1 in DMSO-d_6 (600 MHz)

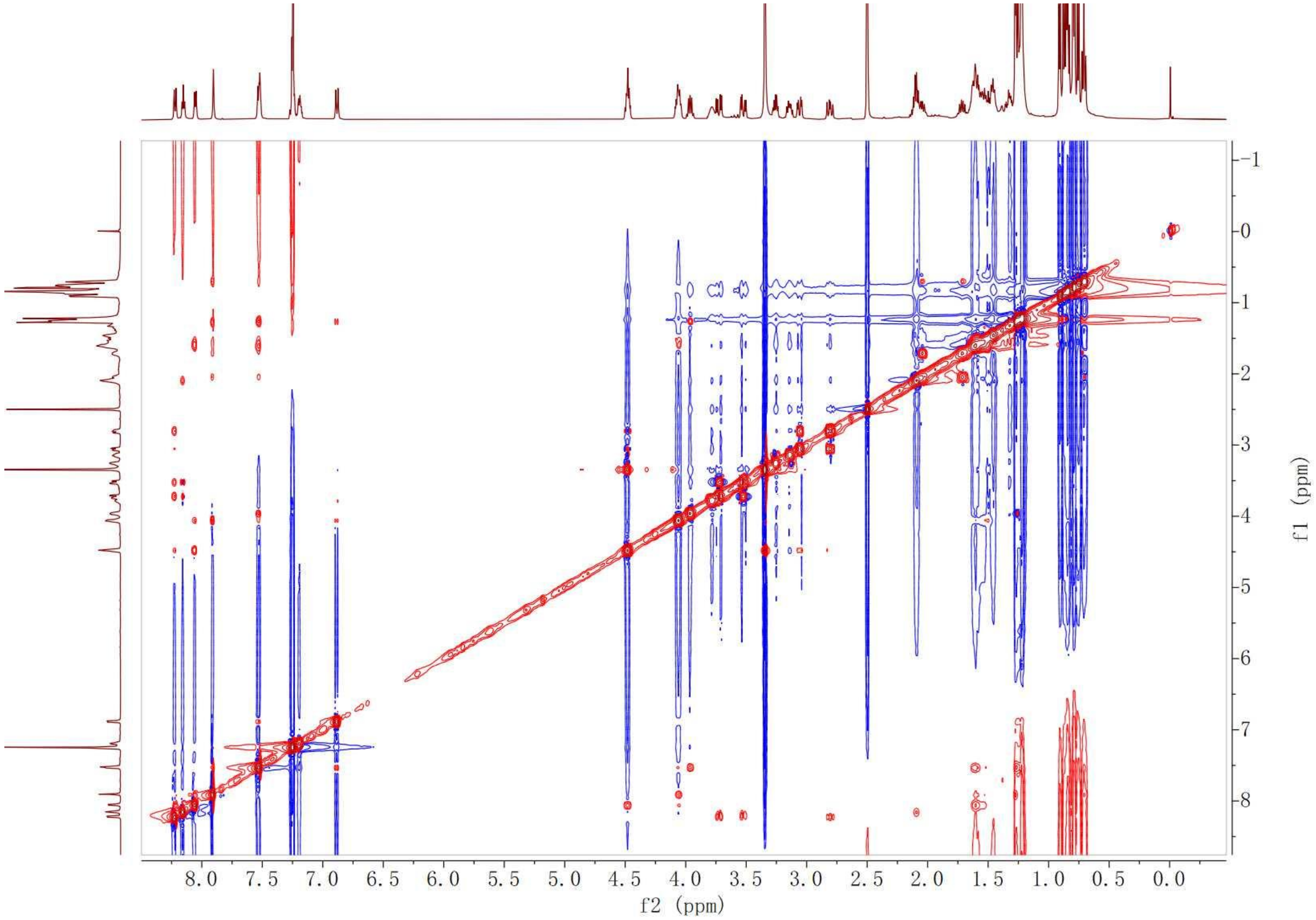

Fig S9. NOESY spectrum of compound 1 in DMSO-*d*$_6$ (600 MHz)

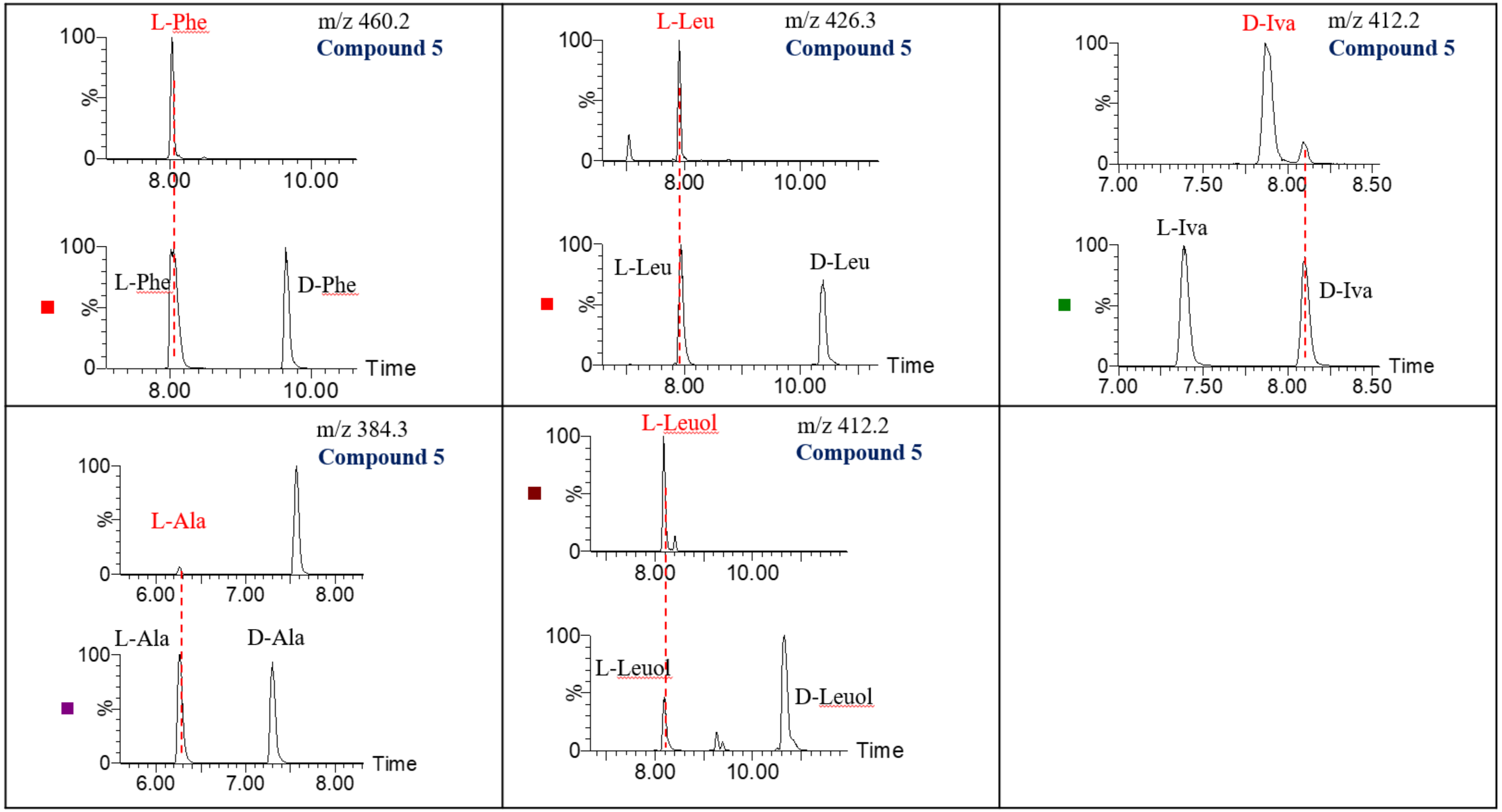

Fig S10. Marfey's of compound 1

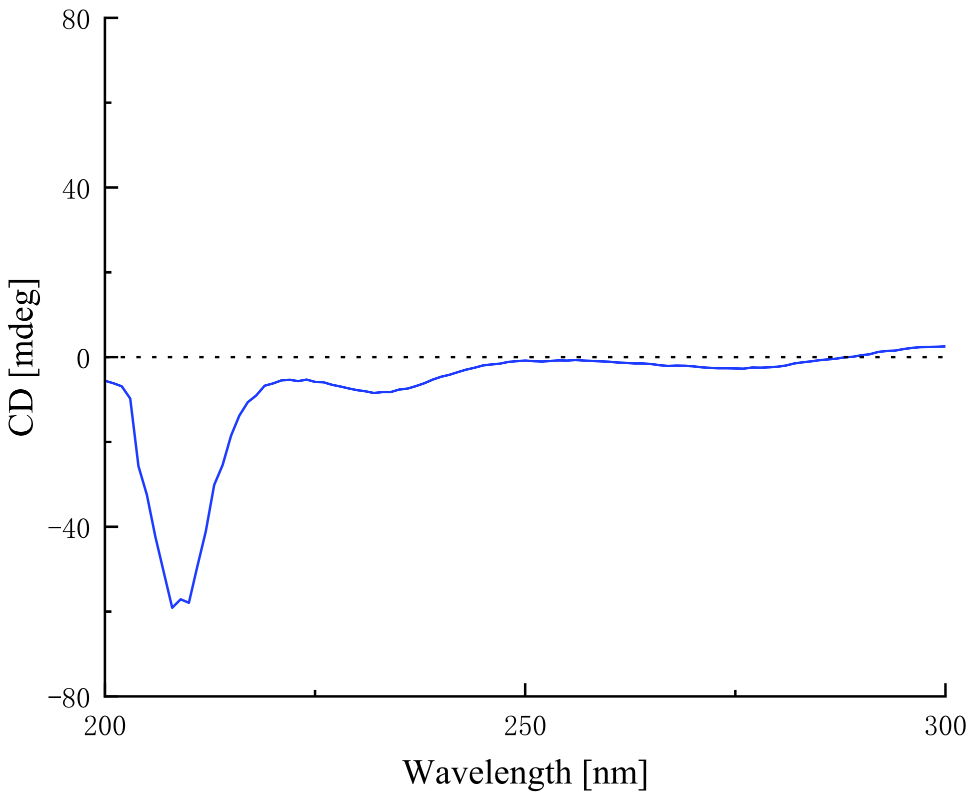

Fig S11. CD spectrum of compound 1 in MeOH

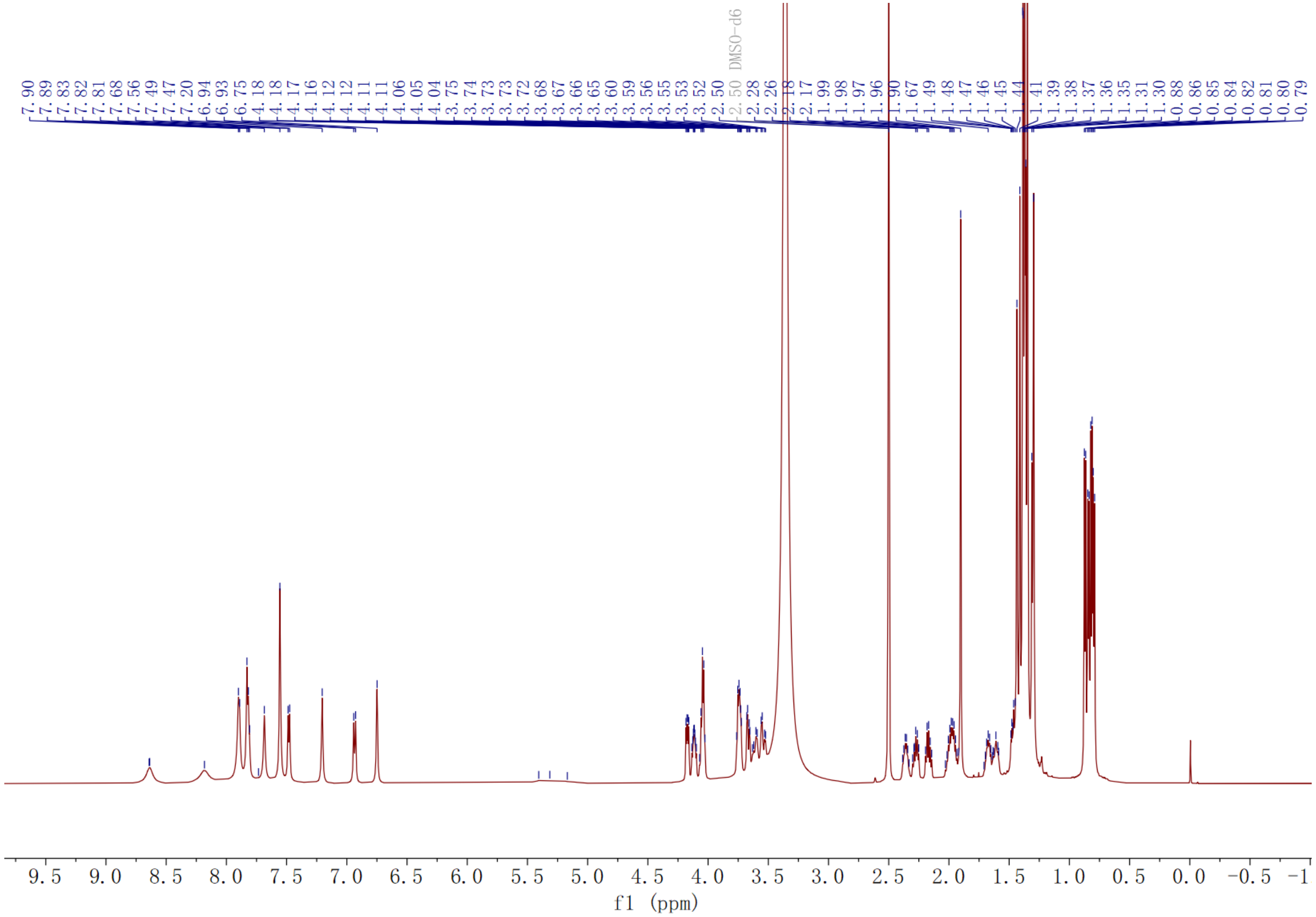

Fig S12. ^{1}H NMR spectrum of compound 2 in DMSO-d_6 (600 MHz)

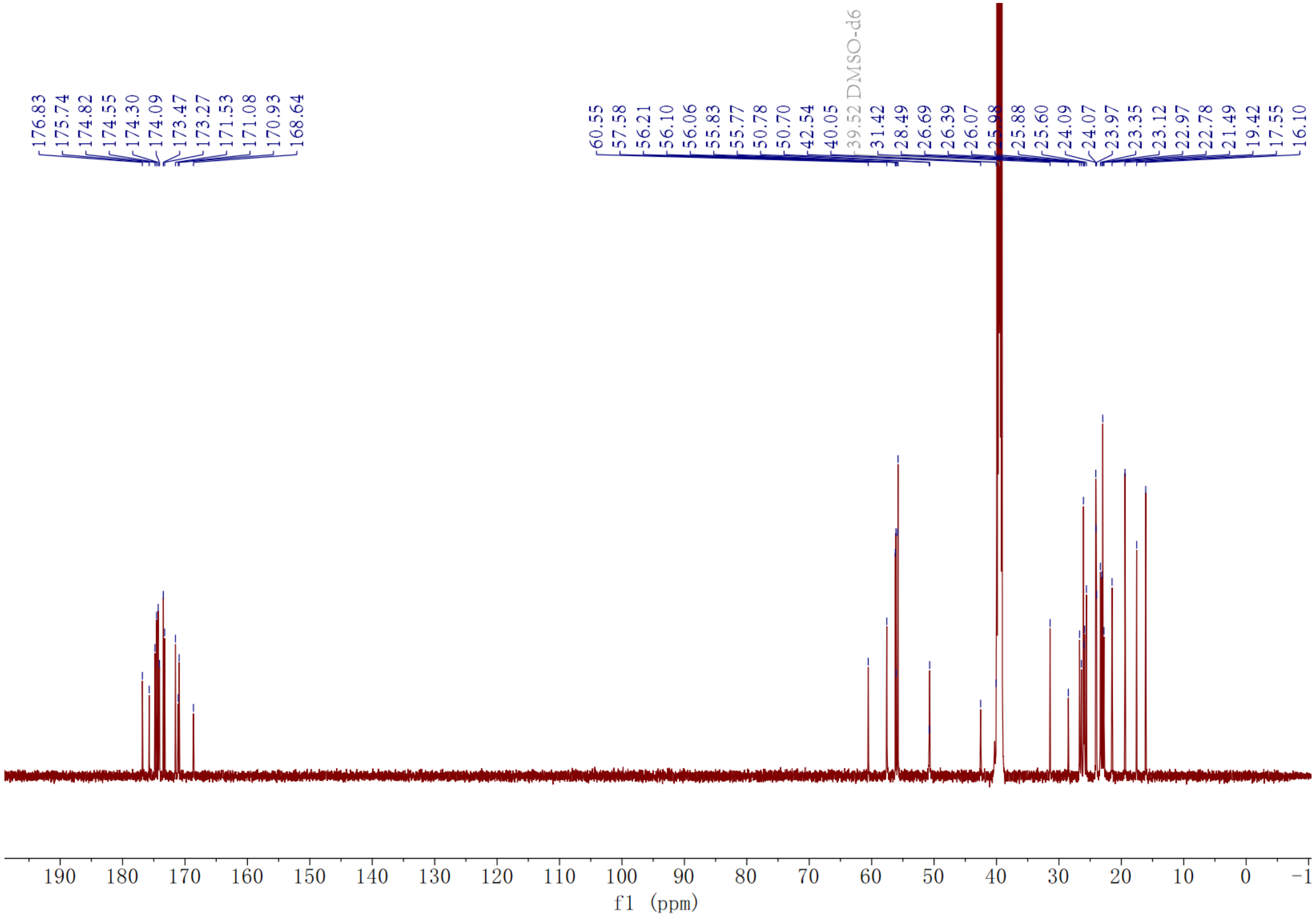

Fig S13. ^{13}C NMR spectrum of compound 2 in DMSO-*d*$_6$ (600 MHz)

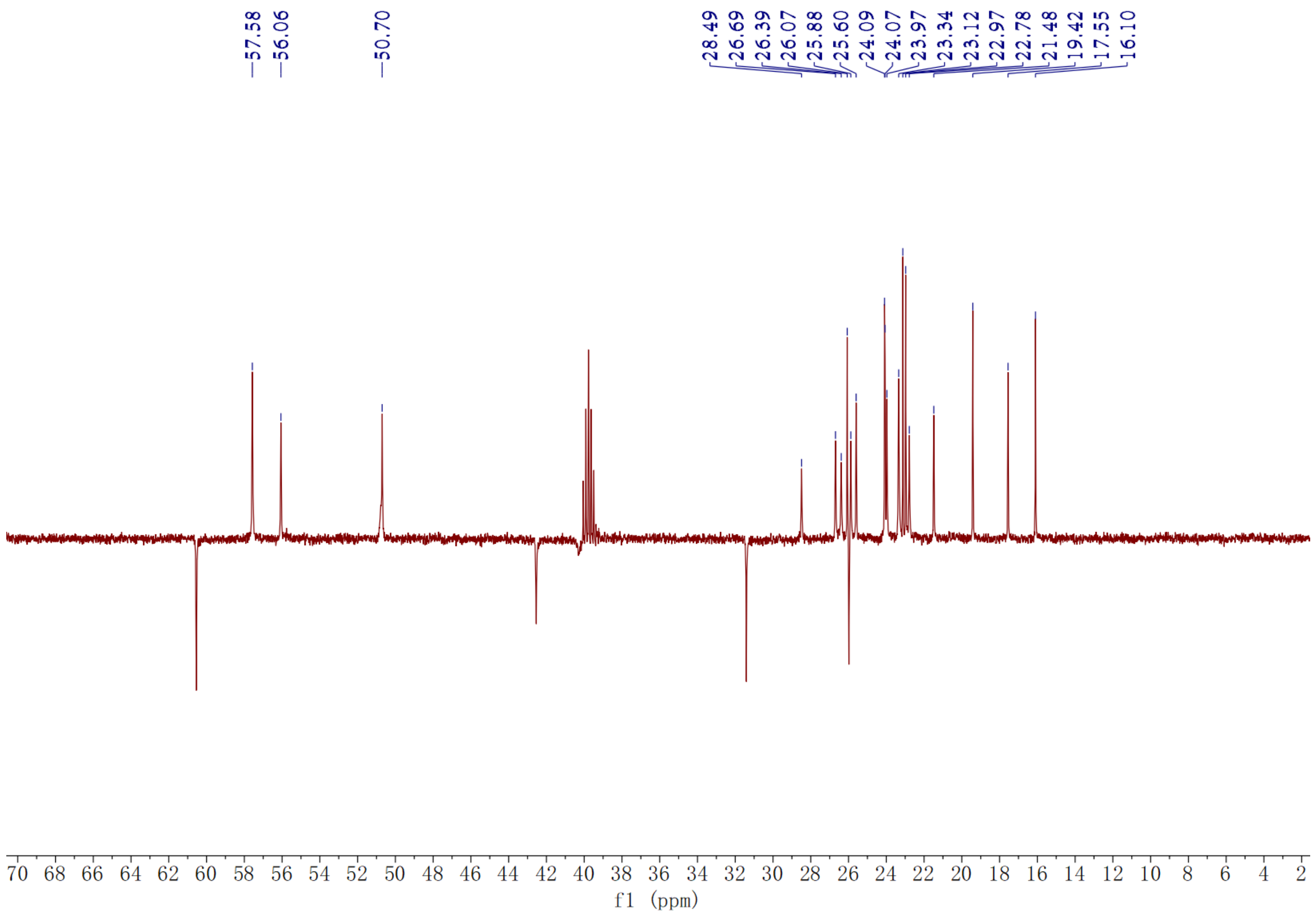

Fig S14. DEPT-135 spectrum of compound 2 in DMSO-*d*$_6$ (600 MHz)

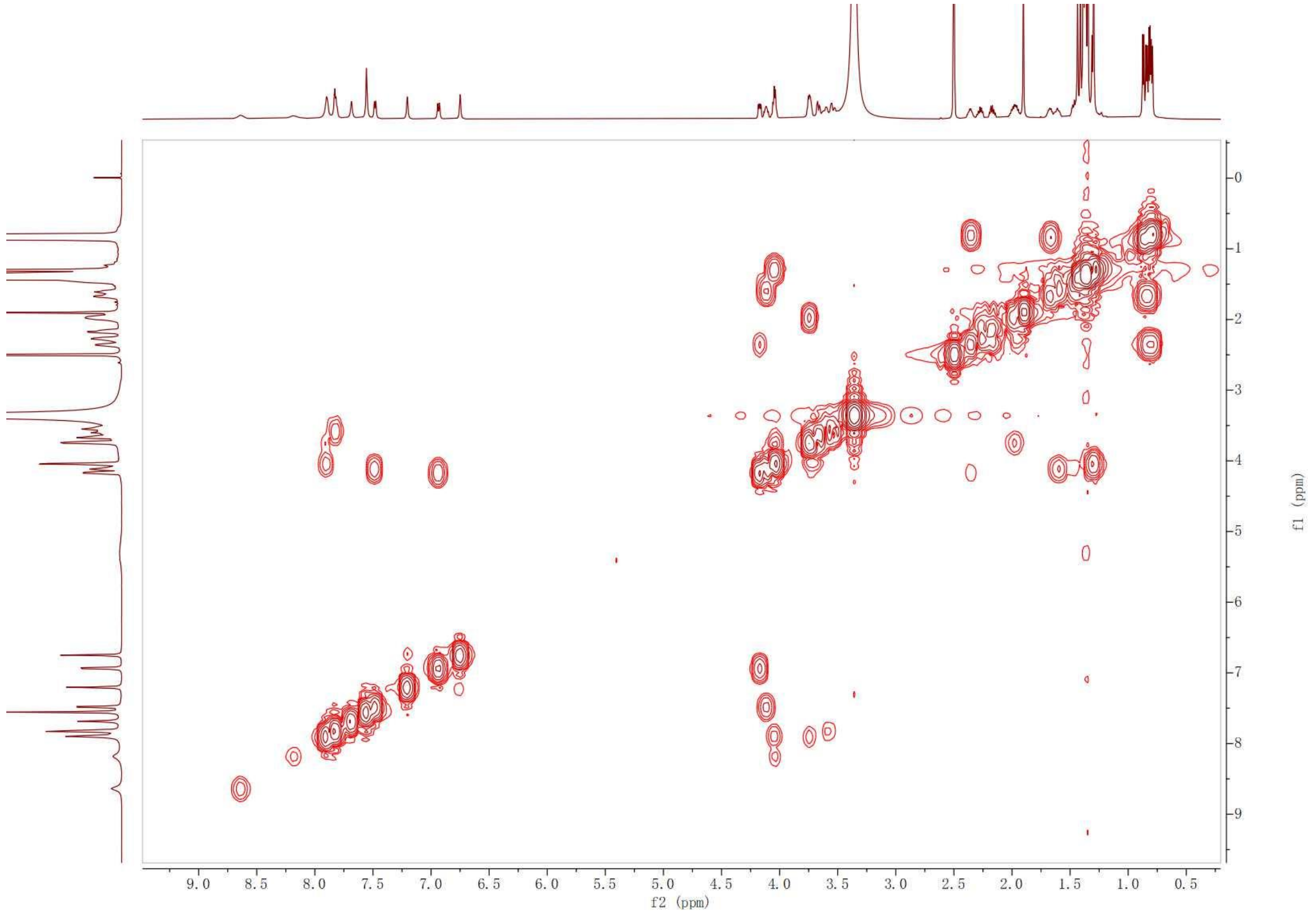

Fig S15. ^{1}H-^{1}H COSY spectrum of compound 2 in DMSO-*d*$_6$ (600 MHz)

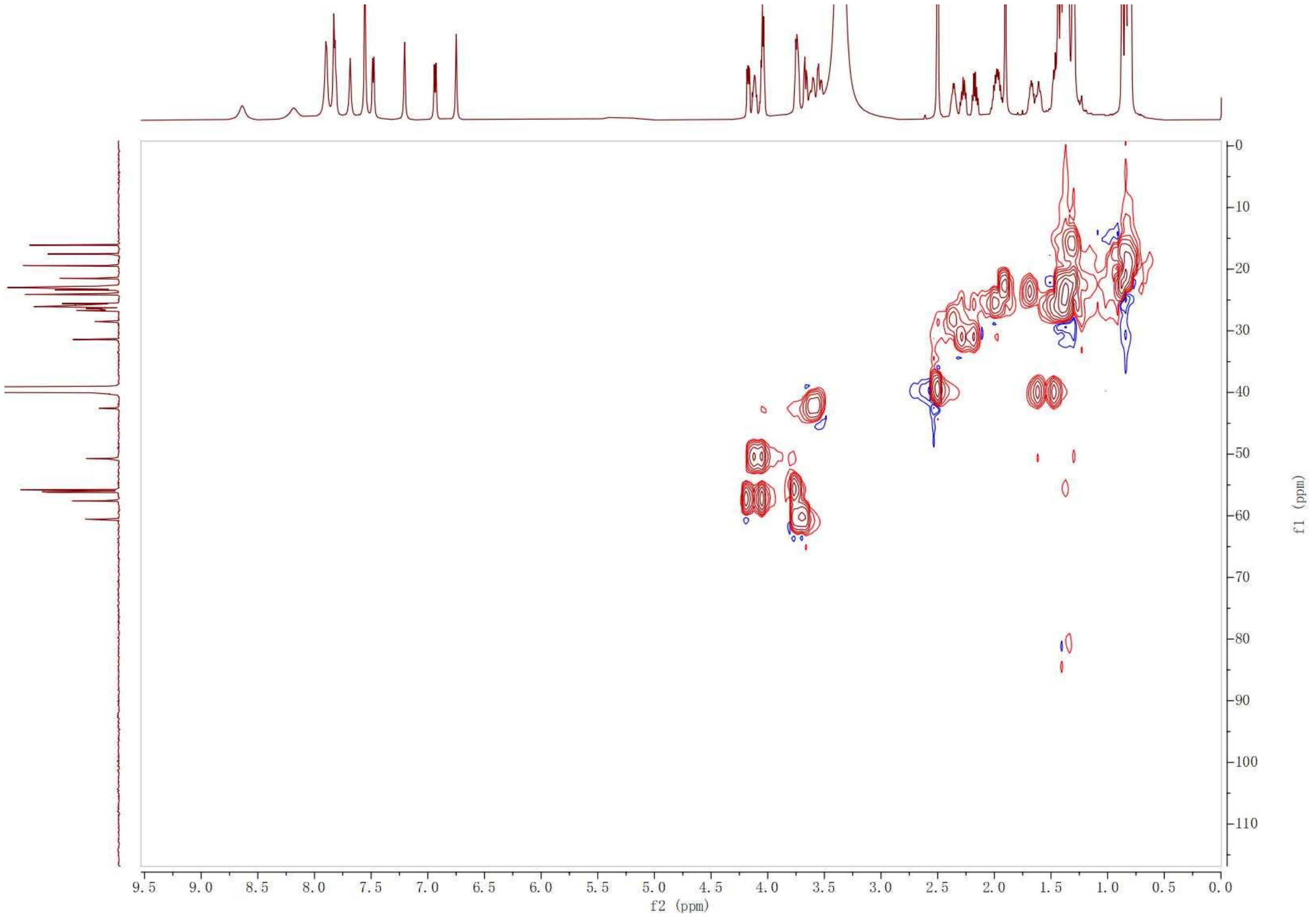

Fig S16. HSQC spectrum of compound 2 in DMSO-*d*₆ (600 MHz)

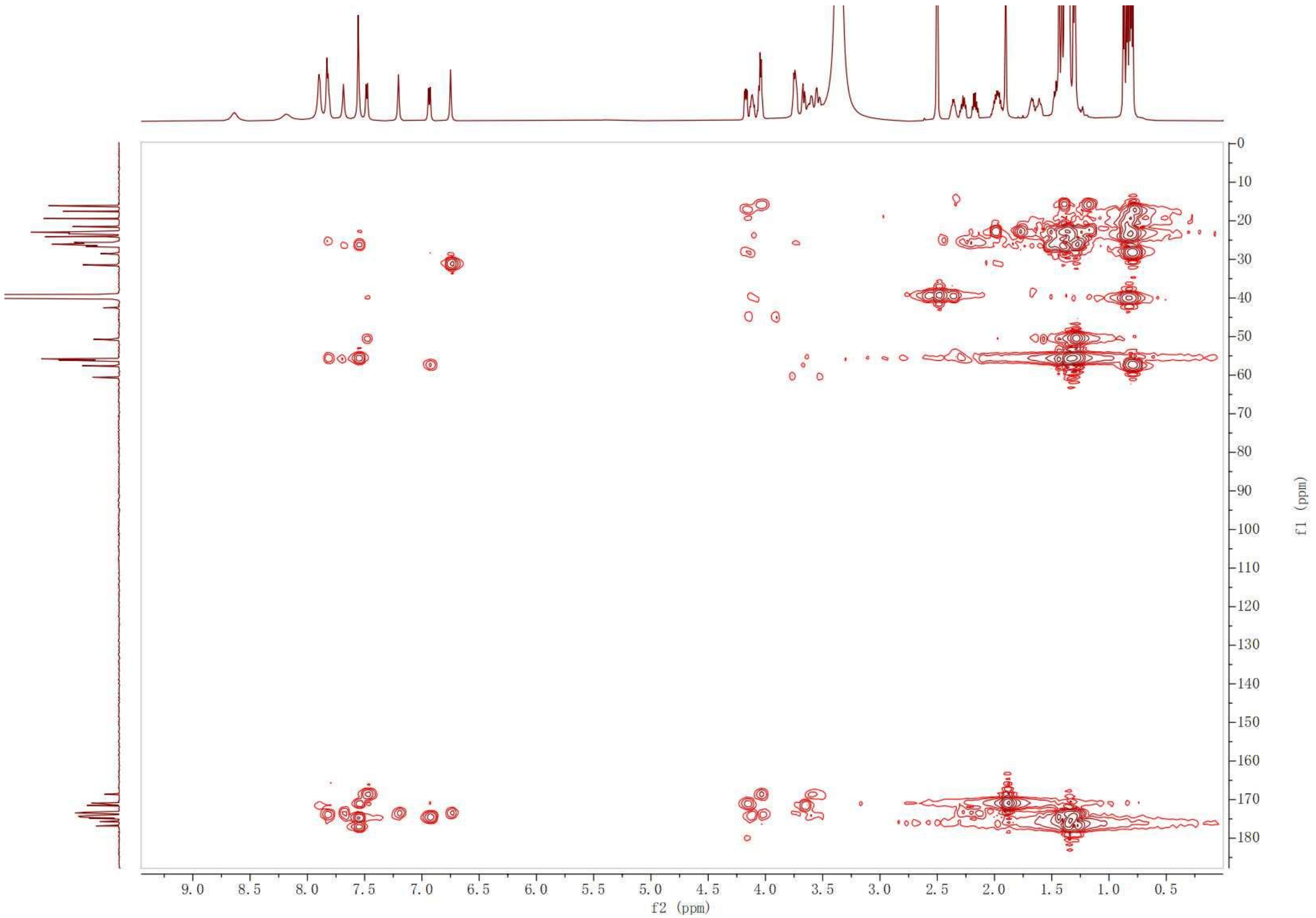

Fig S17. HMBC spectrum of compound 2 in DMSO-d_6 (600 MHz)

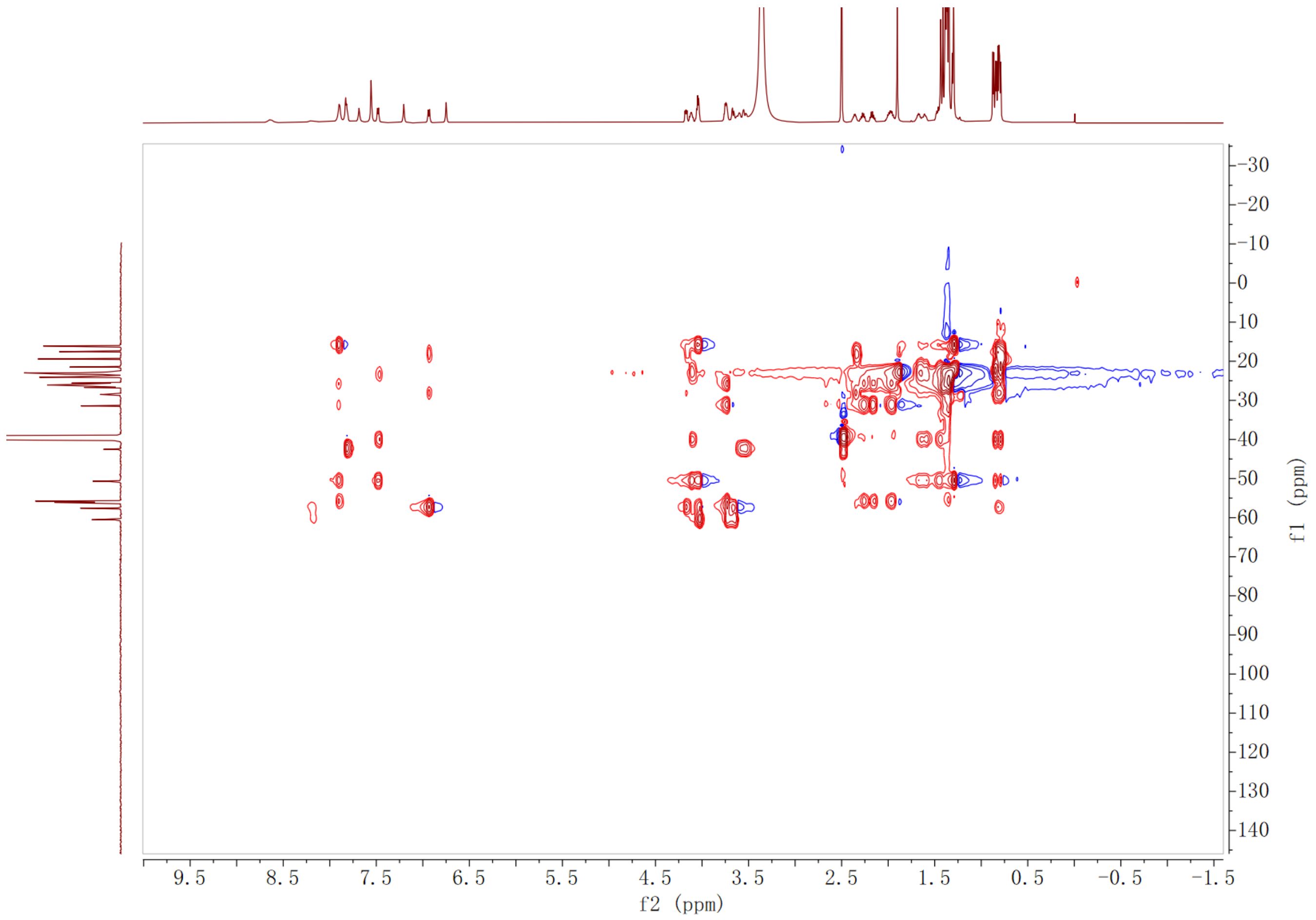

Fig S18. TOCSY spectrum of compound 2 in DMSO-*d*₆ (600 MHz)

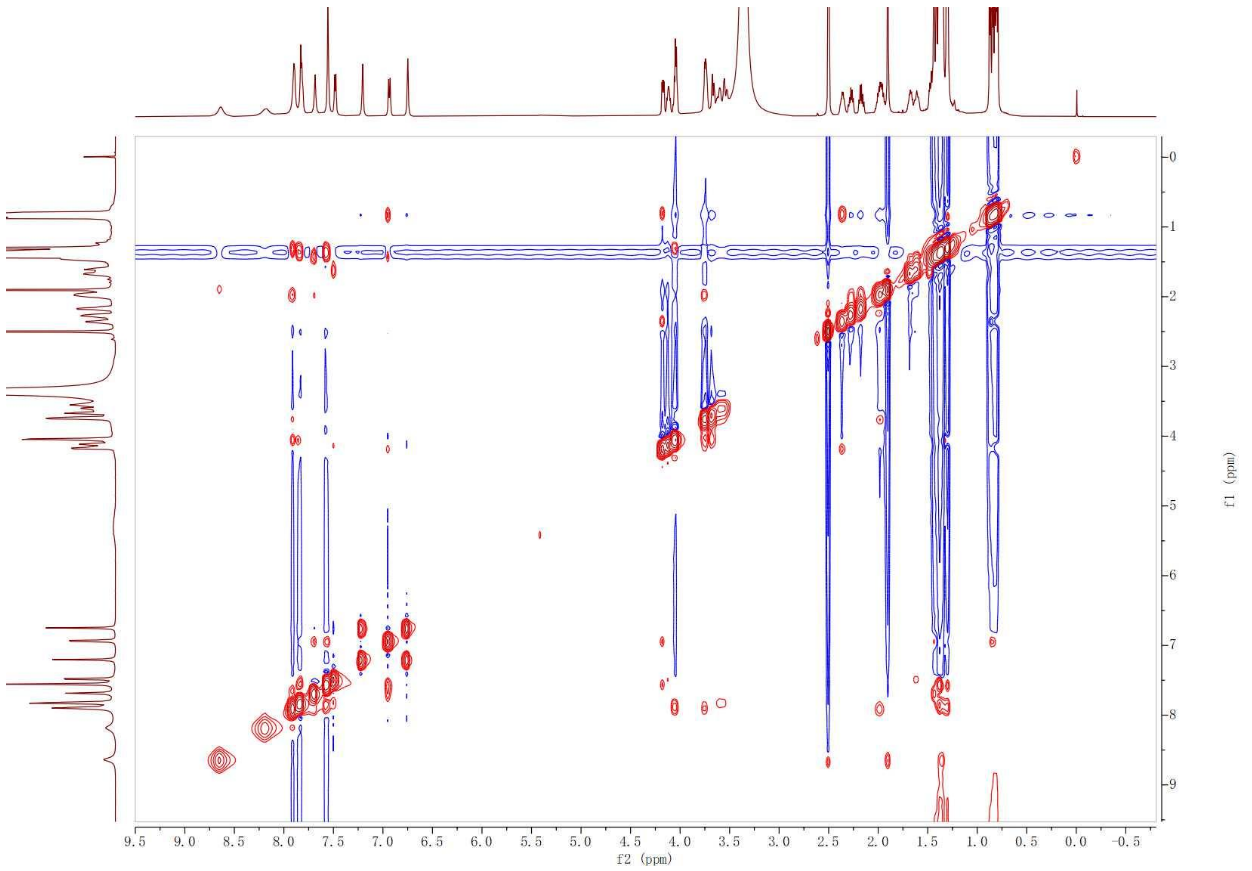

Fig S19. NOESY spectrum of compound 2 in DMSO-*d*₆ (600 MHz)

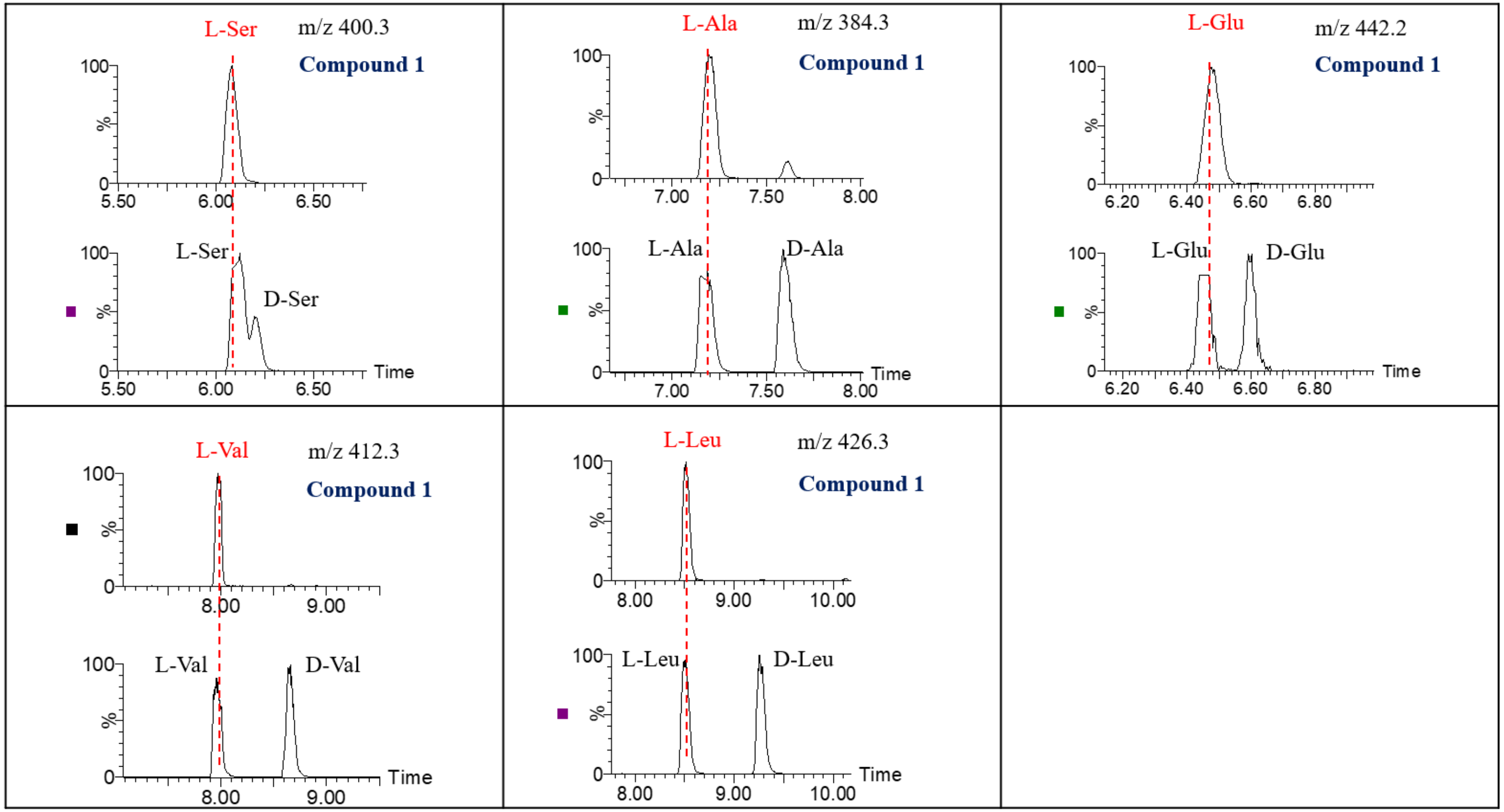

Fig S20. Marfey's of compound 2

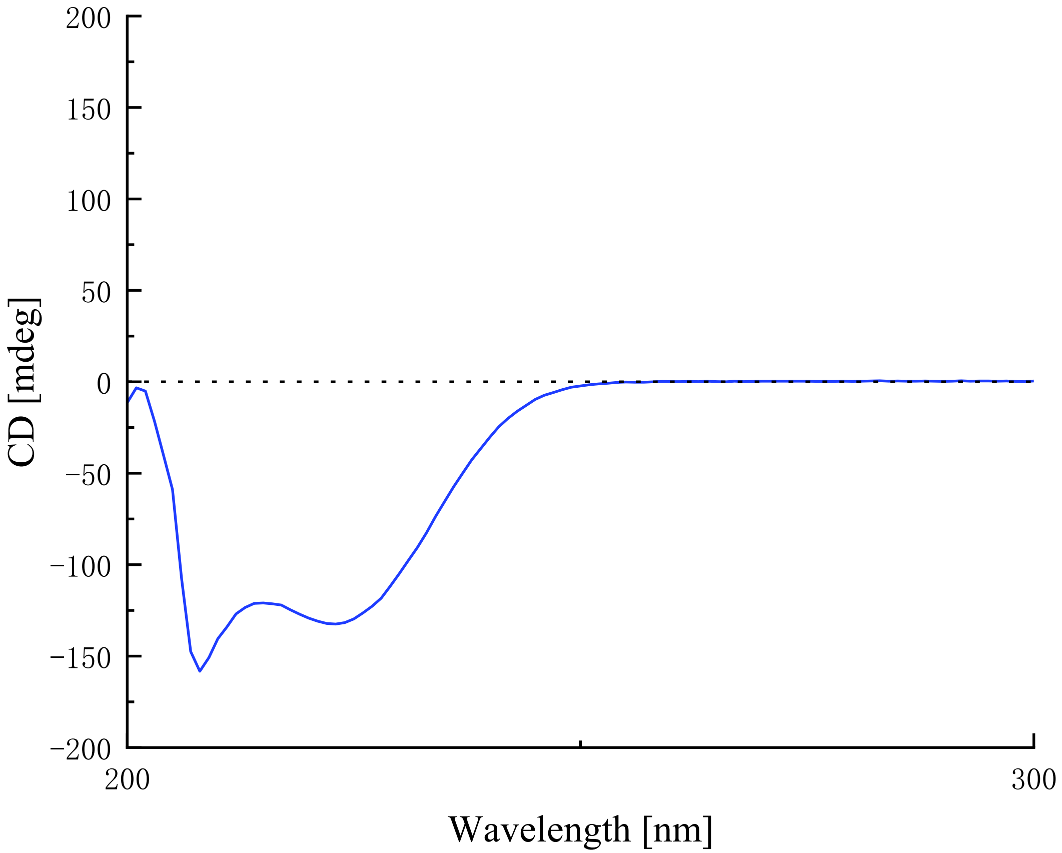

Fig S21. CD spectrum of compound 2 in MeOH

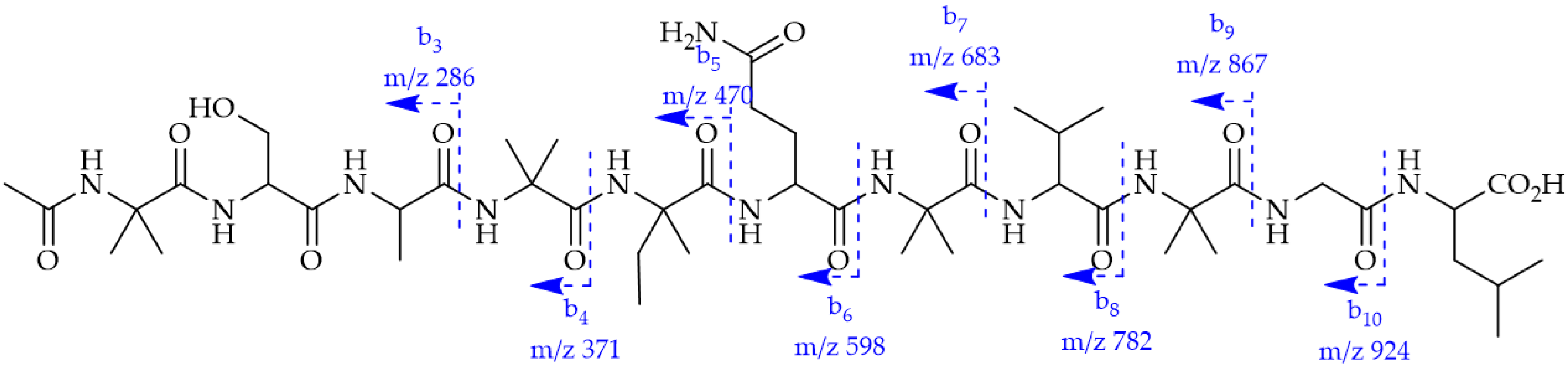

3: Ac-Aib[1]-Ser[2]-Ala[3]-Aib[4]-Iva[5]-Gln[6]-Aib[7]-Val[8]-Aib[9]-Gly[10]-Leu[11]

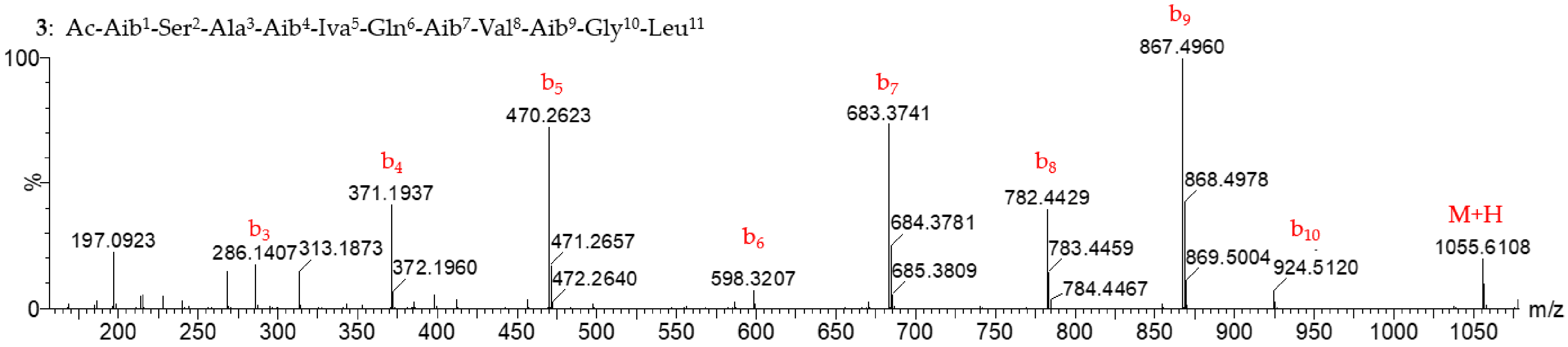

Fig S22. ESI-MS² spectrum of compound 3 at m/z 1055.6 [M+H]⁺

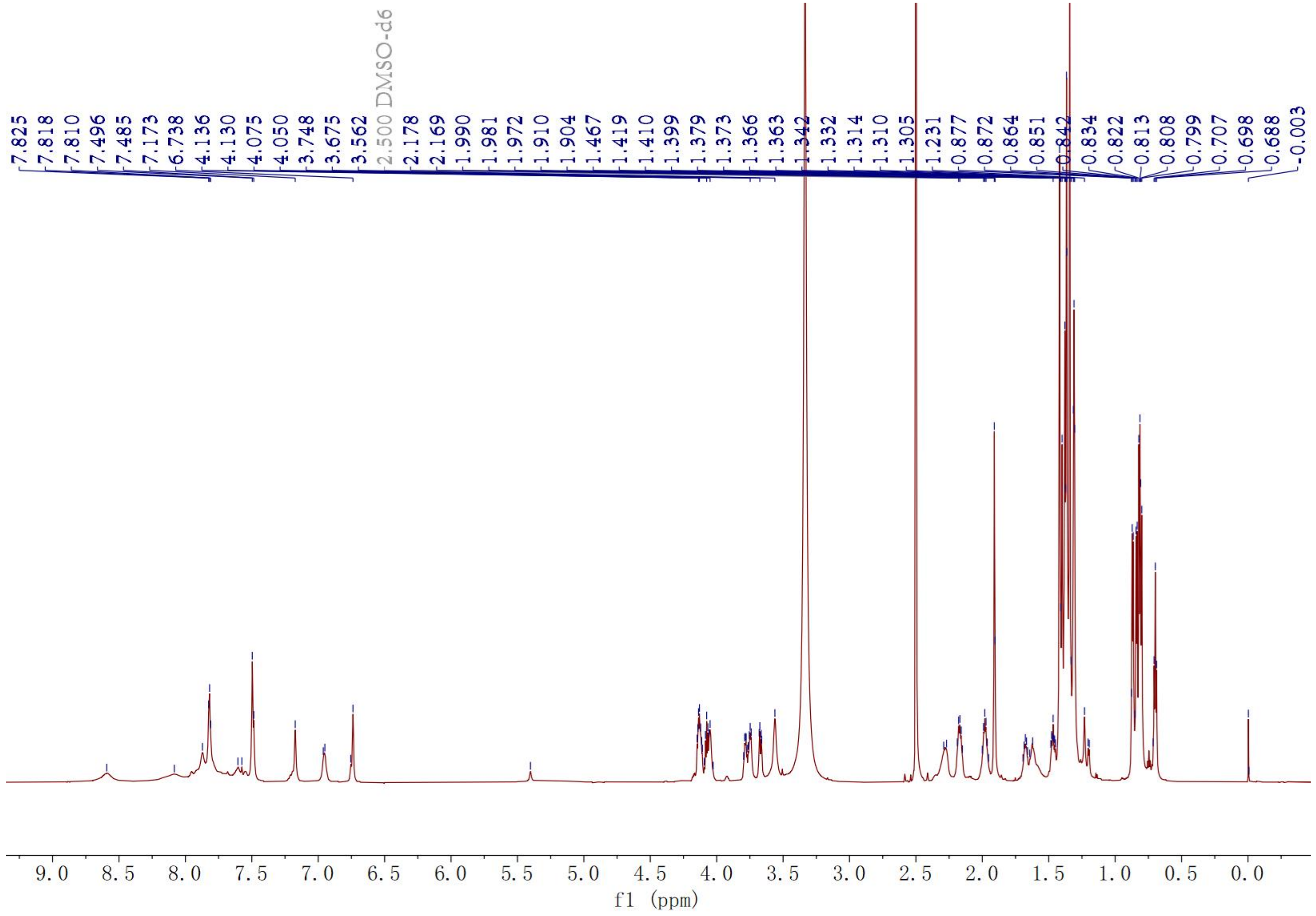

Fig S23. ¹H NMR spectrum of compound 3 in DMSO-*d*₆ (600 MHz)

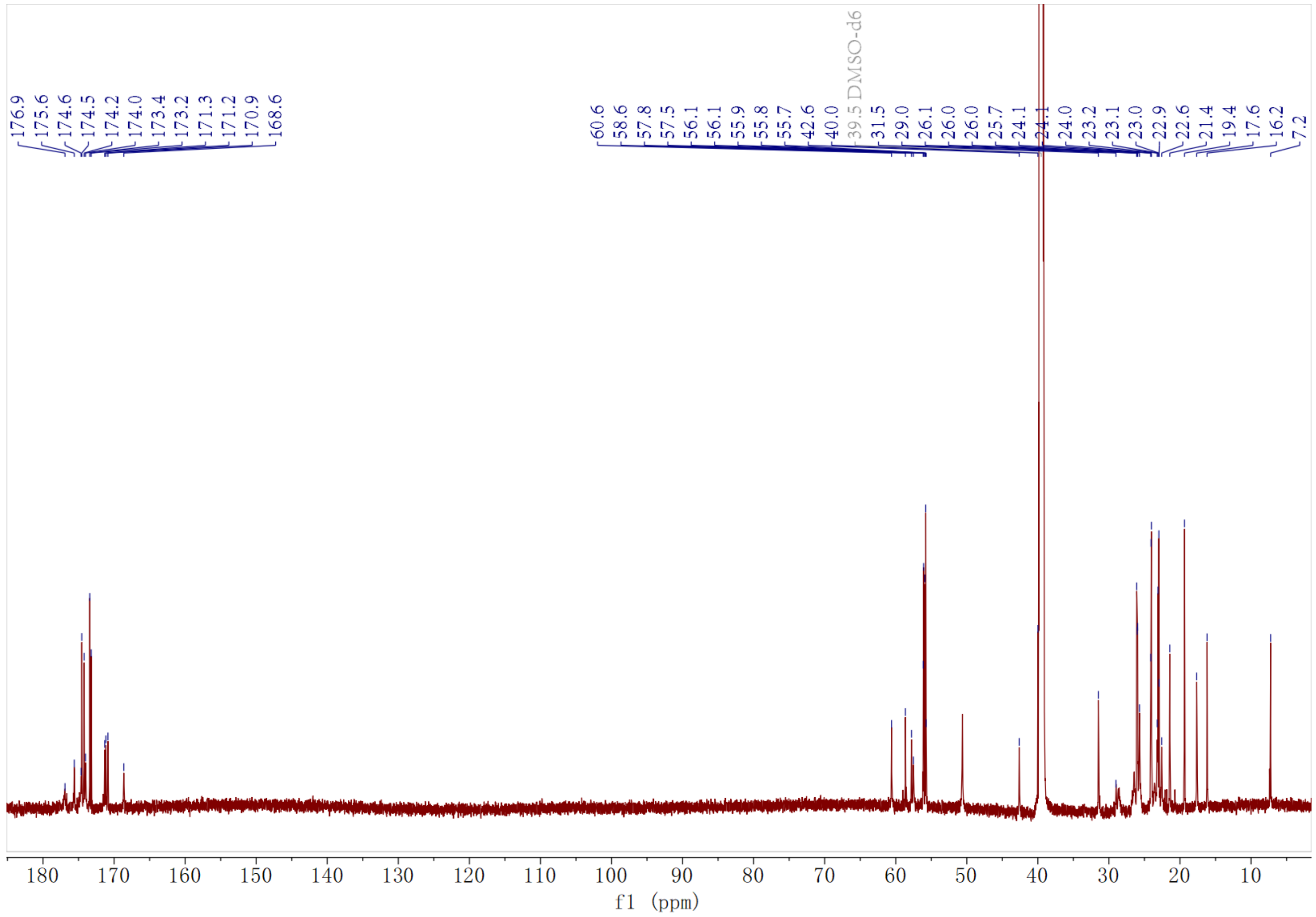

Fig S24. ^{13}**C NMR spectrum of compound 3 in DMSO-*d*$_6$ (600 MHz)**

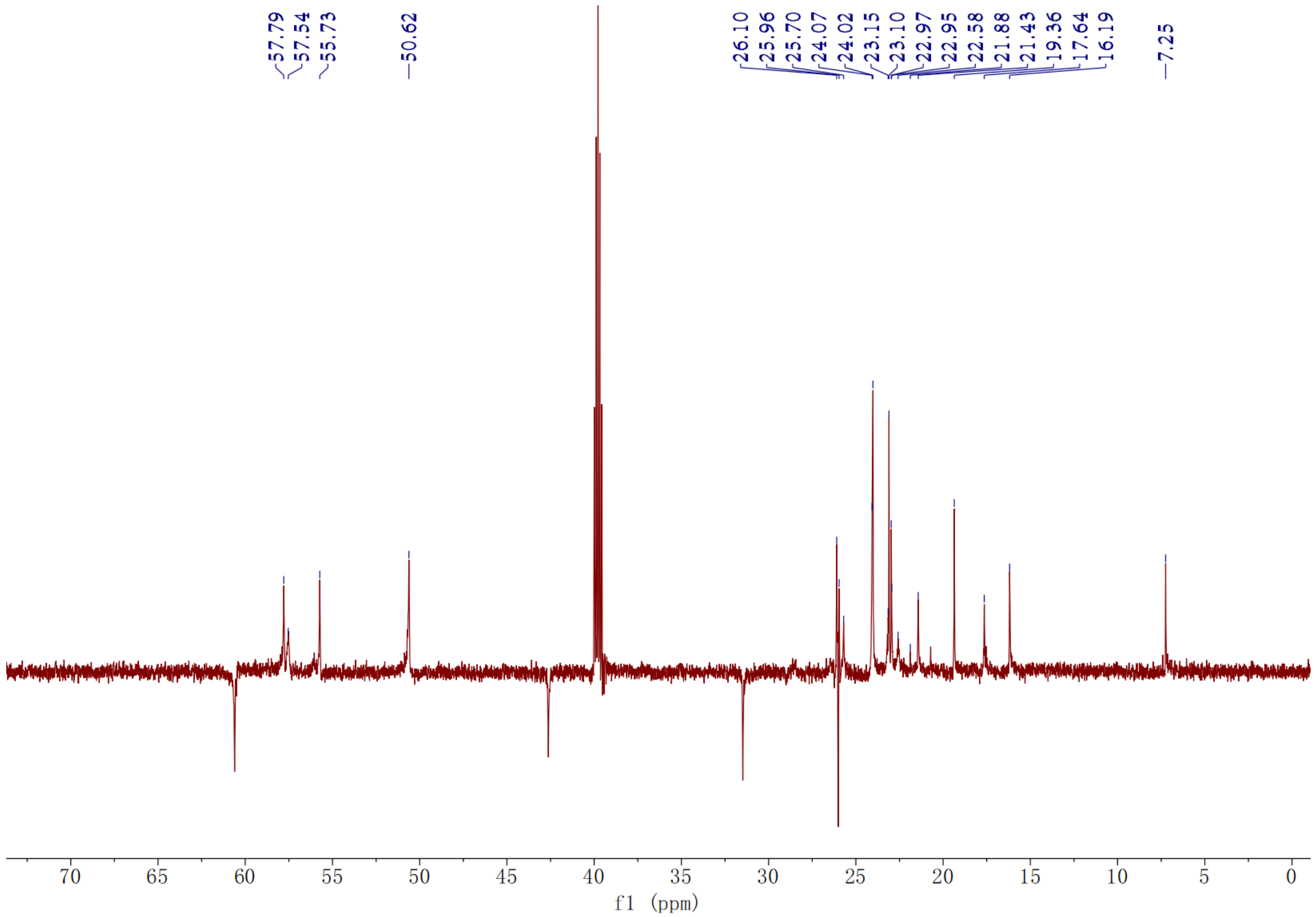

Fig S25. DEPT-135 spectrum of compound 3 in DMSO-d_6 (600 MHz)

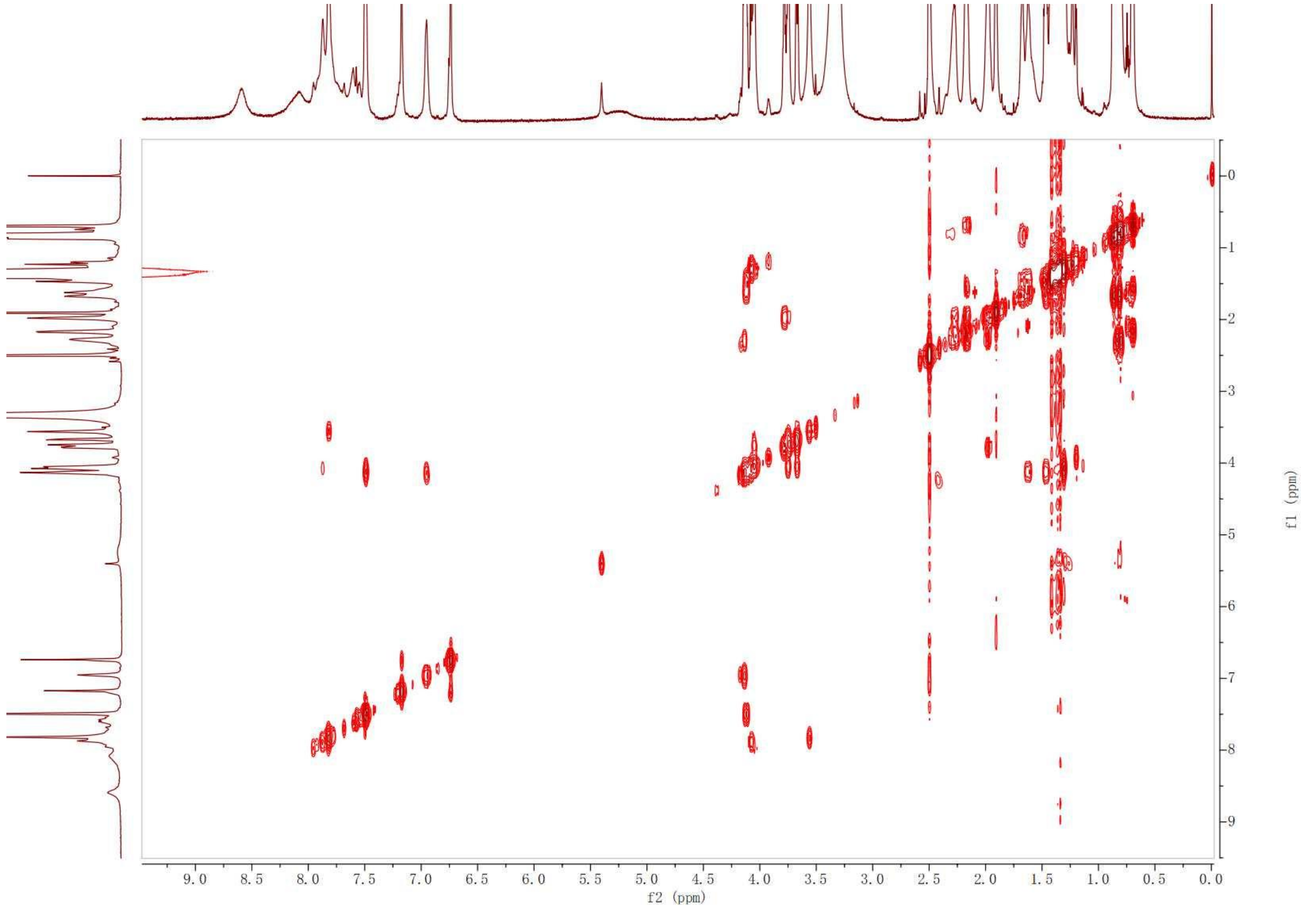

Fig S26. ^{1}**H-**^{1}**H COSY spectrum of compound 3 in DMSO-*****d*****$_6$ (600 MHz)**

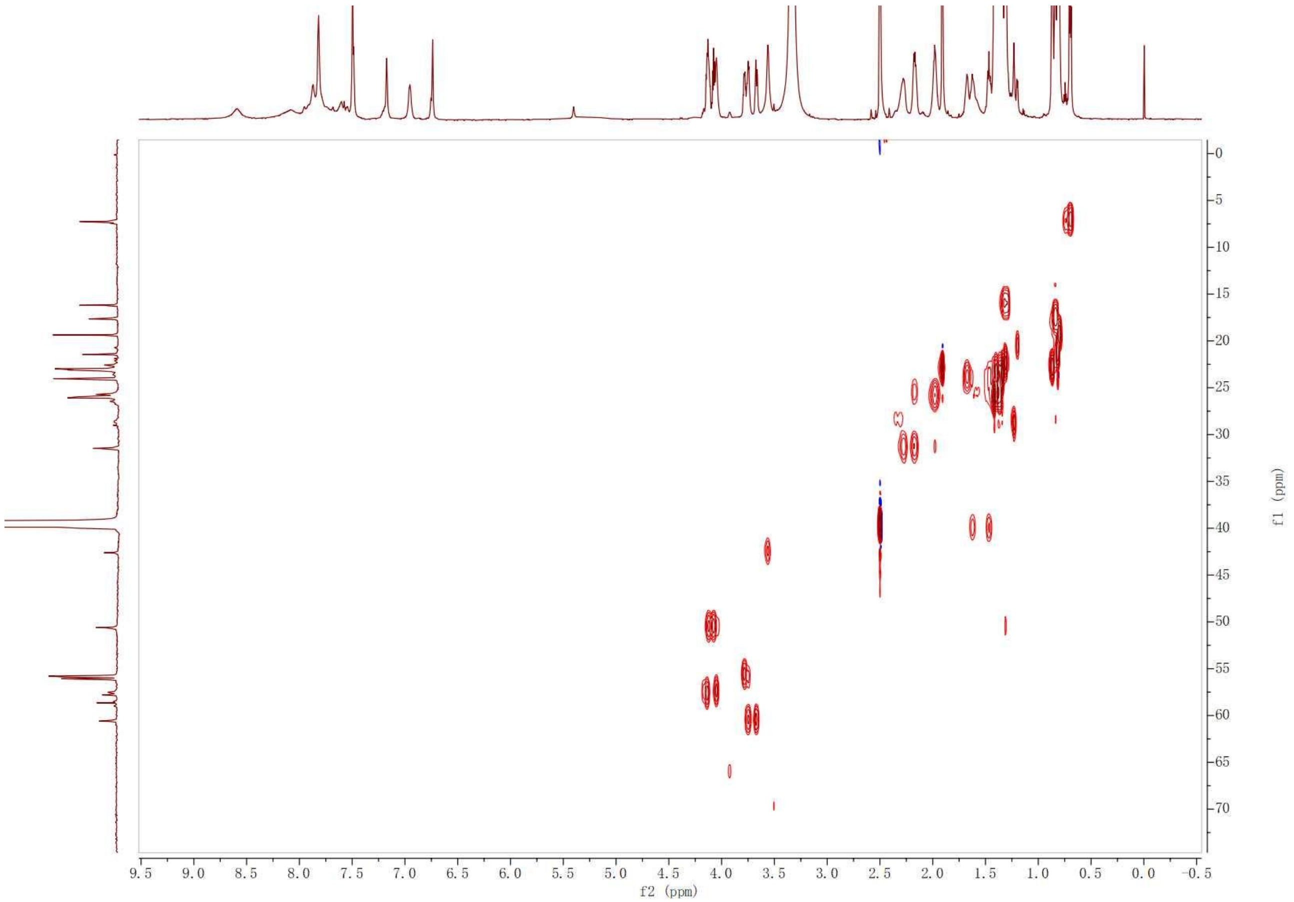

Fig S27. HSQC spectrum of compound 3 in DMSO-d_6 (600 MHz)

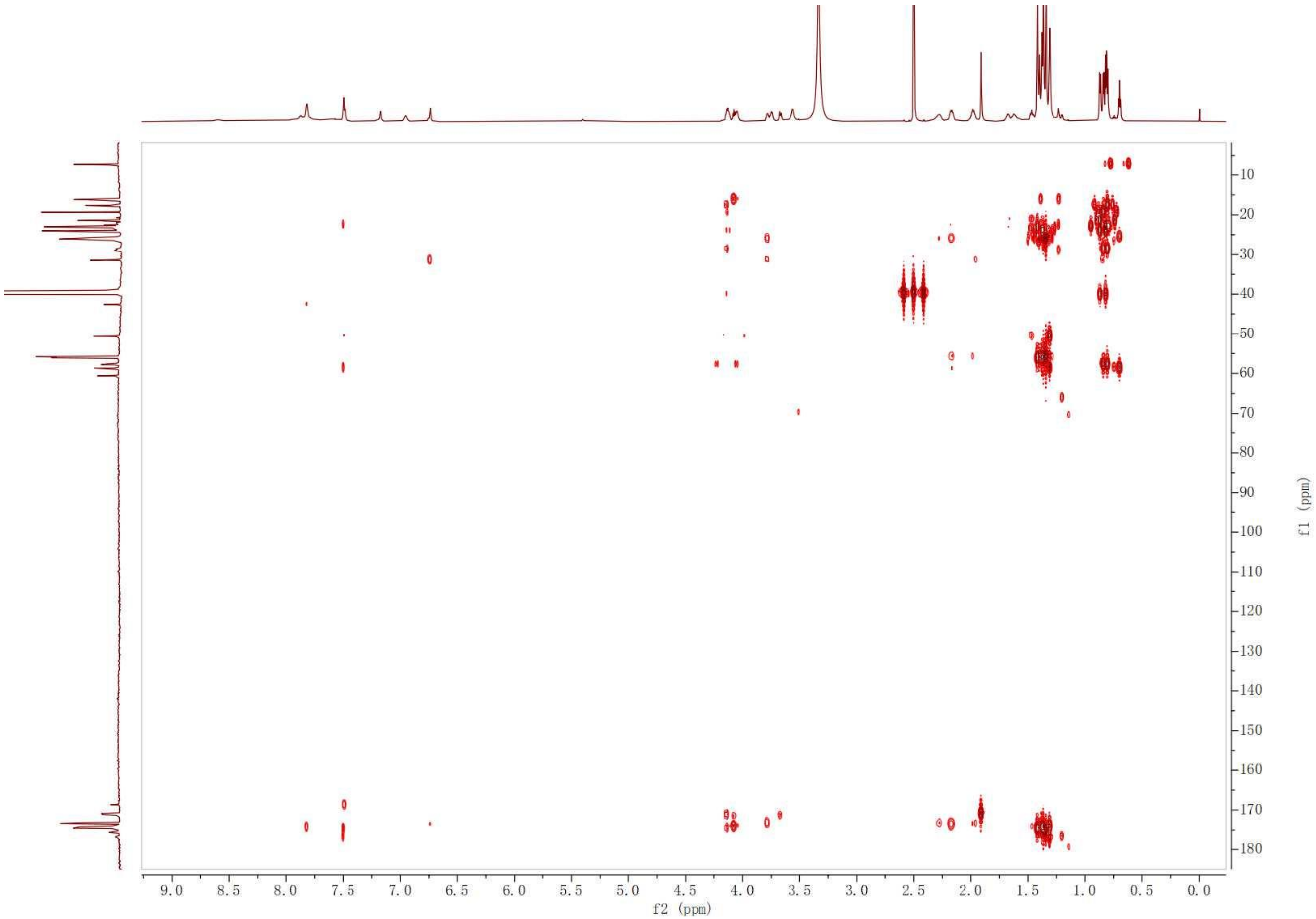

Fig S28. HMBC spectrum of compound 3 in DMSO-d_6 (600 MHz)

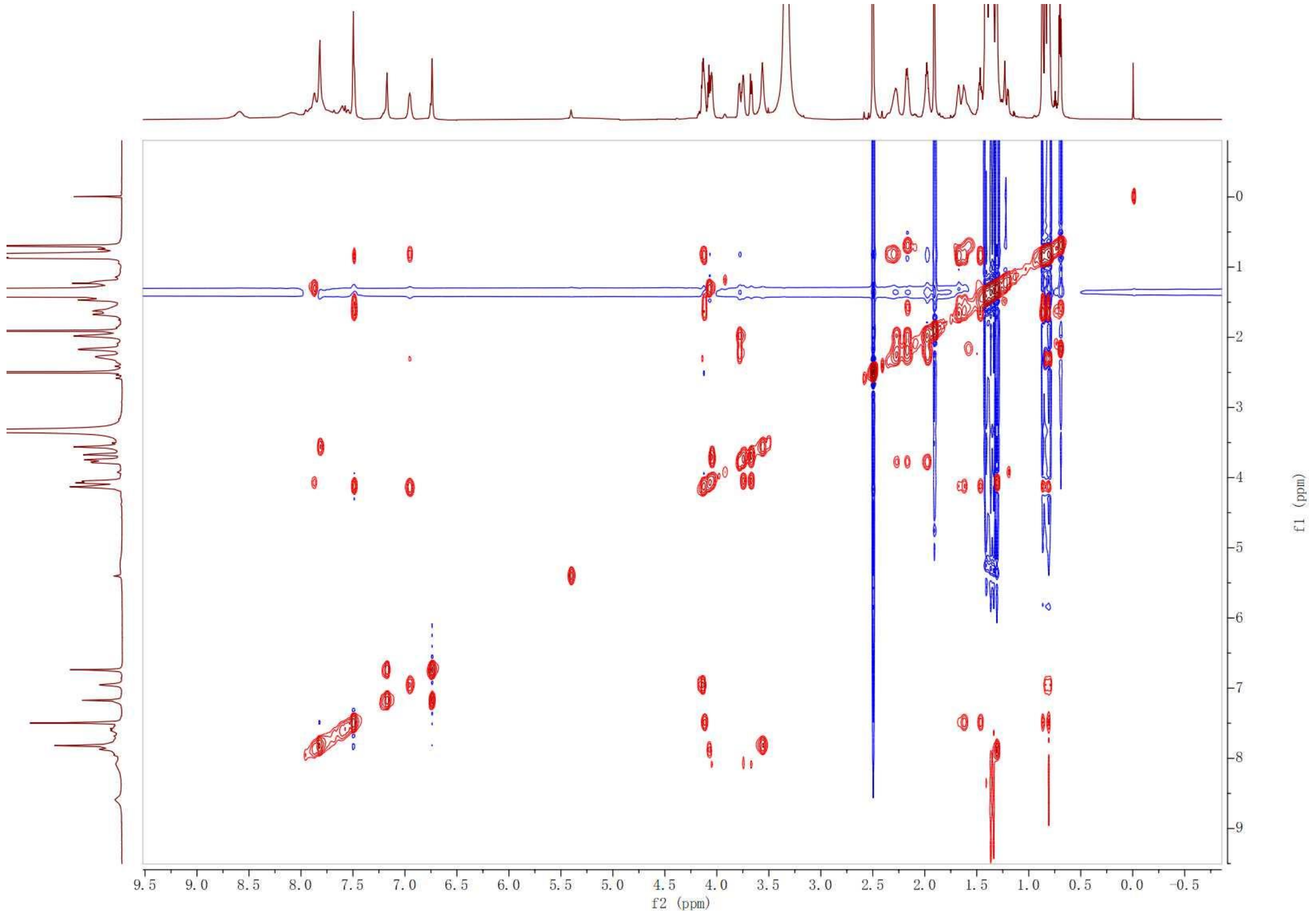

Fig S29. TOCSY spectrum of compound 3 in DMSO-d_6 (600 MHz)

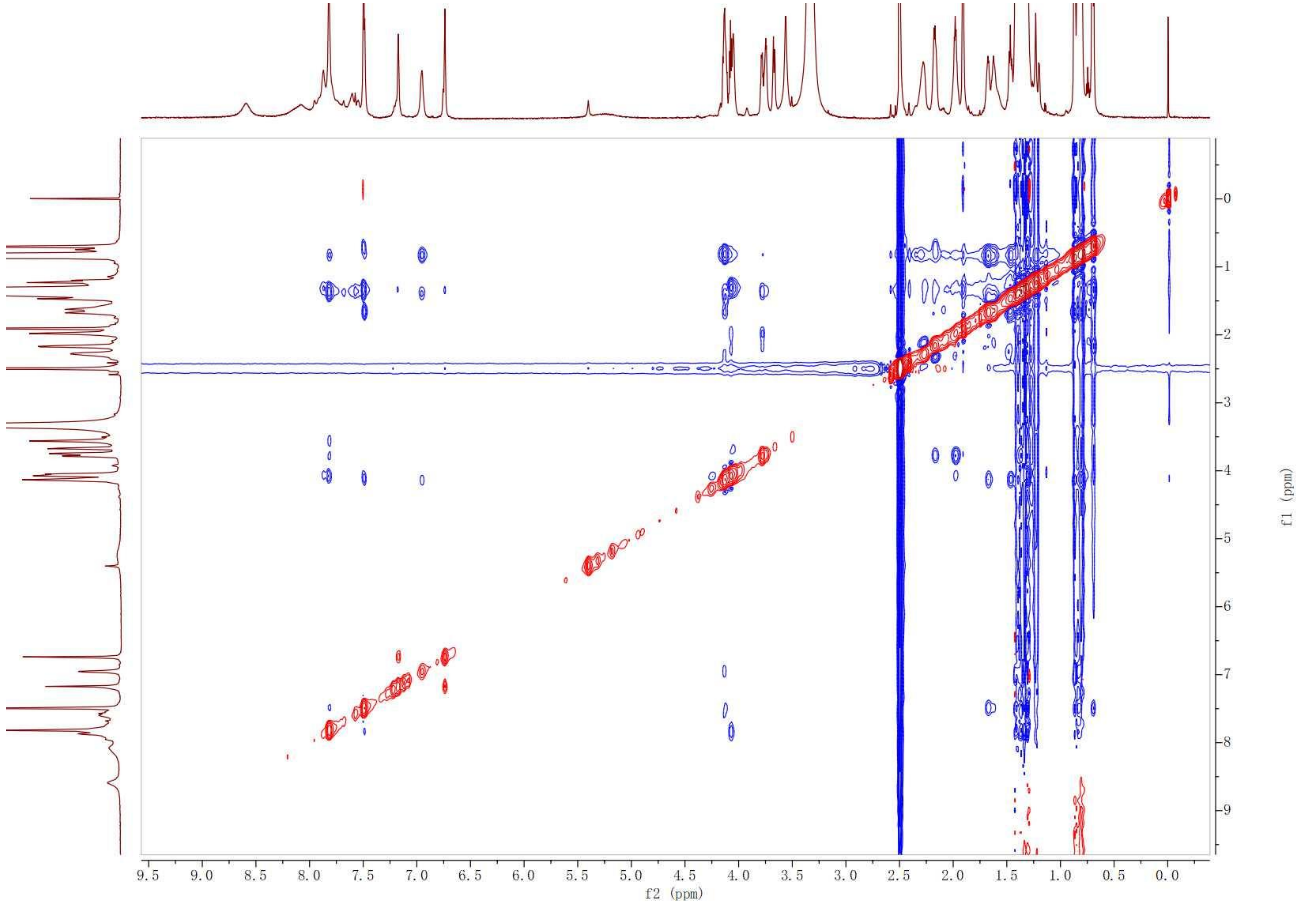

Fig S30. NOESY spectrum of compound 3 in DMSO-*d*$_6$ (600 MHz)

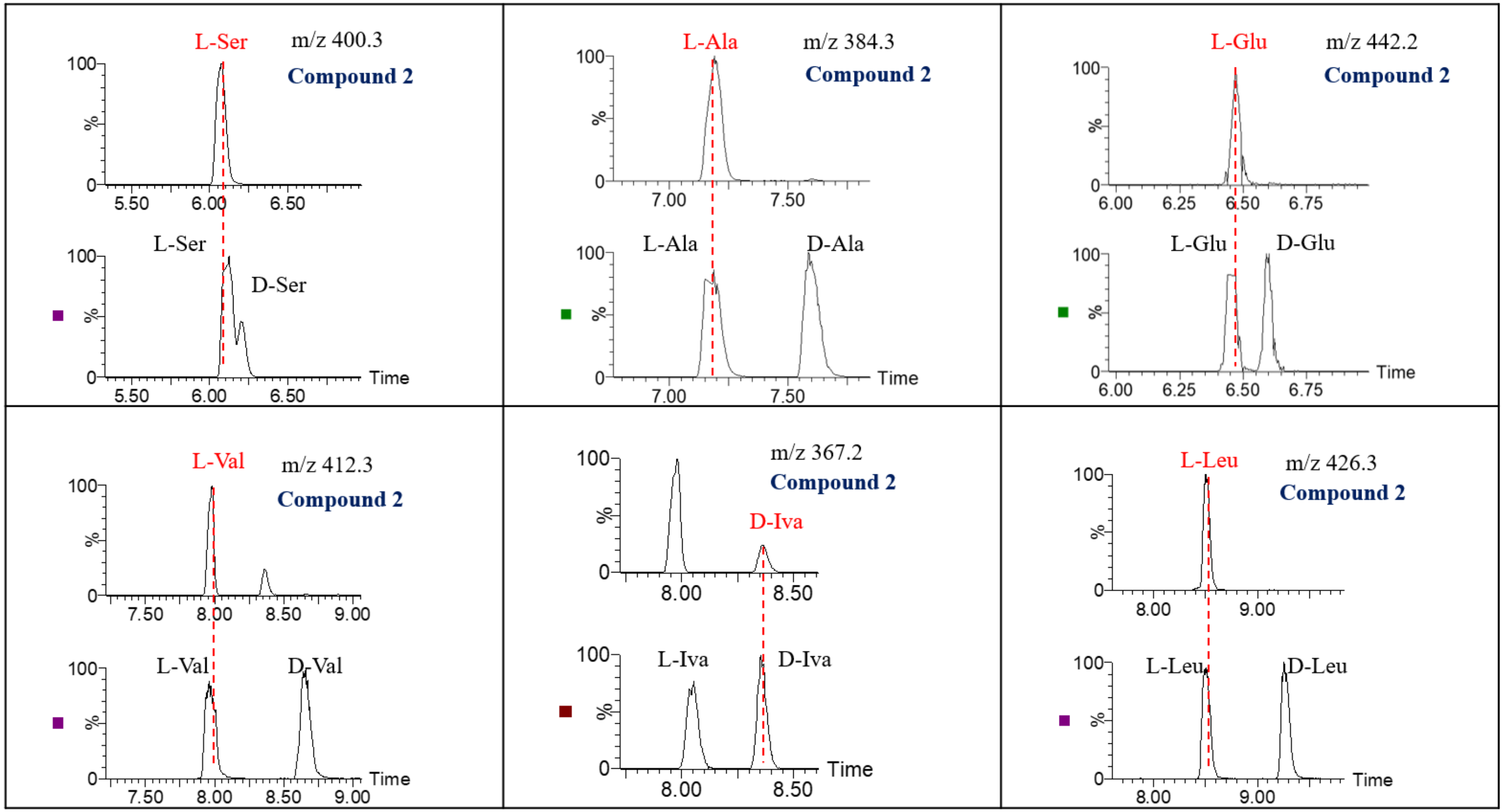

Fig S31. Marfey's of compound 3

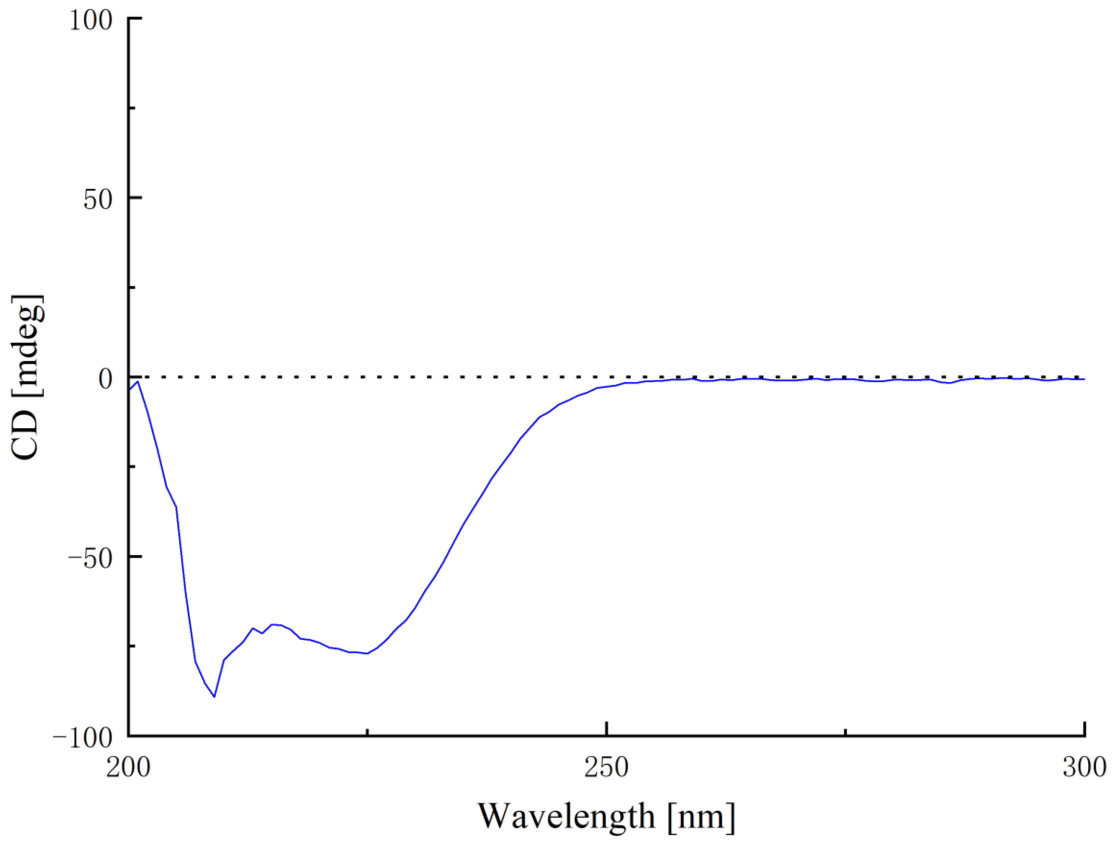

Fig S32. CD spectrum of compound 3 in MeOH

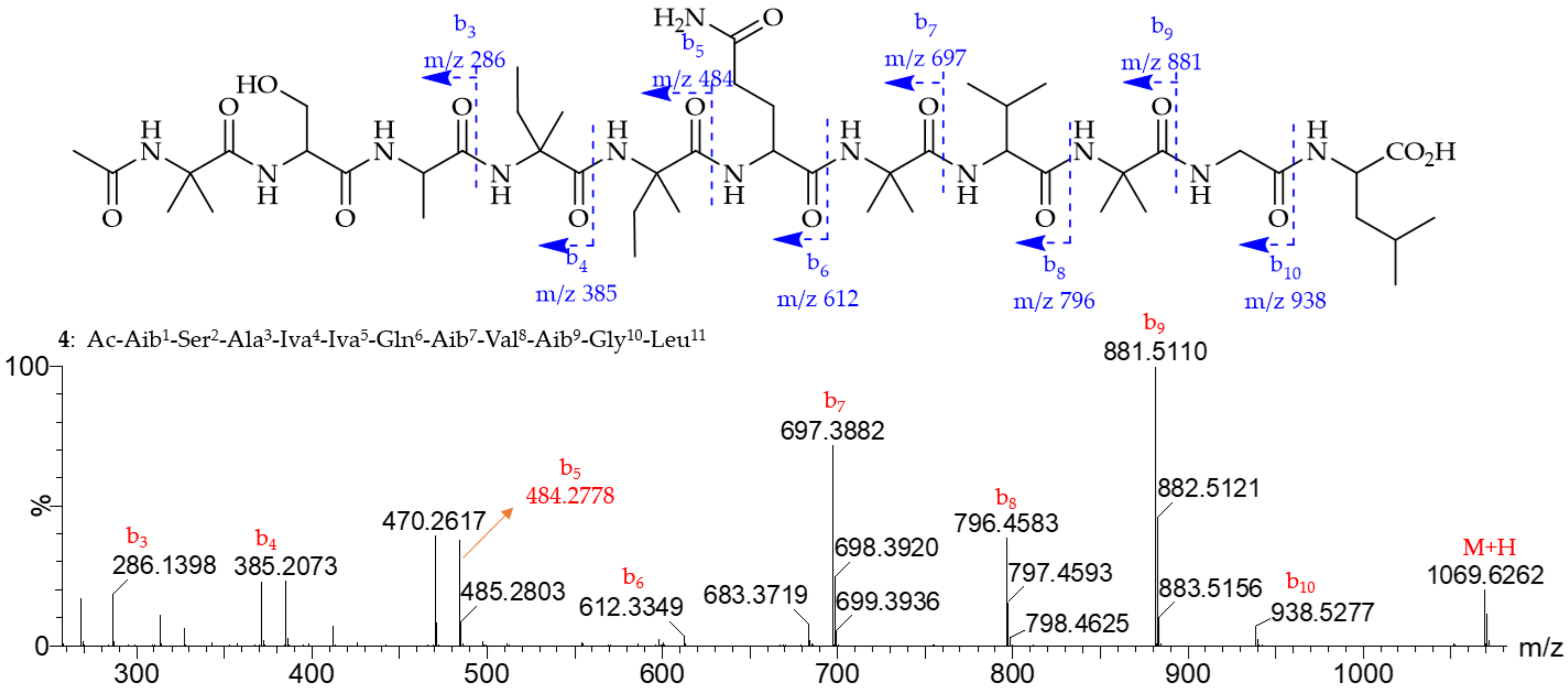

Fig S33. ESI-MS² spectrum of 4 at m/z 1069.6 [M+H]⁺

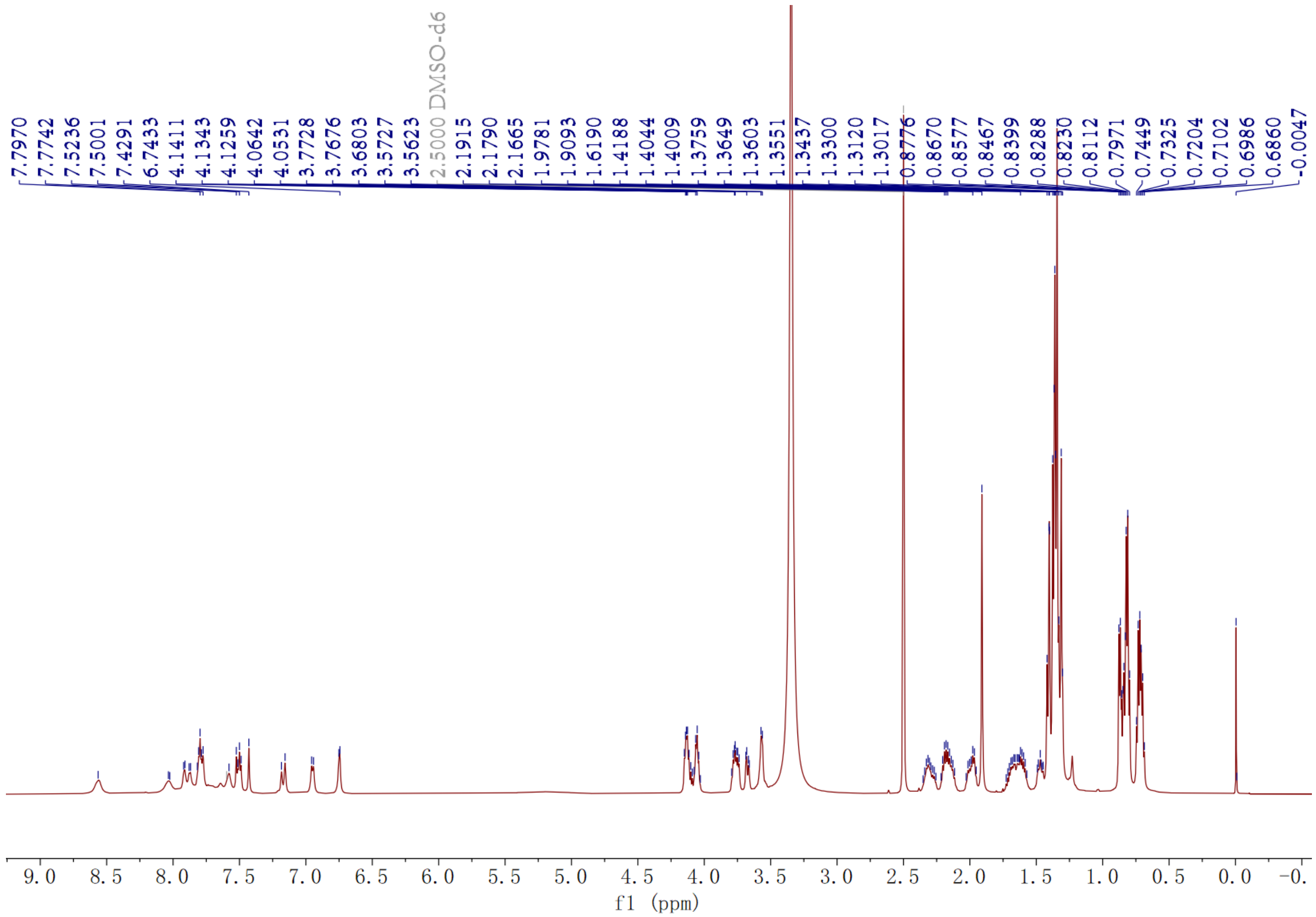

Fig S34. **^{1}H NMR spectrum of compound 4 in DMSO-*d*$_6$ (600 MHz)**

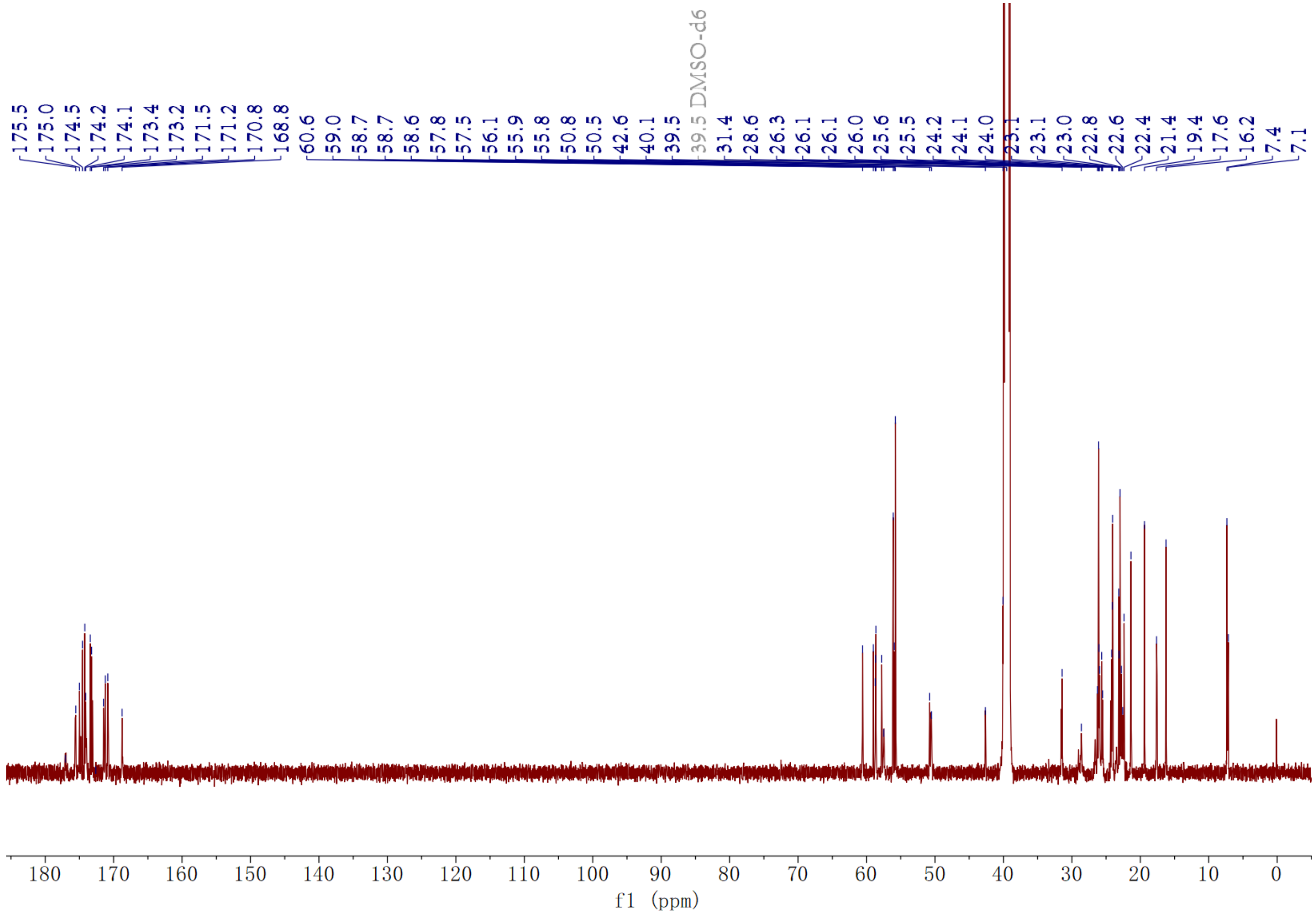

Fig S35. ^{13}**C NMR spectrum of compound 4 in DMSO-*d*$_6$ (600 MHz)**

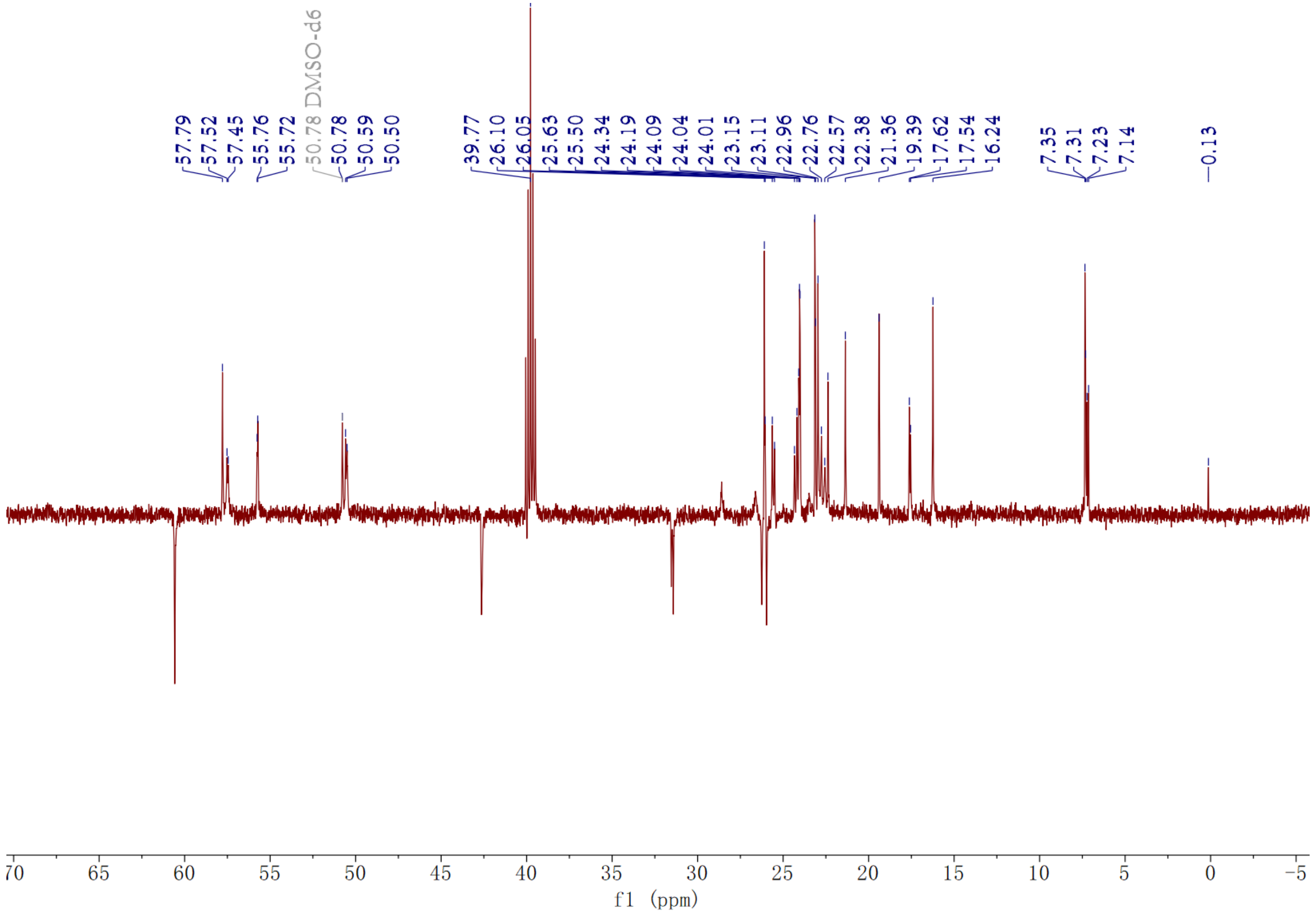

Fig S36. DEPT-135 spectrum of compound 4 in DMSO-d_6 (600 MHz)

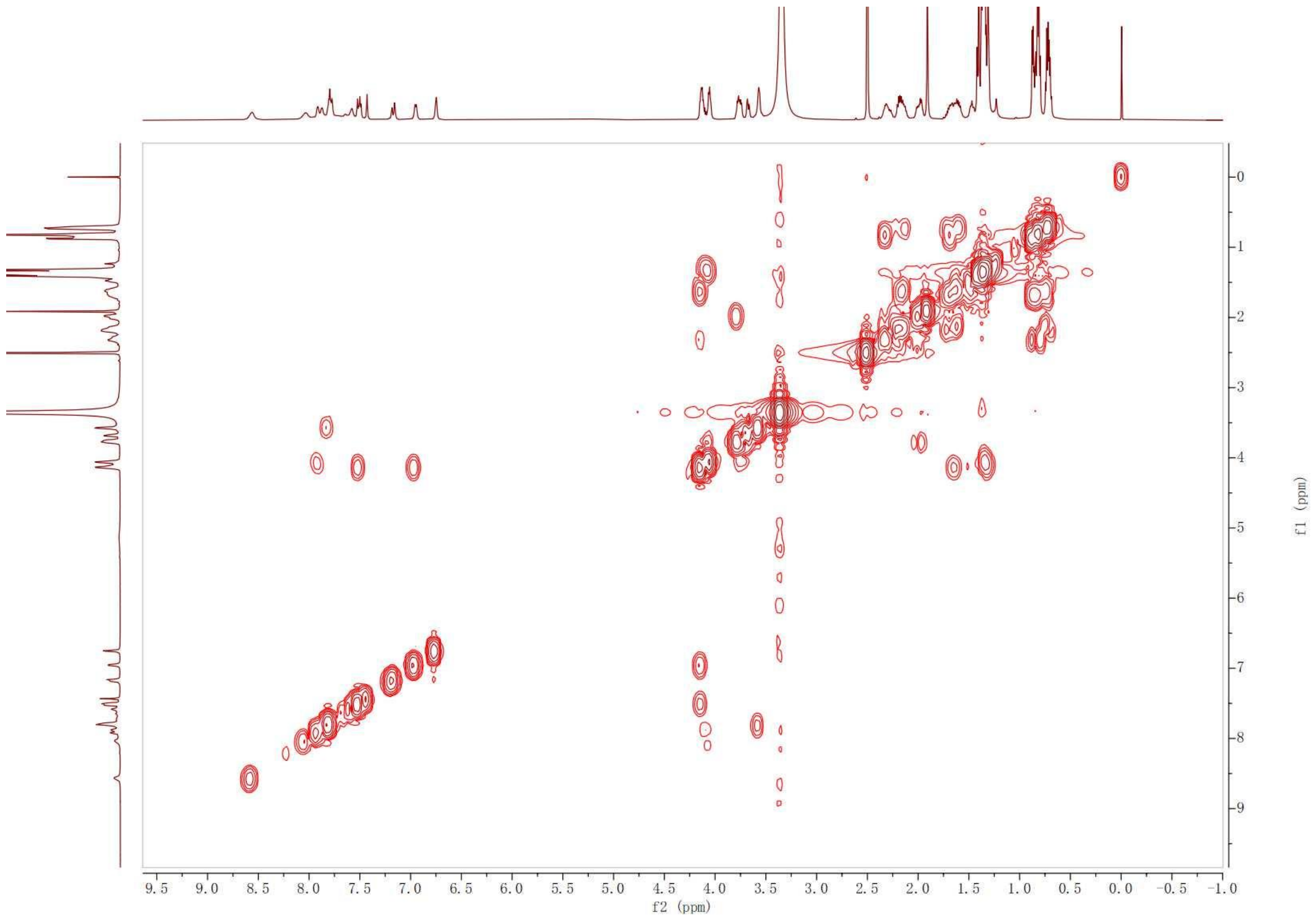

Fig S37. ^{1}H-^{1}H COSY spectrum of compound 4 in DMSO-*d*$_6$ (600 MHz)

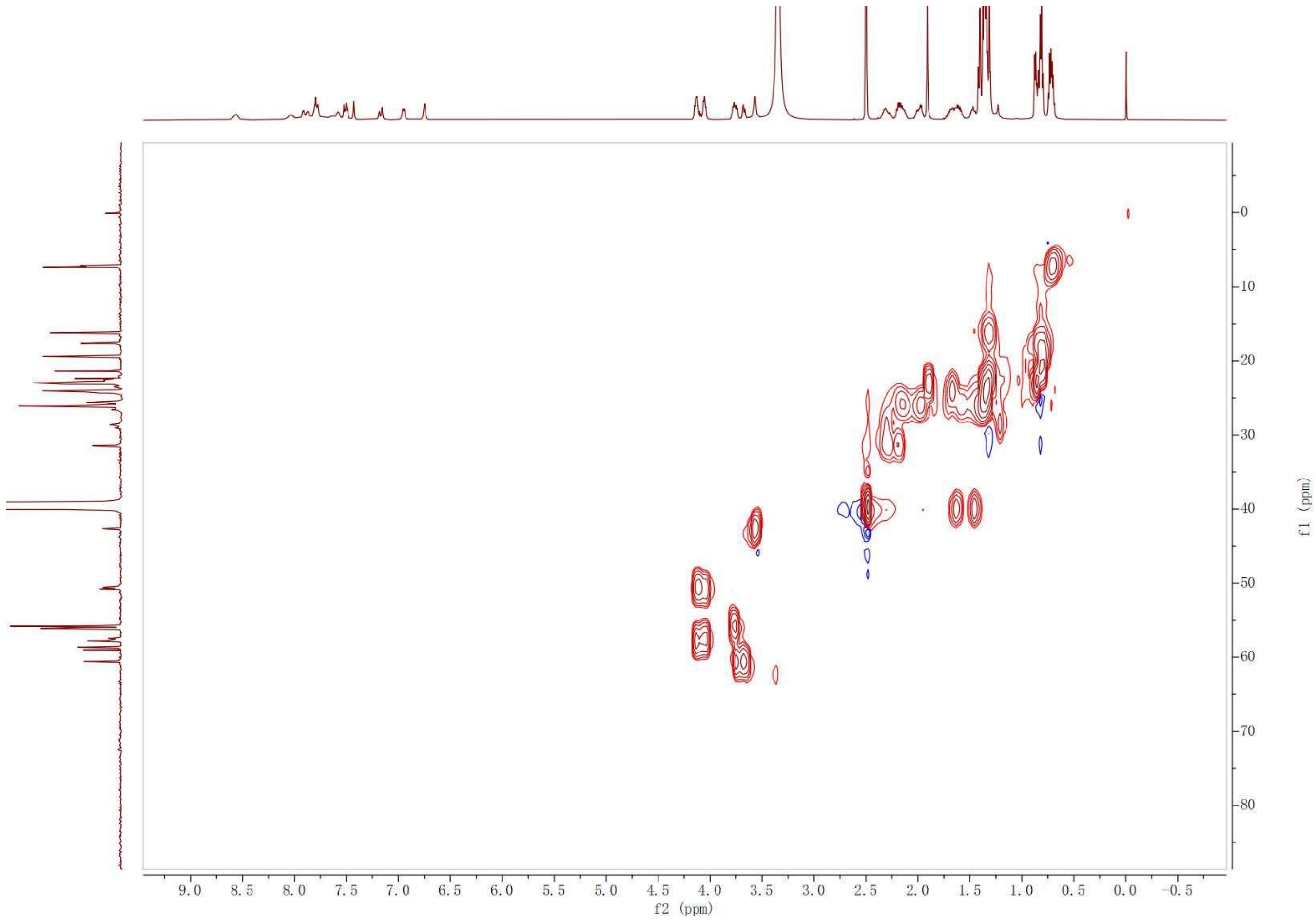

Fig S38. HSQC spectrum of compound 4 in DMSO-d_6 (600 MHz)

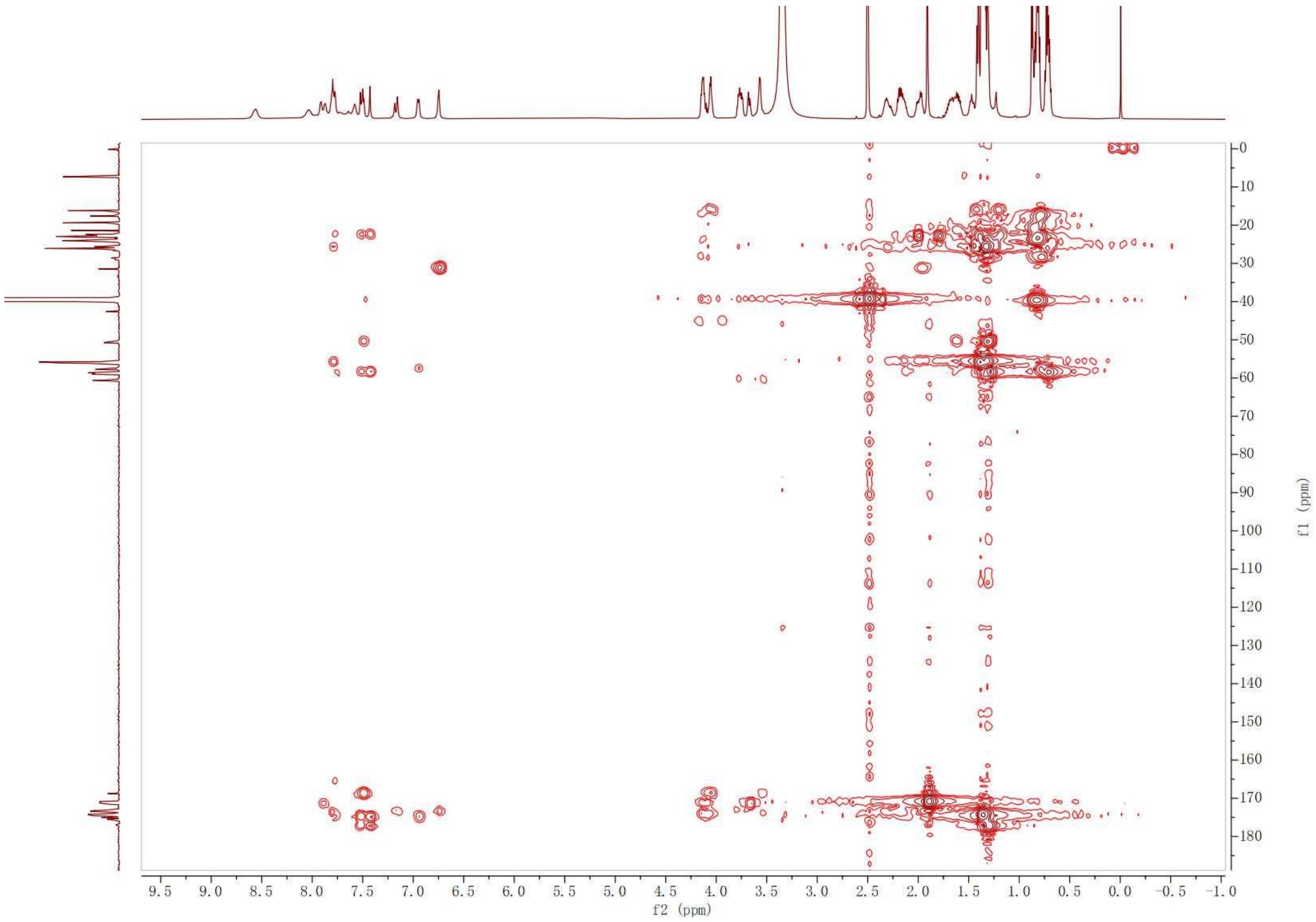

Fig S39. HMBC spectrum of compound 4 in DMSO-d_6 (600 MHz)

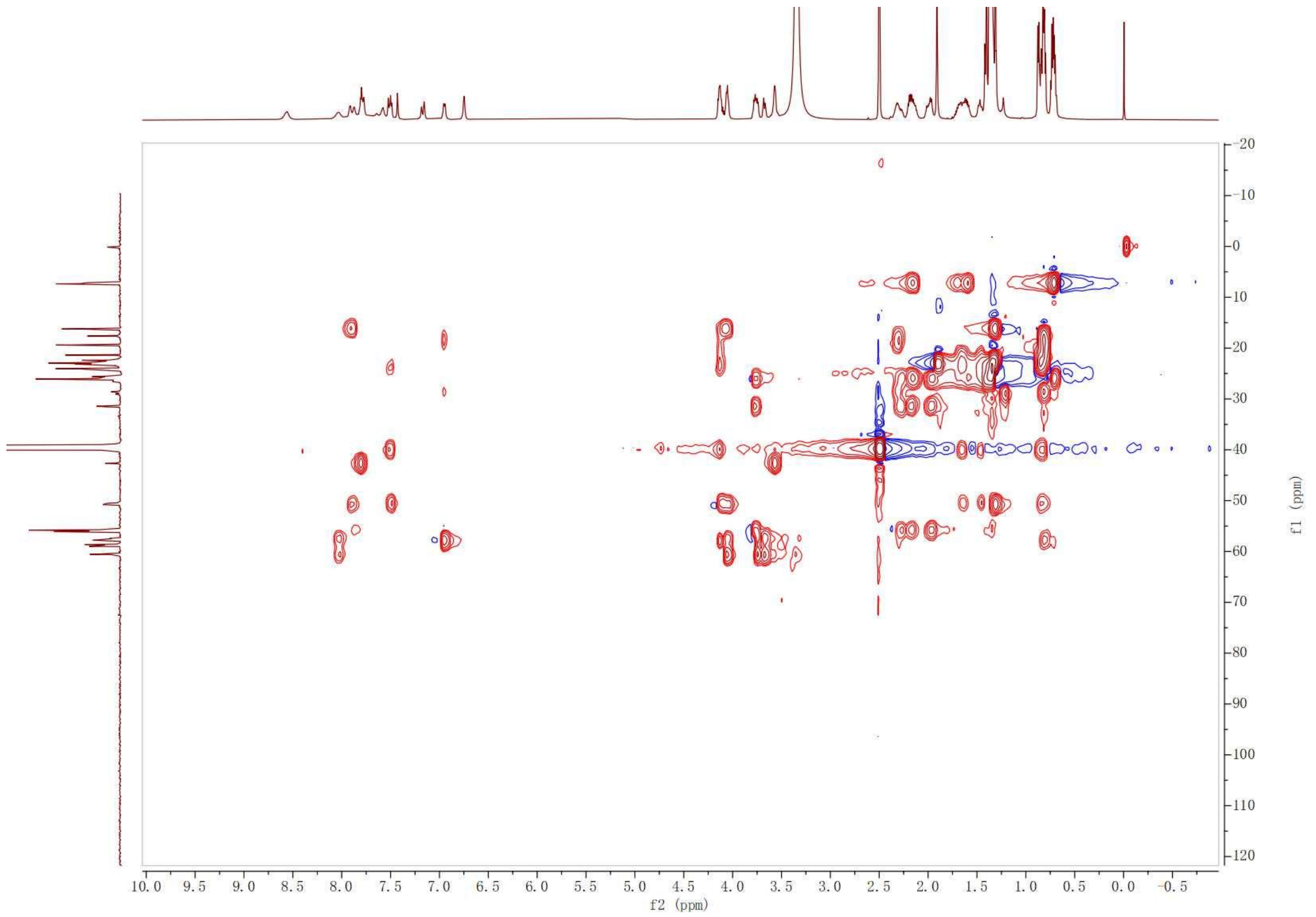

Fig S40. TOCSY spectrum of compound 4 in DMSO-d_6 (600 MHz)

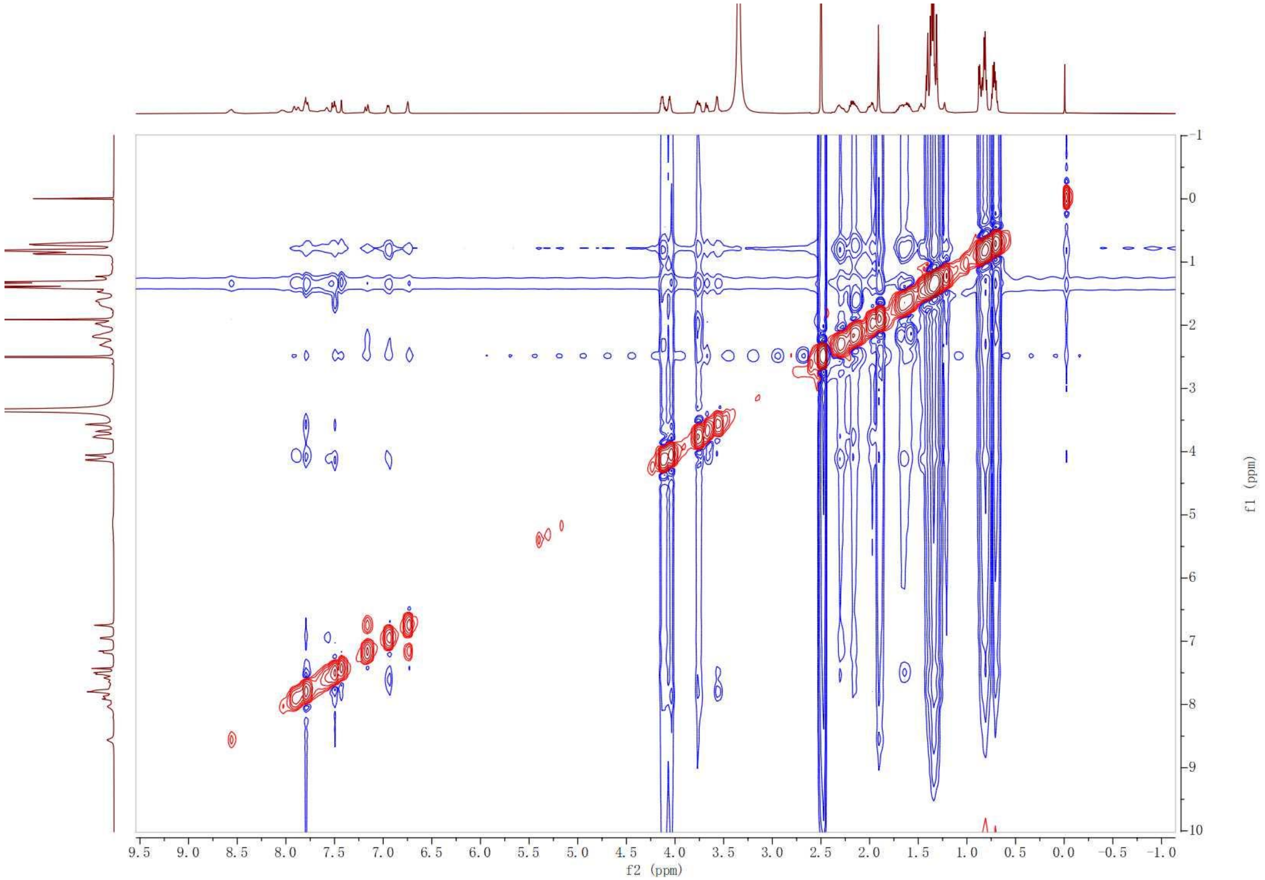

Fig S41. NOESY spectrum of compound 4 in DMSO-d_6 (600 MHz)

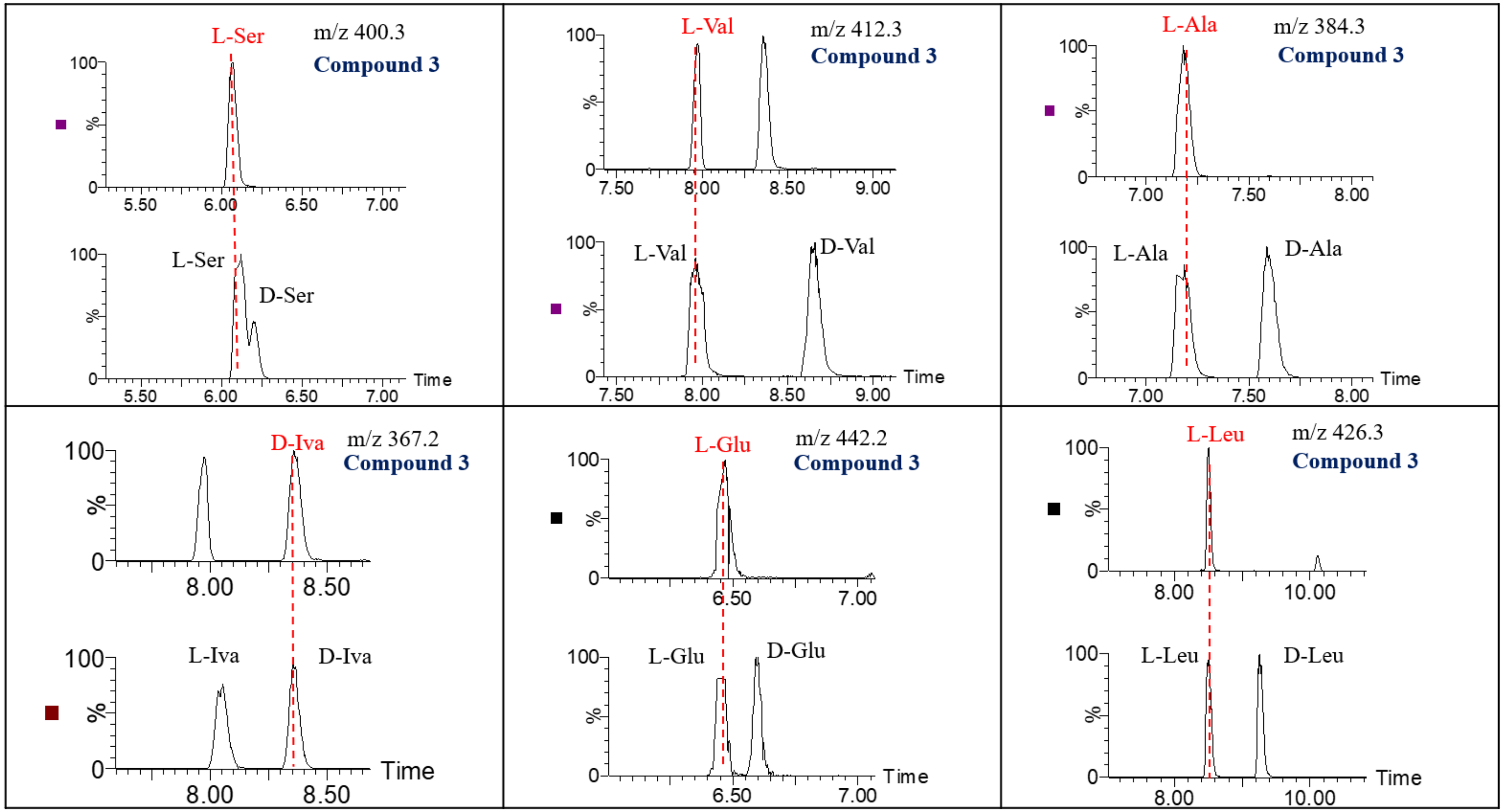

Fig S42. Marfey's of compound 4

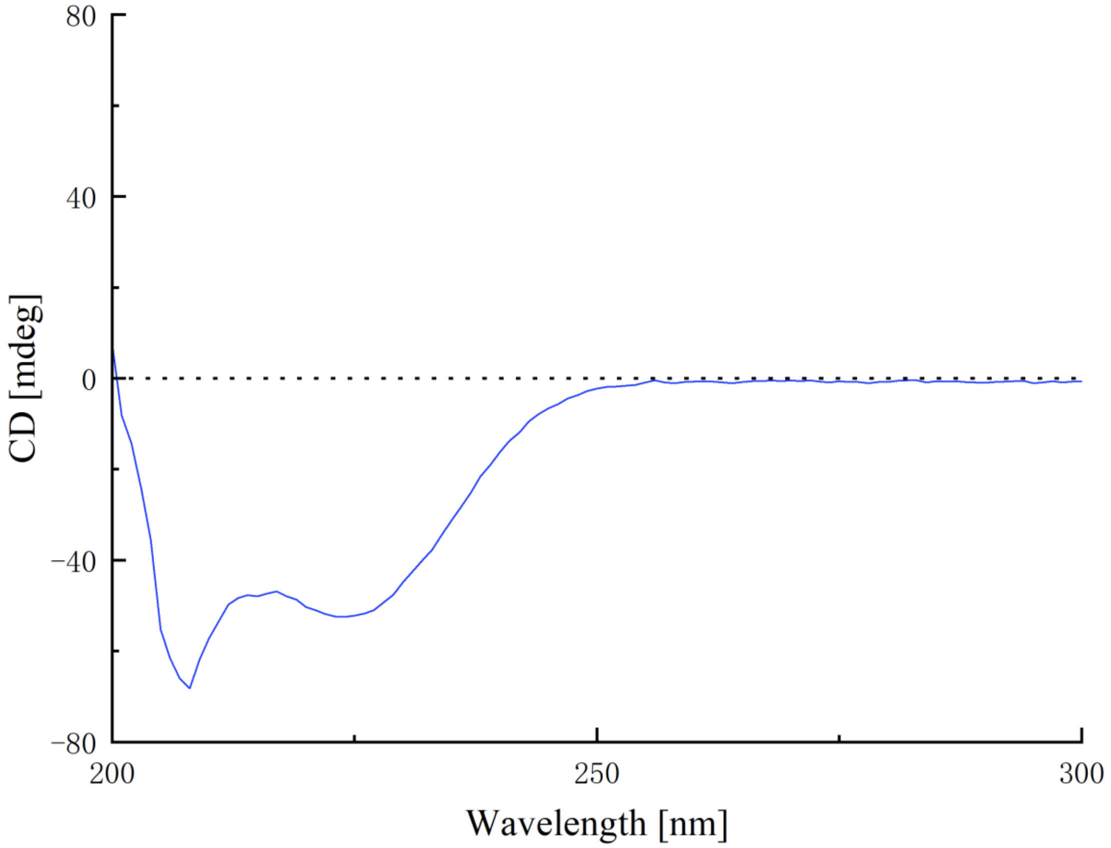

Fig S43. CD spectrum of compound 4 in MeOH

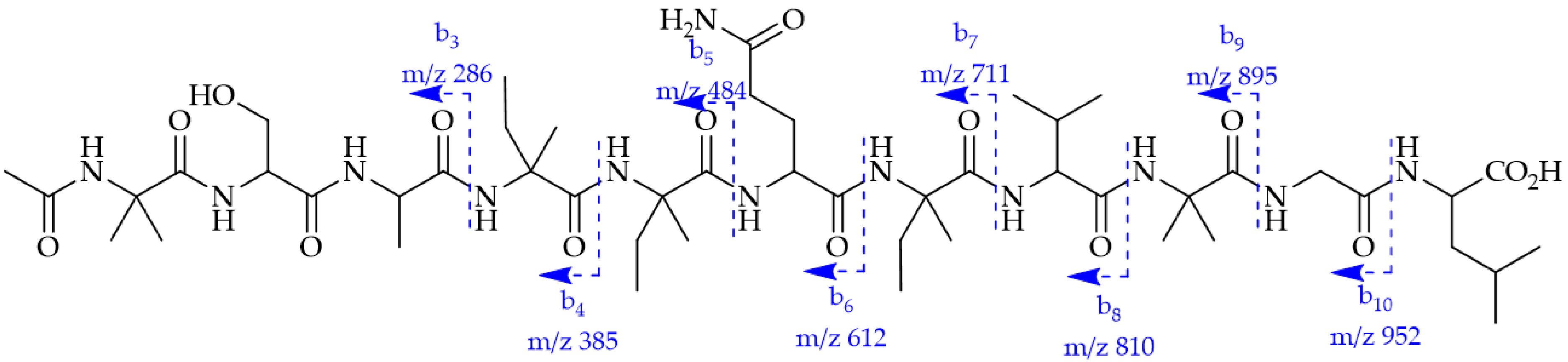

5: Ac-Aib[1]-Ser[2]-Ala[3]-Iva[4]-Iva[5]-Gln[6]-Iva[7]-Val[8]-Aib[9]-Gly[10]-Leu[11]

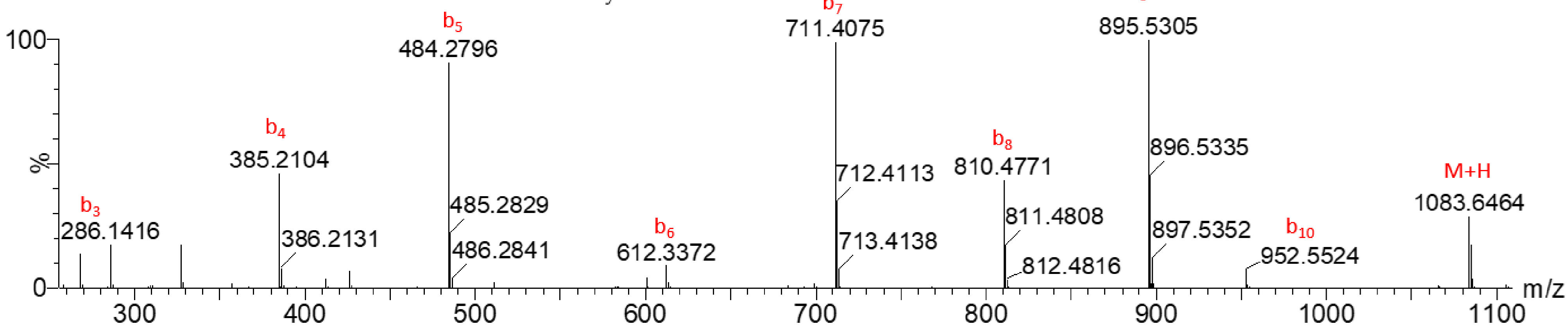

Fig S44. ESI-MS² spectrum of 5 at m/z 1083.6 [M+H]⁺

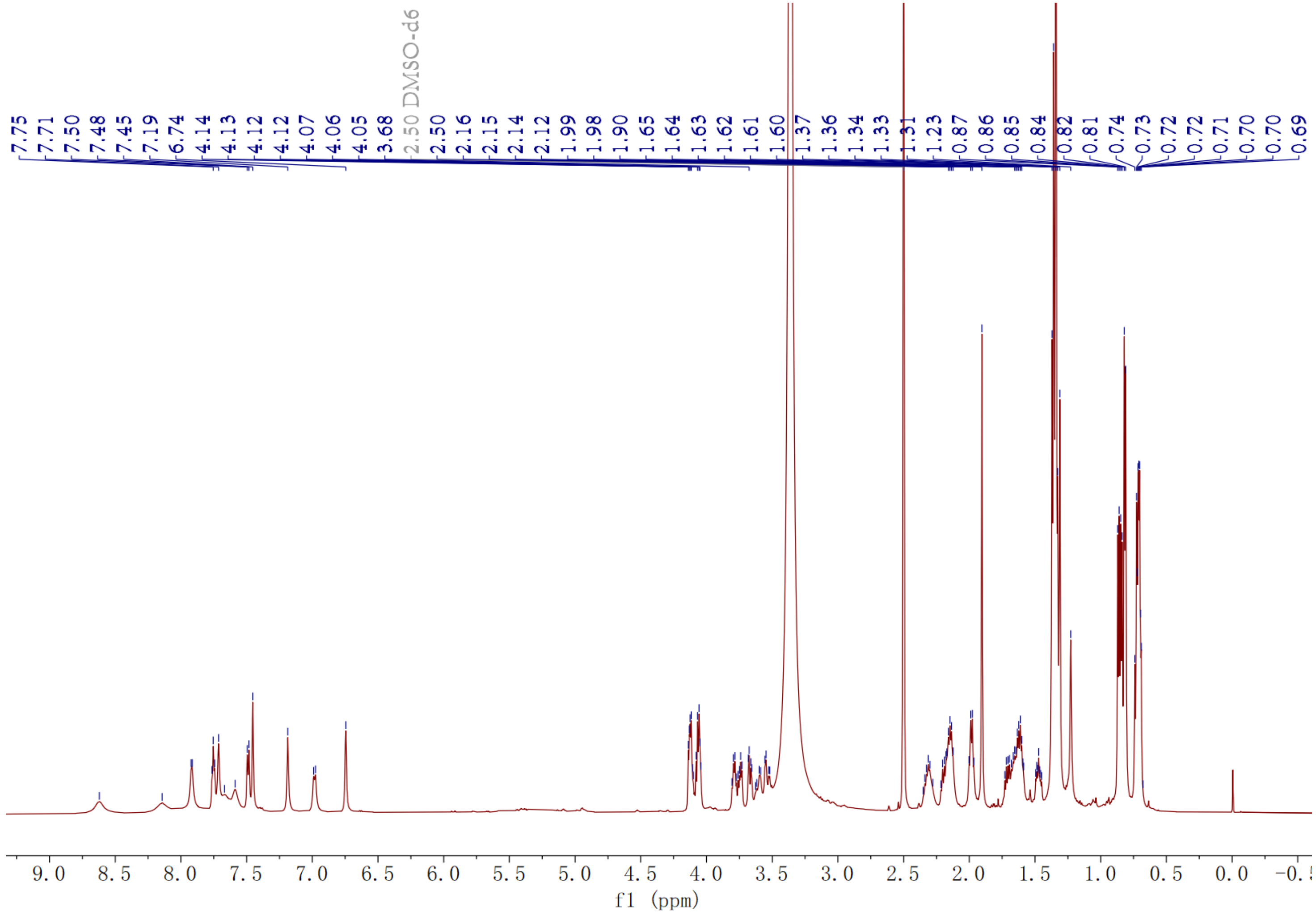

Fig S45. ^{1}H NMR spectrum of compound 5 in DMSO-d_6 (600 MHz)

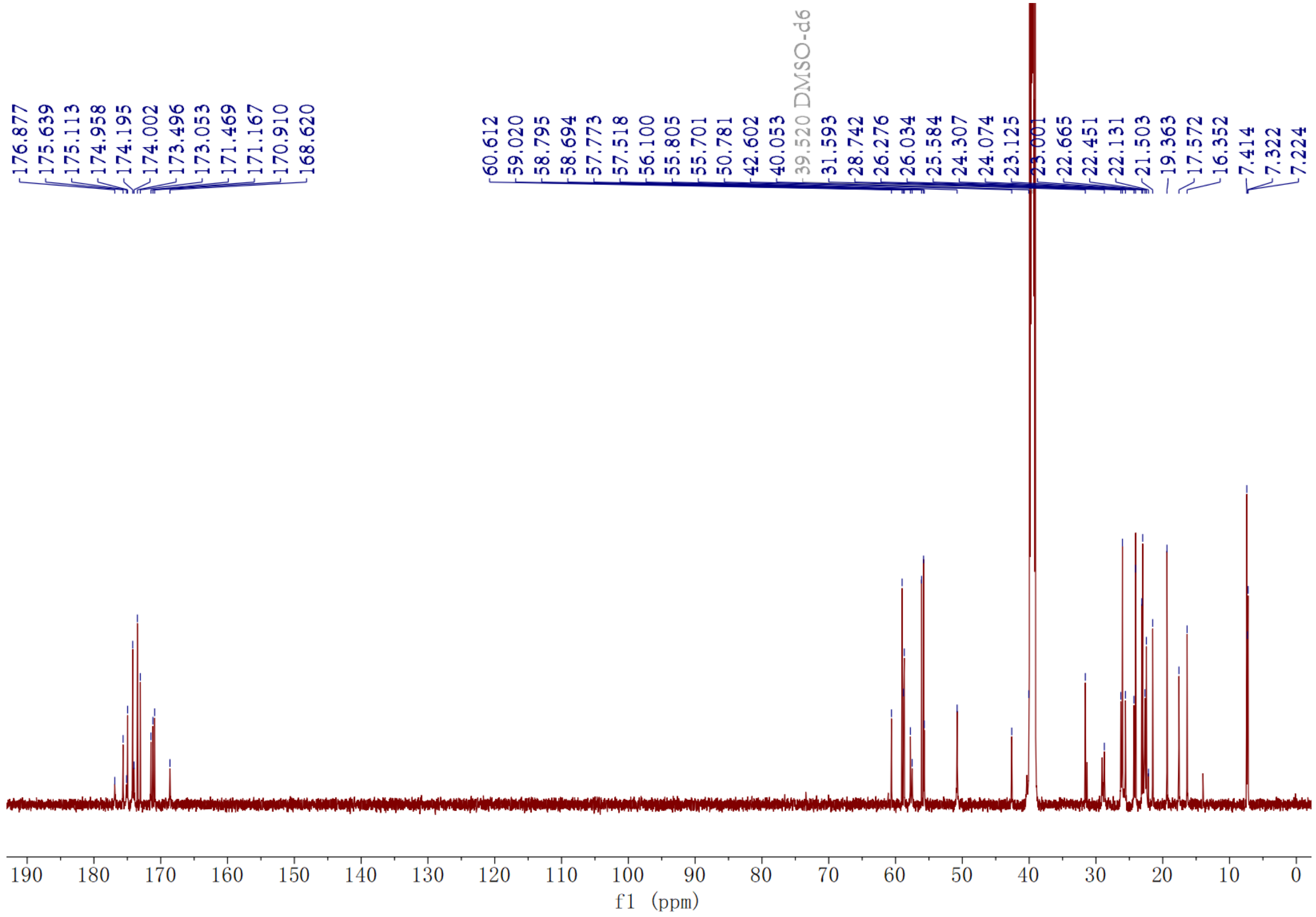

Fig S46. ^{13}C NMR spectrum of compound 5 in DMSO-*d*$_6$ (600 MHz)

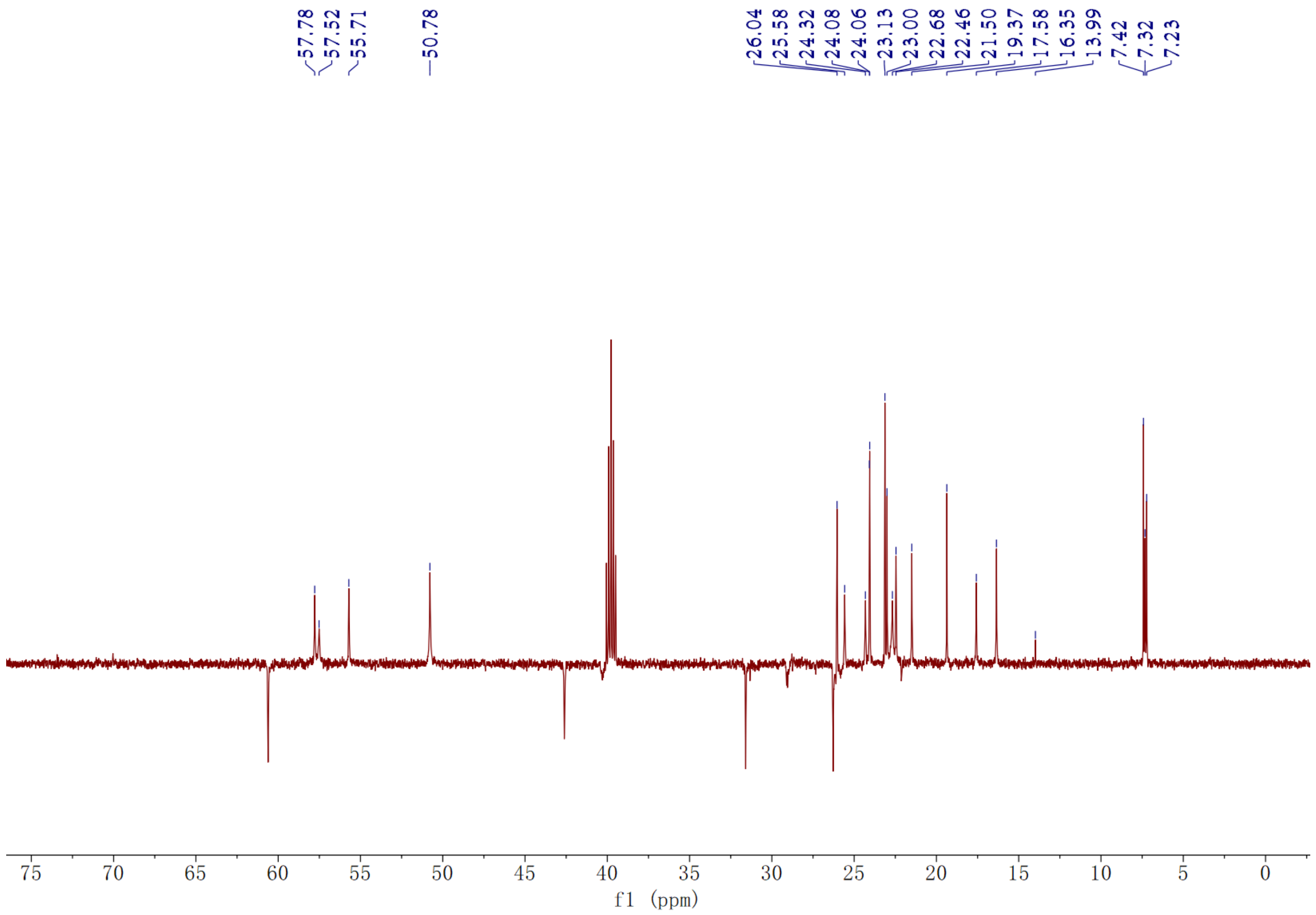

Fig S47. DEPT-135 spectrum of compound 5 in DMSO-d_6 (600 MHz)

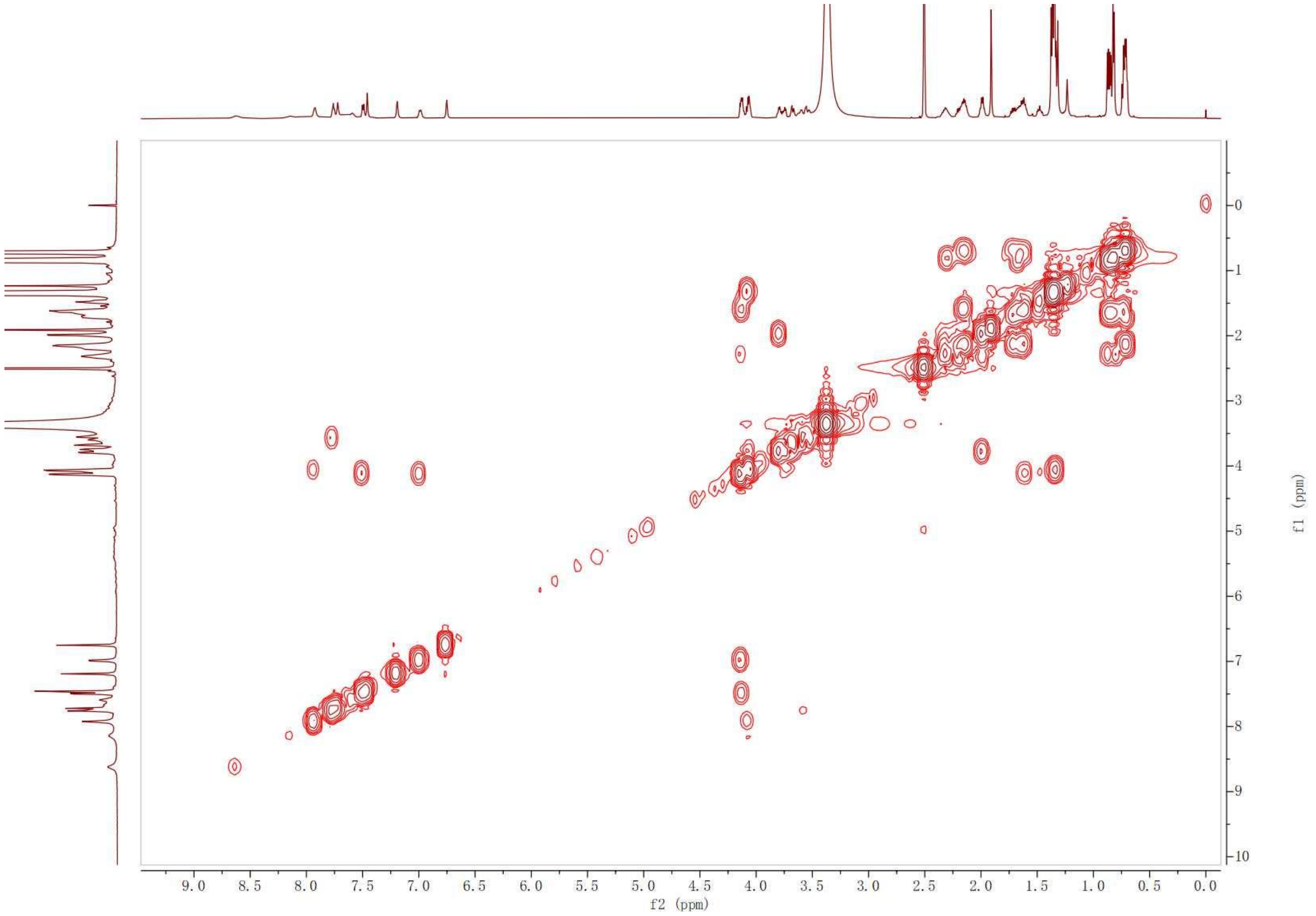

Fig S48. ^{1}H-^{1}H COSY spectrum of compound 5 in DMSO-*d*$_6$ (600 MHz)

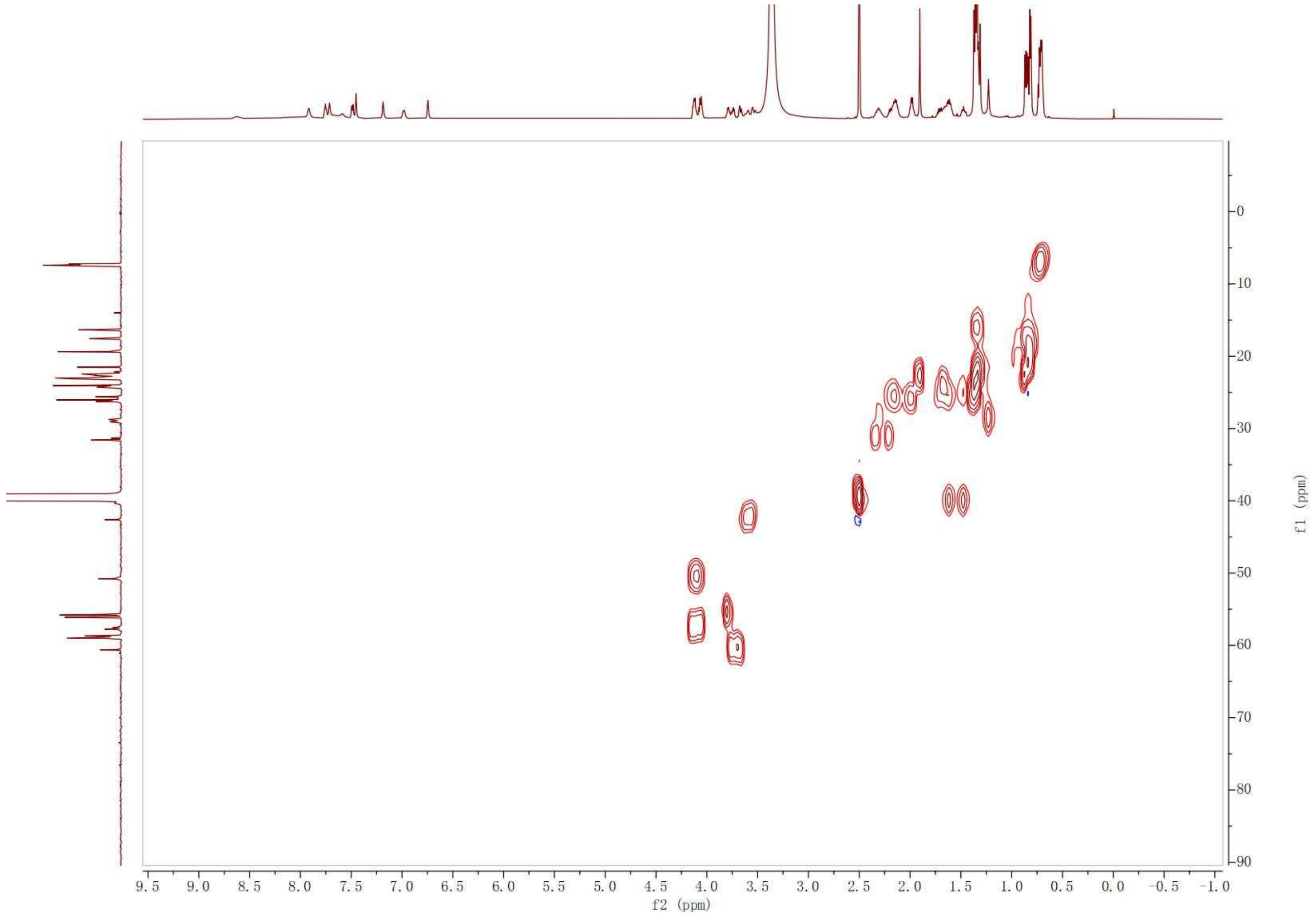

Fig S49. HSQC spectrum of compound 5 in DMSO-d_6 (600 MHz)

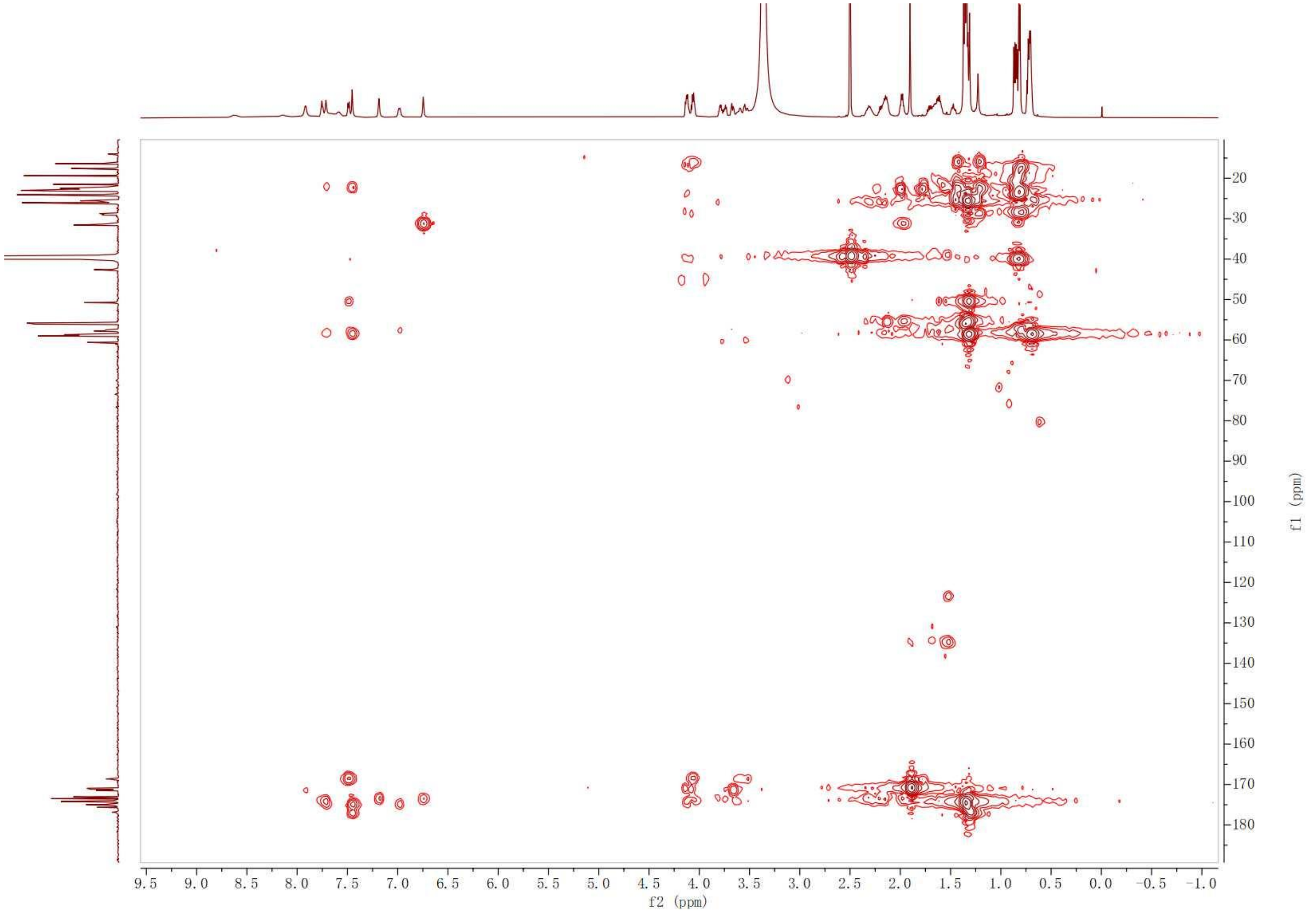

Fig S50. HMBC spectrum of compound 5 in DMSO-d_6 (600 MHz)

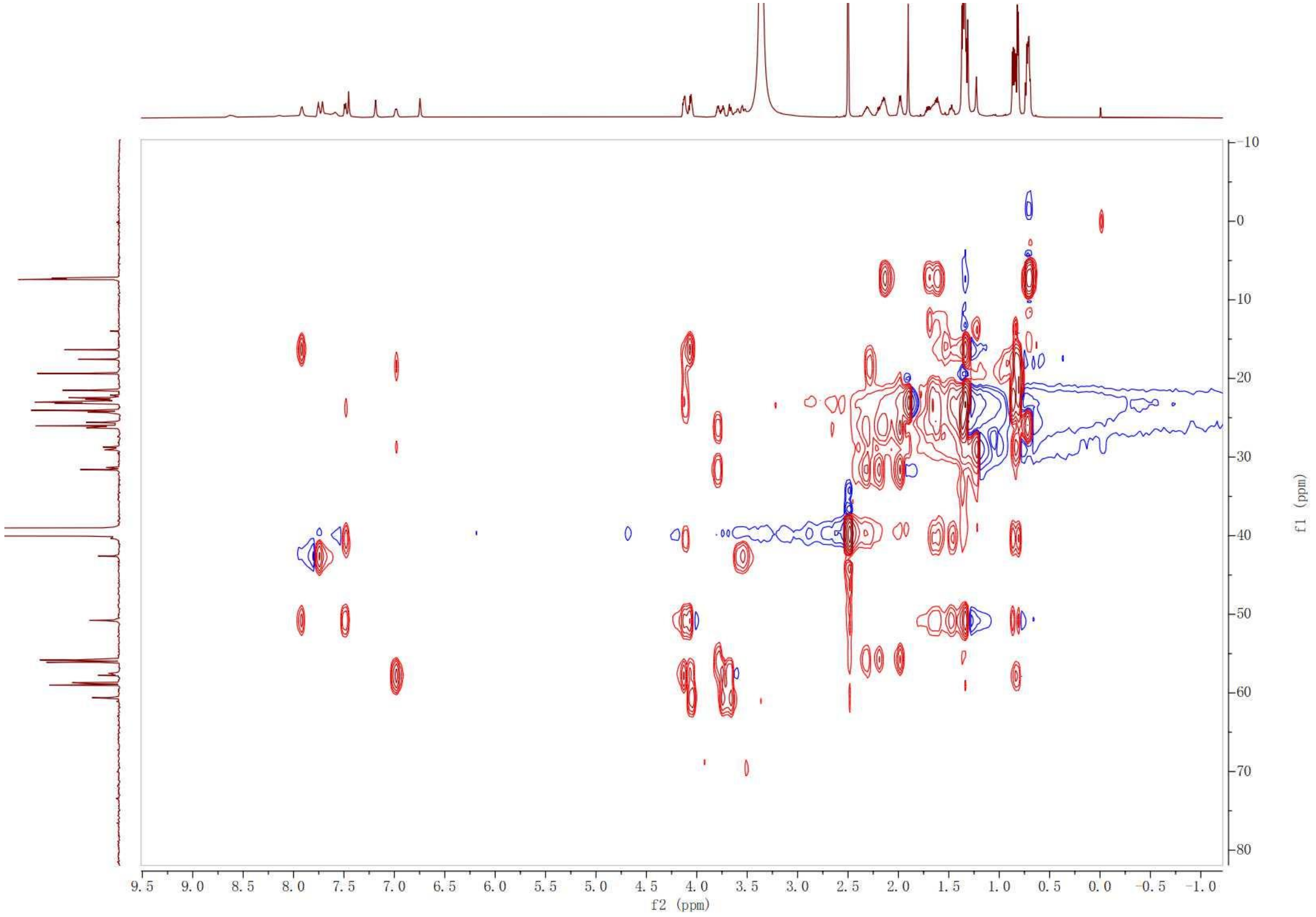

Fig S51. TOCSY spectrum of compound 5 in DMSO-*d*6 (600 MHz)

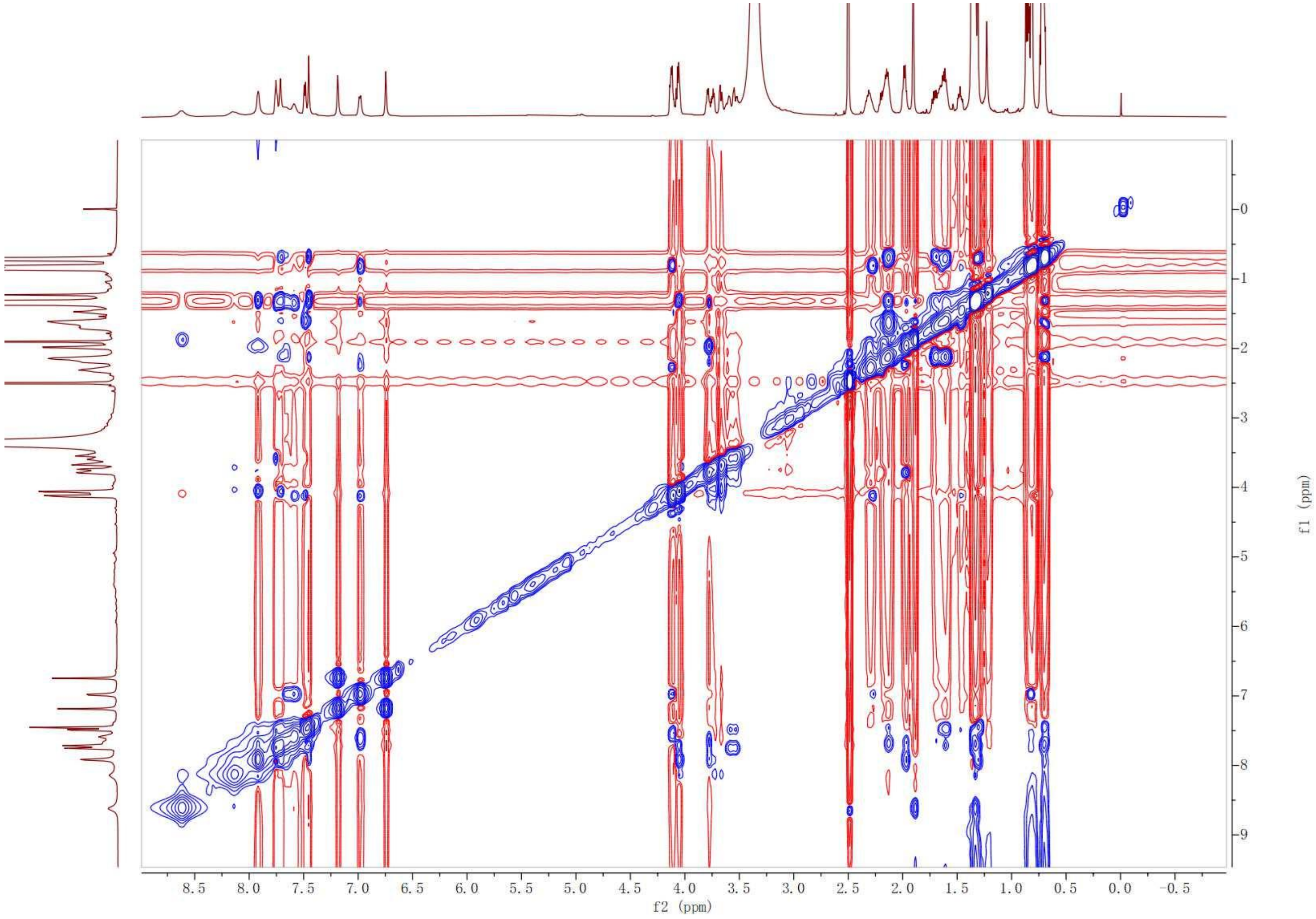

Fig S52. NOESY spectrum of compound 5 in DMSO-*d*$_6$ (600 MHz)

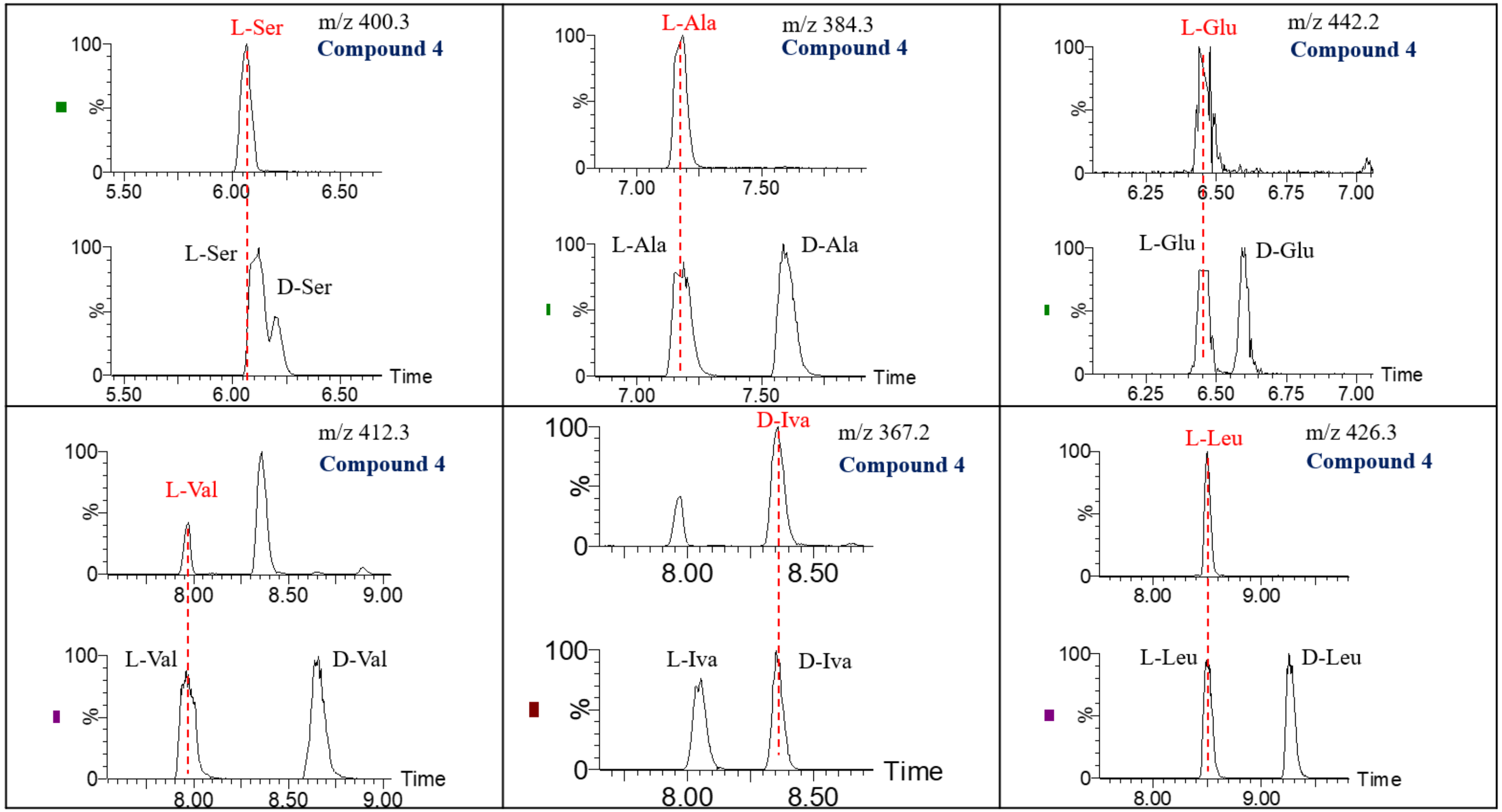

Fig S53. Marfey's of compound 5

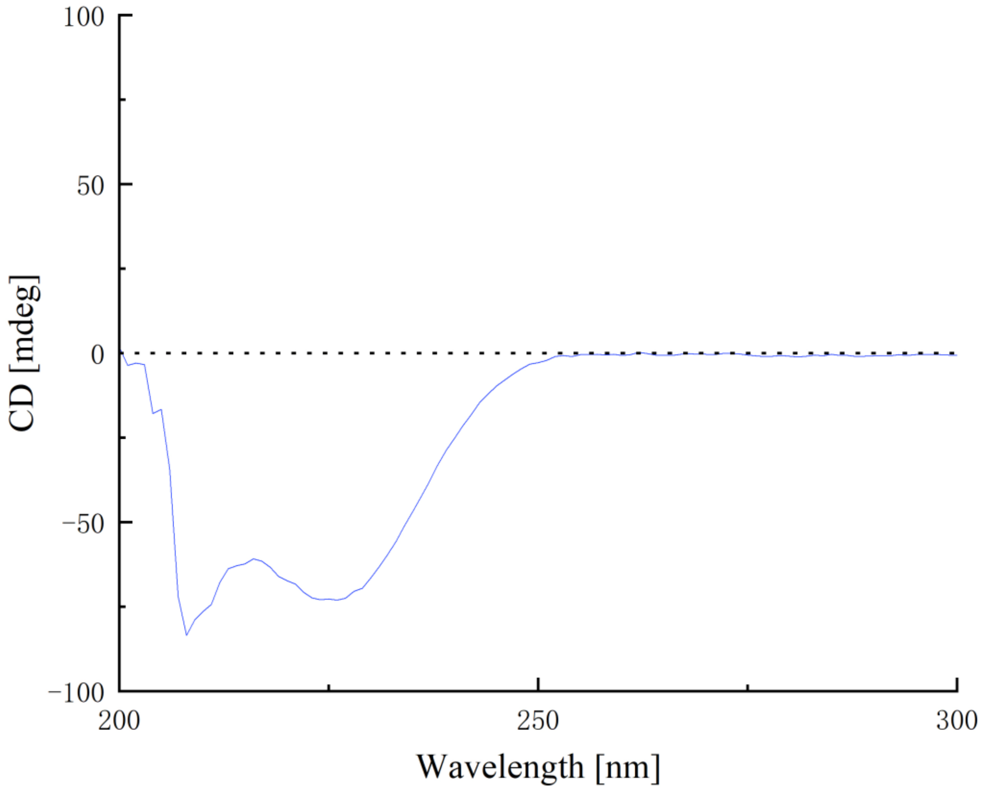

Fig S54. CD spectrum of compound 5 in MeOH

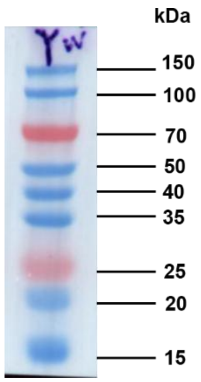

Fig S55. Protein Molecular Weight Marker used in western blotting system to detect Erk1/2 (42 kda).

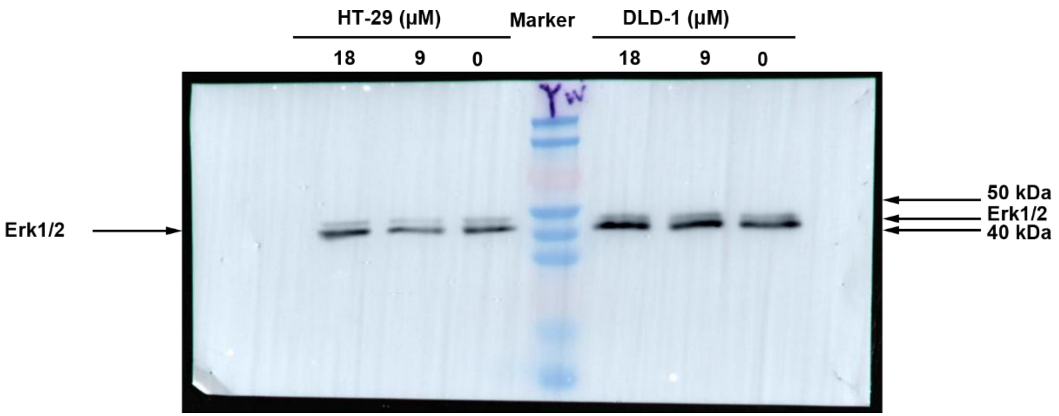

Fig S56. Original protein image of Erk1/2 (42 kda) detected using the Amersham imager 680 QC

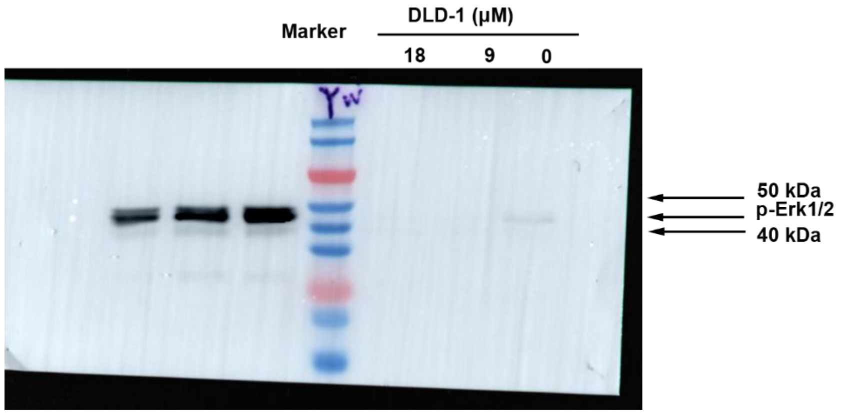

Fig S57. Original protein image of *p*-Erk1/2 (42 kda) in DLD-1 cells detected using the Amersham imager 680 QC.

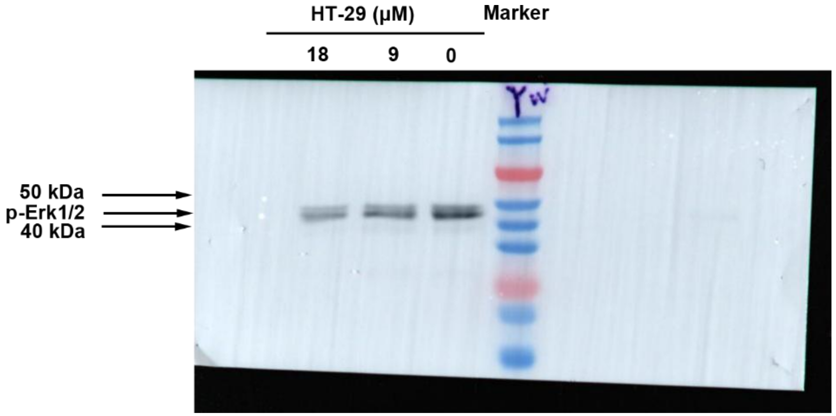

Fig S58. Original protein image of p-Erk1/2 (42 kda) in HT-29 cells detected using the Amersham imager 680 QC

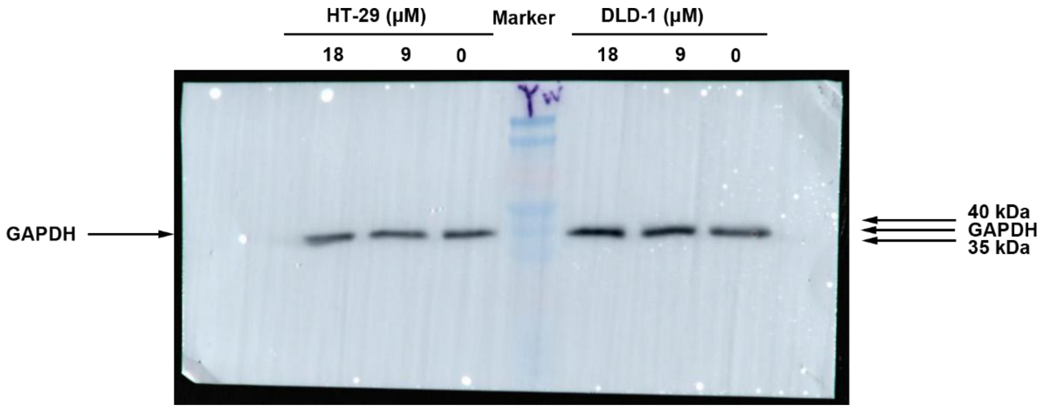

Fig S59. Original protein image of GAPDH (37 kda) detected using the Amersham imager 680 QC
